# Measuring the Quality of Datasets: Development of the IDEFIM Indicator Set for Empirical Health Research

**DOI:** 10.2196/90482

**Published:** 2026-06-17

**Authors:** Sonja Harkener, Oliver J Bott, Christian Draeger, Tobias Hartz, Ekkehart Jenetzky, Matthias Löbe, Stefanie March, Chris Schubert, Jürgen Stausberg

**Affiliations:** 1 Institute for Medical Informatics, Biometry and Epidemiology, Faculty of Medicine, University of Duisburg-Essen, Hufelandstr. 55, Essen, North Rhine-Westphalia, 45122, Germany, 49 201 72377201; 2 Institute for Applied Data Science Hannover (DATA|H), Hochschule Hannover - University of Applied Sciences and Arts, Hannover, Lower Saxony, Germany; 3 Institute for Medical Informatics, Statistics and Epidemiology, University of Leipzig, Leipzig, Saxony, Germany; 4 Clinical Cancer Registry Lower Saxony, Hannover, Germany; 5 Faculty of Health/School of Medicine, Witten/Herdecke University, Witten, North Rhine-Westphalia, Germany; 6 University Medical Center of the Johannes Gutenberg University Mainz, Mainz, Rhineland-Palatinate, Germany; 7 Magdeburg-Stendal University of Applied Sciences, Magdeburg, Saxony-Anhalt, Germany; 8 Library, TU Wien, Vienna, Austria

**Keywords:** dataset, data quality, empirical health research, health care, metadata, quality indicator, secondary use, IDEFIM framework

## Abstract

**Background:**

To be beneficial for empirical health research, a dataset must be fit for use. The quality of a dataset can only be influenced during data collection, yet it is evaluated multiple times during analysis or secondary use by applying quality indicators.

**Objective:**

This study aimed to establish an up-to-date set of indicators measuring the quality of datasets in empirical health research.

**Methods:**

A total of 3 pillars were combined. First, the 51 indicators of a German guideline from 2014 about the management of data quality were revised. Second, a literature review was performed looking for evidence sources since 2013 that describe, propose, or apply dataset quality indicators. Third, indicators were supplemented by a manual search and other sources. The quality indicators were then integrated into the IDEFIM framework. The IDEFIM framework distinguishes between the categories’ data, metadata, context, and openness quality. In this work, only the categories data and metadata quality, with their 14 dimensions were considered.

**Results:**

In total, 69 indicators qualified for the IDEFIM indicator set, 53 related to the category data quality, and 16 to the category metadata quality. A total of 30 indicators originated from the German guideline, 31 from the literature review. Three indicators were added to cover aspects of diversity, equity, and inclusion, and an additional 5 related to specifics of data and metadata quality not addressed so far. Most indicators were found in the dimensions accuracy (data) with 12 measures, completeness (data) with 12 measures, and consistency (data) with 19 measures. According to the number of supporting evidence sources, missing values in data elements (48 evidence sources), contradictions (31), and currentness (26) were the most popular quality indicators. Metadata quality was significantly less frequently addressed.

**Conclusions:**

The presented IDEFIM indicator set can be used for the management of data collections as well as for the verification of a dataset’s quality for an intended use. The indicator set should also be considered in the design of a study in empirical health research and the development of software tools supporting the visualization of issues related to the quality of a dataset.

## Introduction

**Figure 1.**
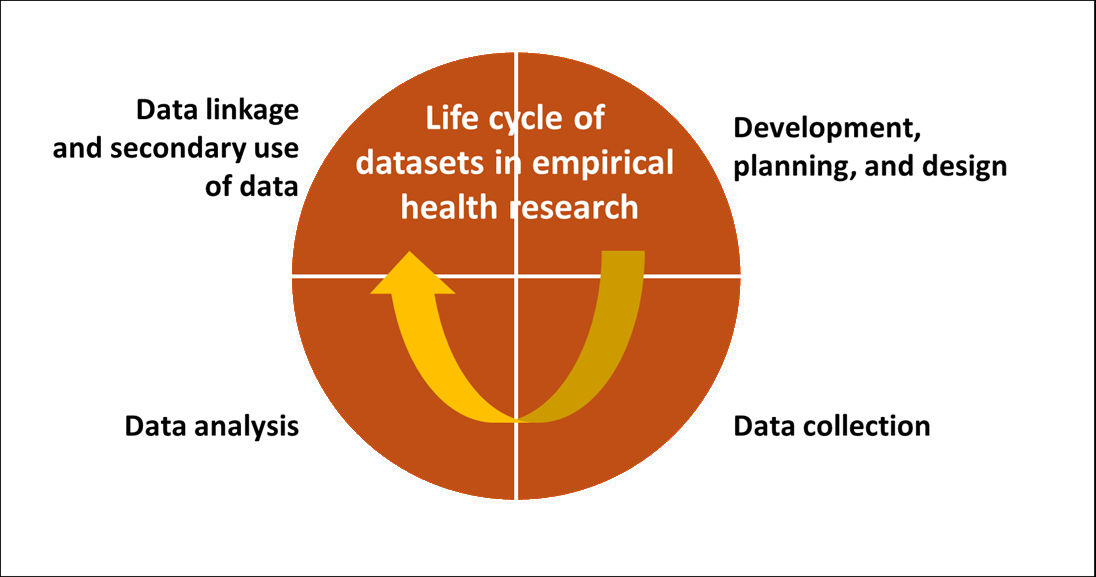
Life cycle of datasets in empirical health research from the development phase to their linkage and secondary use.

The goal of having data that is fit for use accompanies the entire life cycle of empirical health research (Figure 1). It begins with the development of a data collection following a systematic approach as a study [1] or registry protocol [2]. Ideally, predefined research questions guide the determination of populations, samples, visits, and variables, to name a few aspects [3]. It is worthwhile to consider study-specific requirements for characteristics of a dataset, such as case completeness, data completeness, and correctness, already in the development phase, because the design - as well as the available resources - will substantially contribute to fulfilling these requirements. Many recommendations have been made for appropriate designs focusing on different types of empirical health research, for example, CONSORT (Consolidated Standards of Reporting Trials) for randomized trials [4] or STROBE (Strengthening the Reporting of Observational Studies in Epidemiology) for observational research [5].

Project-specific requirements regarding the quality of a dataset can be measured, evaluated, and benchmarked against individual or common thresholds. During data collection, the plan-do-check-act cycle (PDCA cycle) described by Deming [6] can be applied to detect weak points and to implement actions for improvement. The data quality standards of the International Organization for Standardization (ISO) are based upon the approach of active quality management that was brought into practice in the 1950s by United States-Americans such as Crosby [7] and Juran [8]. ISO 8000 defines data quality as the degree to which a set of inherent characteristics of data fulfills requirements [9]. The degree is something measurable that could be quantified using a “data quality measure” introduced in ISO/International Electrotechnical Commission (IEC) 25024 [10]. However, even within ISO standards, there are different definitions of the term “data quality,” each of them reasonable in the respective subject areas. Returning to the PDCA cycle, the measures about the quality of a dataset support the check part within the PDCA cycle, and the consequences determine the act part of the cycle regarding the management and operation of a data collection.

In empirical health research, data collection is usually followed by an analysis phase [11]. Once data collection is complete (with the exception of interim analyses), the design of the data collection could not be changed. The same is true for secondary analyses of data, for example, from electronic health records [12]. Measuring quality is important in the analysis phase to assess whether a dataset is fit for the intended use or for a secondary use. However, reviewing a dataset related to its design, its documentation, its availability, and similar aspects may also be useful. On the one hand, reviews supplement the measurement of characteristics with additional information, such as the FAIRness of a dataset [13]. On the other hand, reviews may be less demanding because access to a dataset is not necessary to evaluate its fitness for use. This advantage of reviews compared to complex measurements could motivate the data quality and utility label proposed for the secondary use of data in the European Health Data Space (EHDS) [14]. However, simply documenting information on data quality does not indicate the certain degree of quality needed, for example, to assess the appropriateness of a dataset with regard to different purposes of empirical health research, such as effectiveness evaluation as the most prominent purpose or hypothesis generation as a basic purpose [15].

New challenges concerning data quality arise from using datasets to generate large language models [16], from the availability of large and uncontrolled volumes of data (“big data”) [17], and from linking datasets either as part of an active data collection or for analysis work [18]. The associated risk of discrimination against vulnerable groups [19] demands a strong consideration of data quality issues.

IDEFIM aims to support the collection and analysis phases of empirical health research by offering an up-to-date list of measures that assess a dataset’s quality. A dataset was defined as a logically meaningful grouping of data [20] and as an “identifiable collection of data available for access or download in one or more formats” [21]. The terminology in the field of data quality was diverse and inconsistent. Therefore, IDEFIM kept the term “quality indicator” from previous work [22] as a synonym for “quality measure” [23]. Quality indicators measure the degree to which requirements concerning characteristics of datasets are met. IDEFIM assumed that the degree to which a dataset possesses a particular characteristic can be quantified by several quality indicators, each focusing on a different aspect of that characteristic. The goal of IDEFIM is to establish a comprehensive and consistent set of quality indicators for empirical health research, embedded in a common framework addressing a dataset’s fitness for purpose and fitness for use. In previous publications, we presented preparatory and supporting work, including our motivation [24], our framework covering dimensions and categories [25], and a structure for specifying a dataset quality indicator [26]. Here, we introduce the IDEFIM indicator set for empirical health research.

## Methods

### IDEFIM Framework

**Figure 2.**
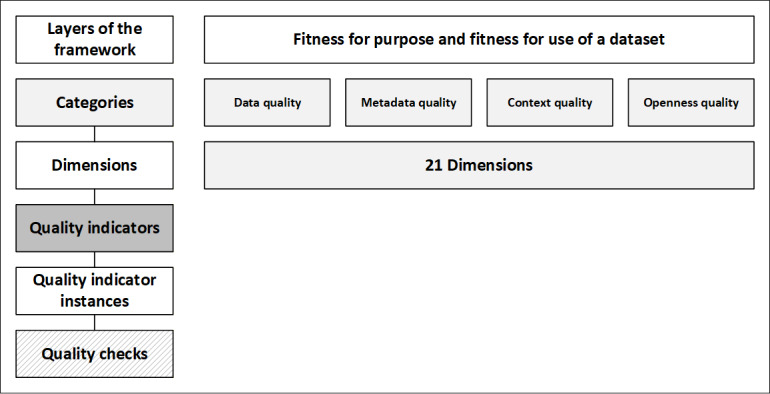
IDEFIM framework with five layers supporting the goal to achieve datasets that are fit for purpose and fit for use.

The IDEFIM framework consists of 4 categories with 21 dimensions (Figure 2 [25]), 8 dimensions in category data quality, 6 in category metadata quality, 3 in category context quality, and 4 in category openness quality. In accordance with ISO 8000, quality indicators measure the degree to which general requirements for characteristics, that is, dimensions, are met. This degree can be used to evaluate the fitness of a dataset for different purposes of empirical health research, such as health services research, quality research, or drug approval research. To evaluate a dataset’s fitness for use in a particular project, quality indicator instances are applied to adjust the quality indicators to the particular setting, such as the recorded data elements. Quality checks represent the algorithms applied to the data. IDEFIM focused on quality indicators and a respective indicator set. However, quality indicators seem not to be appropriate with regard to the categories of context and openness quality. Respective approaches, such as the FAIR Guiding Principles [13] and the 5-star open data approach [27], propose Boolean conditions regarding structures (eg, the existence of an open license as a 1-star prerequisite) and processes (eg, an authentication and authorization procedure to access data as one FAIR Guiding Principle). Boolean conditions are not suitable for calculating measures. Consequently, IDEFIM examined quality indicators only with regard to the categories data and metadata quality.

### Material

#### Overview

The development of the IDEFIM indicator set was based on 3 pillars. First, it builds on national efforts to create a guideline for an adaptive management of data quality in cohort studies and registries [28,29]. Particularly, IDEFIM used indicators published in 2014 under the auspices of the Technology and Methods Platform for Networked Medical Research (TMF), with the second version of this guideline. These TMF indicators were developed through a systematic process over the years based on literature reviews, expert consultations, and community involvement [22]. Second, a broad literature review was conducted in the field of data quality in empirical health research that included the search for evidence sources proposing, describing, or using indicators [24]. This review expanded similar work from the TMF guidelines by searching for publications from 2013 onwards until January 2024. Third, the project identified additional indicators through empirical knowledge of the core project team, manual search looking at relevant journals and conference proceedings, snowball sampling starting with records retrieved during the literature search, and consultations of invited experts in 2 workshops. The first face-to-face workshop with 8 experts took place in November 2024, focusing on the framework. The second face-to-face workshop with 7 experts took place in August 2025, focused on a proposal from the project concerning quality indicators. The workshops were strictly advisory. The core research team made the final decision to include or reject an indicator. The combination of the 3 pillars resulted in a first draft of the set, which explicitly relates each indicator to its origin. This draft was condensed into the final set described here. Each indicator in the final set was described using a uniform structure [26].

#### TMF Indicators

The guideline contains 51 indicators organized into 3 categories: integrity, organization, and trueness. These indicators are defined in a structured way using 14 items such as sources, calculation, and interpretation. As IDEFIM was concerned with empirical research projects on a general level, 10 of these indicators were excluded because they were either cancer registry specific or concerned measurements and their conditions that particularly occur in cohort studies (Multimedia Appendix 1). Thus, 41 indicators remained for IDEFIM from the TMF guideline. Each indicator was then assigned to exactly 1 dimension. Names and definitions of the indicators were revised and extended as necessary to create a consistent and up-to-date set of quality indicators.

#### Literature Search

The literature search included Medline, the Cochrane Library, the Web of Science, and Scopus [24]. It was conducted on February 2, 2024, for Medline and on January 16, 2024, for the other 3 information sources. The search looked at English or German records published since January 1, 2013. The search criteria were tailored to the individual options offered by the 4 information sources (Multimedia Appendix 2). The results were merged, and duplicates were eliminated, resulting in 2748 records.

Due to the large volume of records, the screening based on titles and abstracts was split into 2 stages. A prescreening focused on data quality as a topic, the relationship to empirical health research, and general relevance. The records were divided equally among 2 reviewers. The prescreening took place between February 9, 2024, and March 6, 2024. The remaining 734 records were evaluated independently by 2 reviewers (SH and JS) between March 12, 2024, and April 8, 2024. The relationship of a record to the topic of data quality could be further differentiated into the following aspects: the structure for the description of data quality indicators, indicators of data quality, dimensions of data, gender equality and diversity in relation to data quality, and other references to data quality. An optional comment could be entered for each search result. In case of disagreement between the reviewers, a consensus was reached, resulting in 221 records.

Out of the 221 search results, 219 were obtained in full text. Here as well, due to the unexpectedly high number of remaining records, the search results were randomly divided in half and evaluated using the following criteria: type of scientific project (eg, analysis of data quality), reference to empirical research projects in medicine (eg, registry), reference to data quality (eg, indicators for data quality), and recommendation for further consideration. This left 117 sources of evidence for the review. The PRISMA (Preferred Reporting Items for Systematic Reviews and Meta-Analyses) flow diagram is presented in Multimedia Appendix 3. Table 1 shows the distribution of the 117 sources with regard to aspects of data quality.

**Table 1.** Distribution of the 117 evidence sources with regard to aspects of data quality (multiple options possible).

Category	Frequency
Dimensions of data	101
Quality indicators	79
Standards of data quality	28
Special statistical procedures	21
Overall score for data quality	19
Interventions to improve data quality	18
Quality checks	15
Structure of quality indicators	9
Quality of metadata and paradata	6
Gender equality and diversity with reference to data quality	4
Other reference to data quality	28

#### Literature Review

The literature search yielded 117 relevant evidence sources, out of which 79 specifically related to data quality indicators. Each evidence source was examined by one reviewer (SH) for possible indicators, indicator instances, and quality checks. Due to the diverse and inconsistent terminology of measures, indicator instances, and quality checks were considered at this stage. Respective findings were recorded for each evidence source. However, some sources yielded no findings. In these cases, the entire source was recorded as a single finding. This resulted in a total of 622 findings, ie, candidates for quality indicators, from 79 evidence sources. A total of 2 reviewers (JS and SH) assessed each finding independently. If a finding corresponded to one of the 41 considered TMF indicators, it was marked accordingly. If no assignment made sense, either a “new indicator,” “not applicable,” or “undecidable” flag could be set. Proposals for new indicators were mainly considered in relation to the categories of data and metadata quality. It was assumed that requirements concerning context and openness quality are better defined using Boolean conditions than measures. Table 2 shows the results of this stage. The reviewers agreed on 265 findings, and they disagreed on 357 findings.

**Table 2.** Initial evaluation of quality indicator findings by two reviewers (JS and SH).

Option	Frequency
Both reviewers performed a consistent mapping to a TMF^a^ indicator	174
Both reviewers performed a mapping to a TMF indicator, but it was different	131
One reviewer performed a mapping to a TMF indicator; the other did not	152
Both reviewers unanimously opted for “new indicator”	43
Both reviewers unanimously opted for “not applicable”	48
Other scenarios, eg, combinations of new, not applicable, and undecidable	74

a TMF: Technology and Methods Platform for Networked Medical Research.

Both reviewers discussed the 357 disagreements and reached a consensus. Findings that were not considered proposals for an indicator, an indicator instance, or a quality check were deleted. Findings with suggestions for mapping to 2 or 3 indicators were broken down into individual entries leading to a numerical growth. This left 74 evidence sources with a total of 624 findings: 99 findings proposing a new indicator, 428 findings with a successful mapping to a TMF indicator, and 97 findings rejected. The median number of findings per source was 5.5 (range 1-54) findings. A total of 30% (187/624) of the findings came from 6 evidence sources [30-35]. Findings qualifying for a new indicator were combined based on the description in the respective evidence sources. Each new indicator was assigned to one of the 14 dimensions in the categories data and metadata quality. A Microsoft Access database was used for the literature review, including the management of the retrieved records, the reviews, and the analysis of agreement.

#### IDEFIM Contributions

Proposals for new indicators beyond the literature review were permitted to maximize the use of community knowledge, even if the proposals were published more recently than the literature search. These proposals were identified through snowball sampling from evidence sources, a manual search of other publications proposing quality indicators, input from scientific conferences, and contributions from invited experts. According to the project work plan, the relationship between data quality and the concepts of diversity, equity, and inclusion (DEI) was examined based on the initial records from the literature search [36]. Related indicators were also established and added to the indicator set.

### Condensation of the Draft Indicator Set

Only indicators assigned to a dimension in categories data and metadata quality were accepted. According to the IDEFIM project protocol, TMF indicators without evidence from at least 1 independent source in the literature review were excluded from the final indicator set. Furthermore, the draft set was screened for possible merges. In addition to the originating pillar - TMF work, literature review, and IDEFIM contributions - the indicator proposals were not formally and empirically validated.

### Ethical Considerations

This research did not involve human participants.

## Results

The draft set included 81 indicators. A total of 6 initially kept TMF indicators were excluded from the draft set because they were not referenced by an evidence source. Additionally, 4 TMF indicators were defined as subconcepts of 2 other indicators using information expected on the level of indicator instances. These 4 indicators were also excluded (Multimedia Appendix 1). A total of 2 indicators, 1 TMF indicator and 1 indicator from the review, were skipped because they refer to a dimension in the category context quality. Table 3 shows the distribution of the final set of 69 indicators. Of the 41 TMF indicators, 30 remained after condensation, all dealing with data quality. A total of 31 new indicators were added based on findings from the literature review. These 31 new indicators were distributed fairly evenly between both categories, data and metadata quality. While the total number of indicators was lower in category metadata quality, they were distributed fairly evenly across its dimensions. In contrast, the dimensions of the category data quality were unevenly covered by indicators. Accuracy (data), completeness (data), and consistency (data) were the most dominant here. The 8 indicators added by IDEFIM did not significantly change the distribution.

**Table 3.** Distribution of the 69 indicators among the 14 dimensions in the data and metadata quality categories of the IDEFIM indicator set.

Category and dimension	Origin			
	Total	TMF^a^	Literature review	IDEFIM contribution
Data quality	53	30	16	7
Accuracy (data)	12	6	4	2
Completeness (cases)	3	2	—^b^	1
Completeness (data)	12	7	3	2
Compliance (data)	3	—	3	—
Consistency (data)	19	13	5	1
Credibility	1	—	1	—
Currentness	1	1	—	—
Representativeness	2	1	—	1
Metadata quality	16	0	15	1
Accuracy (metadata)	2	—	2	—
Completeness (metadata)	4	—	4	—
Compliance (metadata)	2	—	2	—
Consistency (metadata)	4	—	3	1
Precision	2	—	2	—
Understandability	2	—	2	—
Total	69	30	31	8

a TMF: Technology and Methods Platform for Networked Medical Research.

b Not available.

Table 4 lists 53 indicators related to data quality organized into 16 indicator groups for clarity, with 1 to 4 groups per dimension. The 16 indicators contributed by the literature review originated from 26 evidence sources. IDEFIM added 3 indicators based on a separate analysis of DEI issues [36]. Additionally, James et al [37] propose the R-index as a standardized representativeness metric for benchmarking DEI in a dataset. The R-index is listed in the IDEFIM indicator set as “Conspicuous representativeness distribution.” To complete the consideration of the contingency table [38,39], the F-Measure was defined as the harmonic mean of correctness and recall based on a recommendation from the second workshop and named “Validity.” Time series, for example, for lab values, are a special phenomenon in health-related datasets. The idea of Giesa et al [40] to address this phenomenon with an indicator that provides a rate of incomplete time series was accepted. While homonyms of observational units were considered in the TMF guideline, homonyms of pieces of information were missing and supplemented according to Woodall et al [41].

**Table 4.** Indicators related to data quality with origin and number of supporting evidence sources (ES).

Dimension, indicator group, and indicator		Origin	Number of ES^a^
Accuracy (data)			
Contingency table indicators			
Conspicuous correctness distribution		IDEFIM^b^ (DEI^c^)	—^d^
Correctness		TMF^e^	18
Recall		TMF	6
Validity		IDEFIM	—
Disagreement with source data indicators			
Disagreement with source data referring to data elements		TMF	21
Disagreement with source data referring to observational units		TMF	1
Illegal content indicators			
Illegal values of qualitative data elements		TMF	11
Illegal values of qualitative data elements used for the coding of missings		TMF	4
Incorrect text in qualitative data elements		Review	4
Misfielded values		Review	3
Other indicators for accuracy (data)			
Granularity (data)		Review	2
Temporal trends in counts or proportions		Review	1
Completeness (cases)			
Indicators for completeness (cases)			
Conspicuous recruitment rate distribution		IDEFIM (DEI)	—
Drop-out-rate		TMF	6
Recruitment rate		TMF	15
Completeness (data)			
Missing content indicators			
Conspicuous missing values distribution		IDEFIM (DEI)^c^	—
Missing modules		TMF	5
Missing values in data elements		TMF	48
Temporal missingness		IDEFIM [40]	—
Other indicators for completeness (data)			
Data elements with existing entries for all observational units		TMF	2
Information density score		Review	1
Modules with existing entries for all data elements		Review	1
Observational units with existing entries for all data elements		Review	4
Observational units with follow-up		TMF	1
Refusal rate indicators			
Refusal rate of investigations		TMF	1
Refusal rate of modules		TMF	1
Refusal rate of single data elements		TMF	1
Compliance (data)			
Indicators for compliance (data)			
Data format, data type, and unit compliance		Review	13
Incompliance with metadata		Review	1
Range compliance		Review	8
Consistency (data)			
Confusion and redundancy indicators			
Confusion		IDEFIM [41]	—
Duplicates (data)		TMF	13
Homonyms (data)		TMF	2
Synonyms (data)		TMF	8
Contradiction indicators			
Contradictions		TMF	31
Data element contradictions		Review	2
Other indicators for consistency (data)			
Missing evidence of known correlations		TMF	4
Single data source per observational unit		TMF	2
Temporality of categorical data elements		Review	1
Unexpected entry indicators			
Concordance		TMF	14
Conspicuous distribution of digits in date-time data elements		Review	1
Conspicuous distribution of values		TMF	6
Data elements with value unknown etc		TMF	3
Disagreement with previous values		TMF	3
Frequency outliers		Review	1
Last digit preferences		TMF	2
Outliers (continuous data elements)		TMF	17
Outliers in numerical data elements in a multivariate analysis		Review	1
Values from external references		TMF	3
Credibility			
Indicators for credibility			
Data element credibility		Review	2
Currentness			
Indicators for currentness			
Currentness		TMF	26
Representativeness			
Indicators for representativeness			
Conspicuous representativeness distribution		IDEFIM [37]	—
Representativeness		TMF	7

a ES: evidence source.

b IDEFIM: contributed by the project, not the literature review.

c DEI: diversity, equity, and inclusion.

d Not available.

e TMF: Technology and Methods Platform for Networked Medical Research.

The number of supporting evidence sources derived from the literature review can be used to weight indicators against each other. A total of 3 indicators were affected by more than one-third of the 74 evidence sources: “Missing values in data elements” (48 evidence sources), “Contradictions” (31), and “Currentness” (26). Two additional indicators were supported by at least a quarter of the evidence sources: “Correctness” (18) and “Disagreement with source data referring to data elements” (21). Multimedia Appendix 4 gives a list of all evidence sources for each indicator in the category data quality.

Table 5 lists the 16 indicators related to metadata quality. Because there are fewer indicators, there is only 1 indicator group per dimension. The 15 indicators from the literature review came from 10 evidence sources. IDEFIM added 1 indicator: “Duplicates” refers to a comparison of metadata between multiple datasets, that intentionally do not overlap [41]. This indicator counts redundantly defined data elements. In the category metadata quality, the number of supporting evidence sources was less suitable as an indicator weight. Only 2 indicators had more than 1 supporting evidence source, “Metadata format, type, and unit compliance” (3 evidence sources) and “Granularity (metadata)” (2). Multimedia Appendix 5 provides a list of all evidence sources for each indicator in the category metadata quality.

**Table 5.** Indicators related to metadata quality with origin and number of supporting evidence sources (ES).

Dimension, indicator group, and indicator	Origin^a^	Number of ES^b^
Accuracy (metadata)		
Indicators for accuracy (metadata)		
Correctness (metadata)	Review	1
Responsiveness	Review	1
Completeness (metadata)		
Indicators for completeness (metadata)		
Completeness of administrative metadata	Review	1
Coverage of all data elements	Review	1
Data element completeness	Review	1
Richness (metadata)	Review	1
Compliance (metadata)		
Indicators for compliance (metadata)		
Data element compliance with reference	Review	1
Metadata format, type, and unit compliance	Review	3
Consistency (metadata)		
Indicators for consistency (metadata)		
Duplicates (metadata)	IDEFIM [41]	—^c^
Heterogeneous representation of data elements	Review	1
Homonyms (metadata)	Review	1
Synonyms (metadata)	Review	1
Precision		
Indicators for precision		
Granularity (metadata)	Review	2
Residual classes of qualitative data elements	Review	1
Understandability		
Indicators for understandability		
Easy of understanding	Review	1
Relevance of the dataset’s descriptive information	Review	1

a IDEFIM: contributed by the project, not the literature review.

b ES: evidence source.

c Not available.

## Discussion

### Principal Findings

With 69 indicators, the IDEFIM indicator set expanded its predecessor, the TMF guideline [22], by one-third. The 2 most comprehensive indicator lists found in the literature review contributed 54 [33] and 35 findings [34]. ISO/IEC 25024 offers 63 quality measures [10]. Considering IDEFIM’s coverage of data as well as metadata quality, 69 indicators seem manageable compared to potential competitors. However, the IDEFIM set does not cover the categories context and openness quality. Multimedia Appendix 6 contains the specifications of each indicator.

As expected, not all TMF indicators were confirmed. However, the final set impressively demonstrated the relevance of the adopted indicators. The 8 most frequently mentioned indicators originated from the TMF guideline. Conversely, 21 out of 31 indicators added by the review were mentioned only once. In the category metadata quality, both the number of indicators was lower and the supporting evidence was weaker than in the category data quality. This does not necessarily mean that metadata quality was out of scope. Metadata quality might be appropriately evaluated with a mixture of measures (as is the case in the category data quality) and conditions (as it might be the case in the categories context and openness quality). Nevertheless, indicators of metadata quality could be a critical area for future work to effectively publish open data from empirical health research [42].

The IDEFIM indicator set and its framework provide a foundation for measuring the quality of datasets in empirical health research. Further work could lead to a community approach of sharing and harmonizing instances and applications of its indicators. This includes recommendations for using the indicators, indicator instances, and quality checks in specific use cases. The cross-registry benchmarking of data in health services research can serve as an initial example here [43]. The quality indicators were taken from the TMF guideline. Each participating registry then tailored the indicators to its own setting by specifying data elements considered with regard to missing values, determining its particular denominator with regard to the recruitment rate, and defining the rules used to count contradictions, for example. This tailoring resulted in quality indicator instances for each registry. However, the quality indicators’ common ground with identical meanings and thresholds allowed for a comparison of results between registries. Due to the registries’ different information models, data structures, and concept systems, each registry was responsible for specifying and implementing quality checks.

### Comparison With Prior Work

Like others, IDEFIM understood a quality indicator as a distinct entity, broken down into components in its specification. Woodall et al [41] took a different approach. They distinguish between 9 data quality problems, 9 generic data quality methods, and 6 so-called taxonomy elements. Taxonomy elements combine different objects of a data structure from the perspective of a relational database management system. Quality measures arise from the cross-tabulation of data quality problems and taxonomy elements, which involves assigning none, 1, or several generic data quality methods to each intersection point. Of the initial 54 intersection points, 16 were left blank as senseless and 6 were labeled as gaps that could be filled in subsequent work. The remaining 32 intersection points were filled with 57 entries. To address all aspects proposed by Woodall et al [41], IDEFIM added 2 indicators. “Duplicates (metadata)” represented the cross-domain analyses mentioned by Woodall et al [41] at the level of data elements intended to reduce redundancy. Second, IDEFIM supplemented the data quality problem “Existence of synonyms and homonyms” with the indicator “Confusion,” which examines homonyms of values in a dataset. The approach of Woodall et al [41] turned out to be a good training partner for IDEFIM; however, constructing indicators by combining different axes appeared too theoretical for practical use.

QUANTUM proposes a data quality and utility label [44] that might be used in the EHDS according to article 78 of the respective regulation [14]. In this regulation, a “data quality and utility label means a graphic diagram, including a scale, describing the data quality and conditions of use of a dataset.” This concept is closely related to measuring dataset quality with the IDEFIM framework. However, there is a fundamental difference between QUANTUM and IDEFIM. QUANTUM examines the documentation of quality-related aspects, while IDEFIM examines quality-related aspects directly. In combination, IDEFIM might constitute the basis from which QUANTUM derives its rating. Table 6 shows a comparison between QUANTUM and IDEFIM. Most of QUANTUM’s measure labels represent Boolean conditions that do not provide information about data or metadata quality, for example, the “availability of a data access & usage policy at the time of release of the dataset”. Some measure labels represent measurable quantities such as the coverage rate. For this comparison, we assigned IDEFIM indicators to a measure label even if the label represents a Boolean condition. In these cases, the indicator can quantify the fulfillment of a condition or evaluate the fulfillment of a condition based on a predefined threshold.

**Table 6.** Comparison between the measures of QUANTUM [44] and IDEFIM.

QUANTUM		IDEFIM
Dimension	Measure label	Reference
Accessibility	Availability of a data access & usage policy at the time of release of the dataset Average time from data access application to data release for a speciﬁc dataset	Category: openness quality Dimension: availability Category: openness quality Dimension: accessibility
Population coverage	Coverage Rate (percentage of the eligible population represented in the dataset)	Category: data quality Dimension: completeness (cases) Quality indicator: “Recruitment rate”
Population representativity	How closely does the observed population represent the expected population?	Category: data quality Dimension: representativeness Quality indicator: “Representativeness”
Compliance	Is there documentation of compliance with ethical standards, conventions, protocols or regulations?	Category: context quality Dimension: compliance (context)
Data provenance	Is the source of the dataset documented?	Category: context quality Dimension: provenance
	Are the processes and operations on the data documented?	[not addressed]
Metadata scope	Existence of comprehensive standardised metadata	Category: metadata quality Dimension: completeness (metadata) Quality indicator: “Richness (metadata)”
	Existence of an exhaustive data dictionary at variable level	Category: metadata quality Dimension: completeness (metadata) Quality indicator: “Coverage of all data elements”
Accuracy	Is the accuracy of the dataset documented?	Category: data quality Dimension: accuracy (data) Quality indicator: [several]
Coherence	Is coherence of the dataset documented?	Category: data quality Dimension: accuracy (data) Group of indicators: illegal content Quality indicator: [several]
Completeness	Is completeness of the dataset documented?	Category: data quality Dimension: completeness (data) Quality indicator: [several]
Consistency	Is the consistency of the dataset documented?	Category: data quality Dimension: consistency (data) Quality indicator: [several]
Precision	Is the precision of the dataset documented?	Category: metadata quality Dimension: precision Quality indicator: “Granularity (metadata)”
Validity	Availability of a conformance report for the data model	Category: data quality Dimension: compliance (data) Quality indicator: [several]

From the comparison, one can conclude that IDEFIM addressed all measures of QUANTUM with quality indicators, if the categories data quality and metadata quality are concerned. Therefore, using the IDEFIM indicator set will simplify the registration for the EHDS data quality and utility label to this extent. However, the terminology of QUANTUM differs and does not refer to ISO standards, terms, or definitions. This can be attributed to the unusual framework and wording of the EHDS regulation.

### Limitations

Some limitations of the work must be noted. First, the variety of terminologies used to define data quality and its dimensions posed a challenge. For instance, we found the terms aspects, attributes, categories, characteristics, domains, features, indicators, metrics, processes, and submetrics to be synonyms or siblings of the term “dimension.” The same was true for the term “indicator” noted, for example, as measure, check, or even dimension. In cases of doubt, the criteria were interpreted generously that may have led to misleading sources. Second, maintaining the focus on empirical health research was difficult. This dilemma demonstrates the importance of publishing terms in a citable manner to avoid ambiguity in interpretation and to encourage reliable reuse. Substantial methodological contributions to data quality originated from other fields, even outside of health care. We did not want to overlook important proposals for quality indicators, so we accepted some sources from outside the domain of empirical health research. The domain addressed in the literature was sometimes unclear or overlapped with empirical health research. Particularly, the secondary use of data raises awareness about the initial quality of health-related data, that is, the quality of data from daily health care. Sources were also accepted outside the intended domain if their approach appeared relevant. Third, IDEFIM does not reflect solely the result of its literature review. Although IDEFIM considered the PRISMA extension for Scoping Reviews [45], it uses the results of the literature review as one but not the only basis for its indicator set. However, it would not have been possible to develop a relevant indicator set alone from the literature review due to the terminological but also conceptual confusion. Therefore, the IDEFIM indicator set offers a unique perspective. The bottom-up approach that gives evidence to each indicator is a strength and a unique selling point. Fourth, it is important to note that formal consensus mechanisms were not applied to all parts of this work. For instance, the literature review was one but not the only pillar that led to the inclusion of indicator proposals in the IDEFIM indicator set. Ultimately, the IDEFIM indicator set reflects the decisions of the core project team. Furthermore, a formal and empirical validation of individual indicators was outside the scope of the presented work. Whenever possible and appropriate, this validation is a project of its own for each indicator.

### Conclusions

IDEFIM offers the most up-to-date set of indicators intended to measure the quality of a dataset in empirical health research, as far as the authors know. This set can be used to manage and control the data collection in a clinical trial, a cohort study, or a patient registry, for example. Furthermore, datasets can be labeled according to their fitness for purpose in principle. Then, health data users can verify electronic health data or claims data with respect to the intended use. All indicators of the IDEFIM set were defined using a uniform structure and integrated into the IDEFIM framework of categories and dimensions. However, there is no automatism that allows for the application of the indicators from scratch. It is up to the users to adapt quality indicators to their specific needs, eg, data elements of interest, to create respective indicator instances, and to implement quality checks operating on the data representation layer. This work does not oppose tool-based approaches to data quality control, such as the DataQualityDashboard [46] and dataquieR [47]. Rather, it offers a conceptual basis for the appropriate application of related tools in empirical health research practice.

## Supplementary material

10.2196/90482 Multimedia Appendix 1 Original set of TMF indicators.

10.2196/90482 Multimedia Appendix 2 Search strings used for Medline, the Cochrane Library, the Web of Science, and Scopus.

10.2196/90482 Multimedia Appendix 3 PRISMA flow diagram.

10.2196/90482 Multimedia Appendix 4 Evidence sources per indicator in category data quality.

10.2196/90482 Multimedia Appendix 5 Evidence sources per indicator in category metadata quality.

10.2196/90482 Multimedia Appendix 6 Individual indicator specifications in version 0.8.

## Abbreviations

CONSORT: Consolidated Standards of Reporting Trials
DEI: diversity, equity, and inclusion
DFG: Deutsche Forschungsgemeinschaft
EHDS: European Health Data Space
ES: evidence source
IEC: International Electrotechnical Commission
ISO: International Organization for Standardization
PDCA: plan-do-check-act
PRISMA: Preferred Reporting Items for Systematic Reviews and Meta-Analyses
STROBE: Strengthening the Reporting of Observational Studies in Epidemiology
TMF: Technology and Methods Platform for Networked Medical Research
