# Supplementary material for: Measuring the Quality of Datasets: Development of the IDEFIM Indicator Set for Empirical Health Research
Source: J Med Internet Res. 2026 Jun 17;28:e90482. doi: 10.2196/90482 (PMC13274964; doi:10.2196/90482)
Supplement: Multimedia Appendix 1 [file jmir-v28-e90482-s001.docx]

Original Set of TMF Indicators

| **TMF indicator** | | **Implementation in the IDEFIM set^&^** |
| --- | --- | --- |
| **Category/ID** | **Designation^#^** |  |
| **Integrity** |  |  |
| TMF-1001 | Disagreement with previous values | Final set: IDEFIM-1001 |
| TMF-1002 | Concordance | Final set: IDEFIM-1002 |
| TMF-1003 | Contradictions | Final set: IDEFIM-1003 |
| TMF-1035 | Endless survivor | Excluded before SR: Covered by IDEFIM-1036 |
| TMF-1004 | Certain contradictions | QI Instance of IDEFIM-1003 |
| TMF-1005 | Possible contradictions | QI Instance of IDEFIM-1003 |
| TMF-1006 | Conspicuous distribution of values | Final set: IDEFIM-1006 |
| TMF-1007 | Last digit preferences | Final set: IDEFIM-1007 |
| TMF-1009 | Distribution of parameters recorded by the investigator | Excluded before SR: Covered by IDEFIM-1006 |
| TMF-1010 | Distribution of parameters recorded by the device | Excluded before SR: Covered by IDEFIM-1006 |
| TMF-1011 | Distribution of findings recorded by a medical reader | Excluded before SR: Covered by IDEFIM-1006 |
| TMF-1052 | Distribution of parameters between study sites | Excluded before SR: Covered by IDEFIM-1006 |
| TMF-1008 | Medical tests on special days | No match in the SR |
| TMF-1012 | Missing modules | Final set: IDEFIM-1009 |
| TMF-1013 | Missing values in data elements | Final set: IDEFIM-1010 |
| TMF-1014 | Missing values in mandatory data elements | QI Instance of IDEFIM-1010 |
| TMF-1015 | Missing values in optional data elements | QI Instance of IDEFIM-1010 |
| TMF-1016 | Data elements with value unknown etc. | Final set: IDEFIM-1013 |
| TMF-1017 | Data elements with existing entries for all observational units | Final set: IDEFIM-1014 |
| TMF-1018 | Outliers (continuous data elements) | Final set: IDEFIM-1015 |
| TMF-1019 | Values that exceed measurement limits | Excluded before SR: Covered by IDEFIM-1015 |
| TMF-1020 | Values from external references | Final set: IDEFIM-1016 |
| TMF-1021 | Illegal values of qualitative data elements | Final set: IDEFIM-1017 |
| TMF-1022 | Illegal values of qualitative data elements used for the coding of missings | Final set: IDEFIM-1018 |
| TMF-1023 | Illegal values used for the coding of missing modules | No match in the SR |
| TMF-1024 | Illegal values of quantitative data elements used for the coding of results exceeding measurement limits | Excluded before SR: Covered by IDEFIM-1058 |
| TMF-1025 | Data elements with unspecific values | No match in the SR |
| TMF-1026 | Observational unit with unknown primary tumor | Excluded before SR: Covered by IDEFIM-1013 |
| TMF-1027 | Missing evidence of known correlations | Final set: IDEFIM-1022 |
| TMF-1050 | Coverage of metadata from investigations | No match in the SR |
| **Organization** |  |  |
| TMF-1028 | Currentness | Final set: IDEFIM-1024 |
| TMF-1029 | Duplicates (data) | Final set: IDEFIM-1025 |
| TMF-1030 | Recruitment rate | Final set: IDEFIM-1026 |
| TMF-1051 | DCO‐rate (Death Certificate Only) | Excluded before SR: Covered by IDEFIM-1026 |
| TMF-1031 | Refusal rate of investigations | Final set: IDEFIM-1027 |
| TMF-1032 | Refusal rate of modules | Final set: IDEFIM-1028 |
| TMF-1033 | Refusal rate of single data elements | Final set: IDEFIM-1029 |
| TMF-1034 | Drop-out-rate | Final set: IDEFIM-1030 |
| TMF-1036 | Synonyms (data) | Final set: IDEFIM-1031 |
| TMF-1037 | Homonyms (data) | Final set: IDEFIM-1032 |
| TMF-1038 | Single notification per observational unit | No match in the SR |
| TMF-1039 | Sole notifications from pathologists | Excluded before SR: Covered by IDEFIM-1033 |
| TMF-1040 | Rejected notifications | No match in the SR |
| TMF-1041 | Single data source per observational unit | Final set: IDEFIM-1035 |
| TMF-1042 | Observational units with follow-up | Final set: IDEFIM-1036 |
| **Trueness** |  |  |
| TMF-1043 | Correctness | Final set: IDEFIM-1037 |
| TMF-1044 | Disagreement with source data referring to data elements | Final set: IDEFIM-1038 |
| TMF-1045 | Disagreement with source data referring to observational units | Final set: IDEFIM-1039 |
| TMF-1046 | Recall | Final set: IDEFIM-1040 |
| TMF-1047 | Incompliance with operating procedures | Category context quality |
| TMF-1048 | Representativeness | Final set: IDEFIM-1042 |

^#^Designations partly revised
^&^IDEFIM identifiers are solved in appendices B and C
QI=Quality indicator, SR=Scoping review
