# Supplementary material for: Measuring the Quality of Datasets: Development of the IDEFIM Indicator Set for Empirical Health Research
Source: J Med Internet Res. 2026 Jun 17;28:e90482. doi: 10.2196/90482 (PMC13274964; doi:10.2196/90482)
Supplement: Multimedia Appendix 2 [file jmir-v28-e90482-s002.docx]

# Search strings used for Medline, the Cochrane Library, the Web of Science, and Scopus

## Medline

“data quality” [title] OR (“quality control“ [MeSH Terms] AND “data quality” [title/abstract]) OR (“data collection“ [title] AND “data quality” [title/abstract]) OR “data accuracy“ [title] OR (“dimensional measurement accuracy“ [MeSH Terms] AND “data quality” [title/abstract]) OR (“research design“ [MeSH Major Topic] AND “data reporting“ [Other Term]) AND “2013/01/01“ [Date - Publication]: “3000“ [Date - Publication]

## Cochrane Library

The search string was “data quality” (with quotation marks) in “Title Abstract Keyword”.

## Web of Science

The search string was (TI=(“data quality”)) AND AB=(“data quality”) with restriction to the languages German and English and the years from 2013 to 2024.

## Scopus

SUBJAREA ( medi OR nurs ) AND TITLE-ABS ( “data quality” ) AND INDEXTERMS ( “data quality” ) AND PUBYEAR > 2012 AND PUBYEAR < 2025 AND ( LIMIT-TO ( EXACTKEYWORD , “Controlled Study” ) OR LIMIT-TO ( EXACTKEYWORD , “Major Clinical Study” ) OR LIMIT-TO ( EXACTKEYWORD , “Registries” ) OR LIMIT-TO ( EXACTKEYWORD , “Register” ) OR LIMIT-TO ( EXACTKEYWORD , “Epidemiology” ) OR LIMIT-TO ( EXACTKEYWORD , “Cohort Analysis” ) OR LIMIT-TO ( EXACTKEYWORD , “Retrospective Study” ) OR LIMIT-TO ( EXACTKEYWORD , “Clinical Research” ) OR LIMIT-TO ( EXACTKEYWORD , “Cross-sectional Study” ) OR LIMIT-TO ( EXACTKEYWORD , “Multicenter Study” ) OR LIMIT-TO ( EXACTKEYWORD , “Retrospective Studies” ) OR LIMIT-TO ( EXACTKEYWORD , “Research Design” ) OR LIMIT-TO ( EXACTKEYWORD , “Medical Research” ) OR LIMIT-TO ( EXACTKEYWORD , “Prospective Study” ) OR LIMIT-TO ( EXACTKEYWORD , “Clinical Trial” ) OR LIMIT-TO ( EXACTKEYWORD , “Randomized Controlled Trial (topic)” ) OR LIMIT-TO ( EXACTKEYWORD , “Comparative Study” ) OR LIMIT-TO ( EXACTKEYWORD , “Feasibility Study” ) OR LIMIT-TO ( EXACTKEYWORD , “Quantitative Analysis” ) OR LIMIT-TO ( EXACTKEYWORD , “Observational Study” ) OR LIMIT-TO ( EXACTKEYWORD , “Cross-Sectional Studies” ) OR LIMIT-TO ( EXACTKEYWORD , “Randomized Controlled Trial” ) OR LIMIT-TO ( EXACTKEYWORD , “Pilot Study” ) OR LIMIT-TO ( EXACTKEYWORD , “Clinical Trial (topic)” ) OR LIMIT-TO ( EXACTKEYWORD , “Longitudinal Study” ) ) AND ( LIMIT-TO ( LANGUAGE , “English” ) OR LIMIT-TO ( LANGUAGE , “German” ) )
