# Supplementary figures and images for: Measuring the Quality of Datasets: Development of the IDEFIM Indicator Set for Empirical Health Research
Source: J Med Internet Res. 2026 Jun 17;28:e90482. doi: 10.2196/90482 (PMC13274964; doi:10.2196/90482)

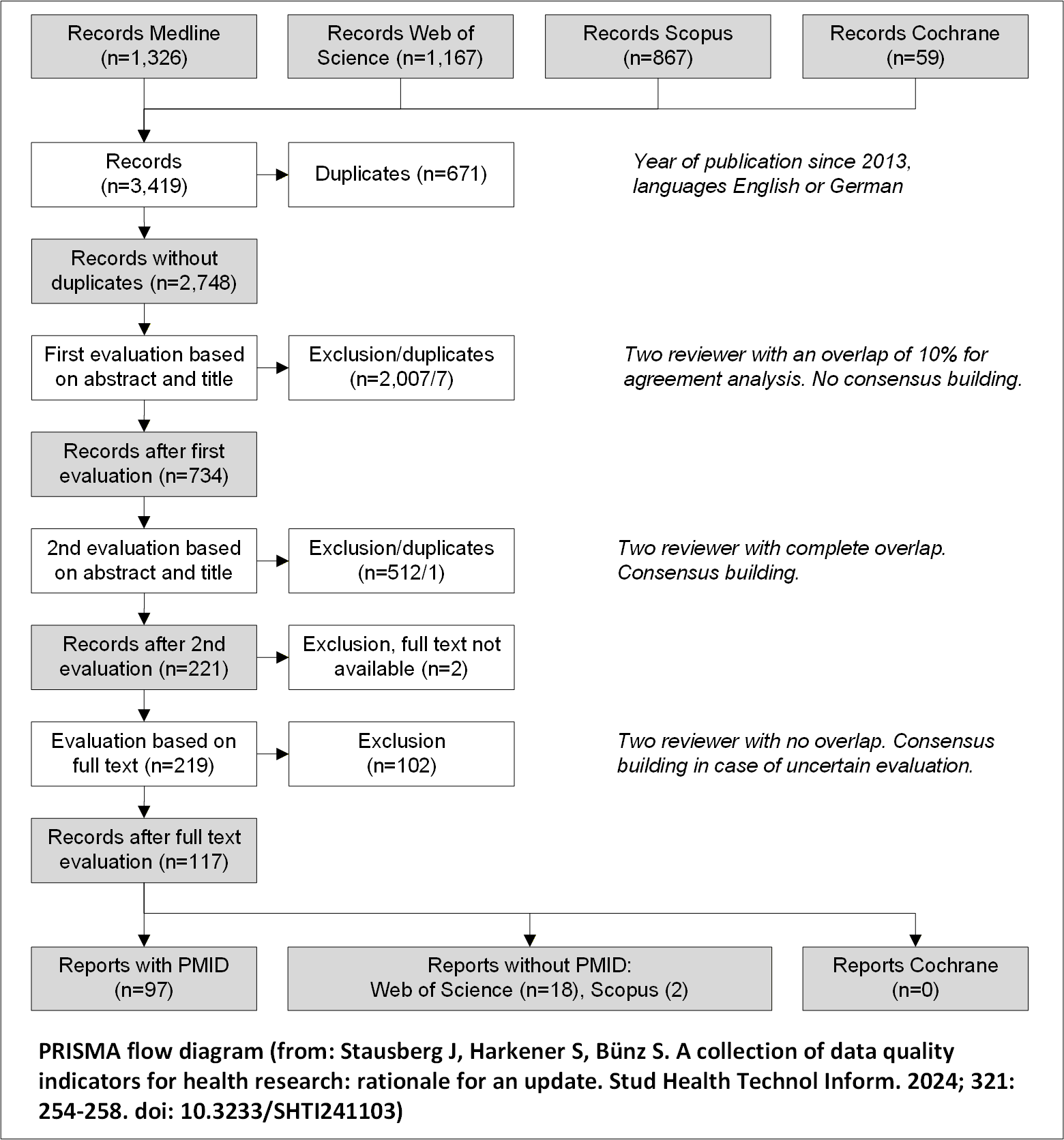

Supplement: Multimedia Appendix 3 [file jmir-v28-e90482-s003.png]
