# Supplementary material for: Measuring the Quality of Datasets: Development of the IDEFIM Indicator Set for Empirical Health Research
Source: J Med Internet Res. 2026 Jun 17;28:e90482. doi: 10.2196/90482 (PMC13274964; doi:10.2196/90482)
Supplement: Multimedia Appendix 4 [file jmir-v28-e90482-s004.docx]

Evidence Sources per Indicator in Category Data Quality

| **Dimension/indicator group** | **ID** | **Evidence source** |
| --- | --- | --- |
| **Accuracy (data)** |  |  |
| Contingency table indicators |  |  |
| Conspicuous correctness distribution | IDEFIM-1075 |  |
| Correctness | IDEFIM-1037 | Alhaug et al. 2022, Arboe et al. 2016, Azeroual et al. 2018, Couchoud et al. 2013, Ehrlinger et al. 2022, García-De-León-Chocano et al. 2015, Gualo et al. 2021, Heinänen et al. 2021, Kalincik et al. 2017, Laberge et al. 2013, Liaw et al. 2013, Lim et al. 2018, Orlandi et al. 2020, Porgo et al. 2016, Quindroit et al. 2023, Schmidt et al. 2021, Terry et al. 2019, Wolf et al. 2023 |
| Recall | IDEFIM-1040 | Stausberg et al. 2022, Stausberg et al. 2023, Tahar et al. 2023, Terry et al. 2019, Tosti et al. 2015, Wolf et al. 2023 |
| Validity | IDEFIM-1079 |  |
| Disagreement with source data indicators |  |  |
| Disagreement with source data referring to data elements | IDEFIM-1038 | Alhaug et al. 2022, Arboe et al. 2016, Argaw et al. 2022, Bashar et al. 2021, Bian et al. 2020, Couchoud et al. 2013, Dunn et al. 2019, Elouataoui et al. 2022, Gisslander et al. 2023, Heinänen et al. 2021, Landberg et al. 2021, Lasim et al. 2022, Lee et al. 2020, Lim et al. 2018, Liu et al. 2023, Löfgren et al. 2019, Rao et al. 2016, Saucedo et al. 2023, Seagrave et al. 2014, Tan et al. 2019, Tomic et al. 2015 |
| Disagreement with source data referring to observational units | IDEFIM-1039 | Gass et al. 2017 |
| Illegal content indicators |  |  |
| Illegal values of qualitative data elements | IDEFIM-1017 | Aerts et al. 2021, Blacketer et al. 2021, Brown et al. 2013, Groisman et al. 2019, Huser et al. 2019, Khare et al. 2017, Lee et al. 2018, Schmidt et al. 2021, Shaheen et al. 2019, Wolf et al. 2023, Zhang et al. 2020 |
| Illegal values of qualitative data elements used for the coding of missings | IDEFIM-1018 | Liu et al. 2023, Quindroit et al. 2023, Schmidt et al. 2021, Zhang et al. 2020 |
| Incorrect text in qualitative data elements | IDEFIM-1051 | Elouataoui et al. 2022, Gualo et al. 2021, Quindroit et al. 2023, Zhang et al. 2020 |
| Misfielded values | IDEFIM-1081 | Khare et al. 2017, Quindroit et al. 2023, Zhang et al. 2020 |
| Other indicators for accuracy (data) |  |  |
| Granularity (data) | IDEFIM-1050 | Eder et al. 2021, Feder et al. 2018 |
| Temporal trends in counts or proportions | IDEFIM-1052 | Brown et al. 2013 |
| **Completeness (cases)** |  |  |
| Indicators for completeness (cases) |  |  |
| Conspicuous recruitment rate distribution | IDEFIM-1076 |  |
| Drop-out-rate | IDEFIM-1030 | Allemani et al. 2017, Heinänen et al. 2021, Khare et al. 2017, Lee et al. 2020, Schmidt et al. 2021, Stausberg et al. 2023 |
| Recruitment rate | IDEFIM-1026 | Anderka et al. 2015, Arboe et al. 2016, Blacketer et al. 2021, Couchoud et al. 2013, D'Ambrosio et al. 2021, Heinänen et al. 2021, Laberge et al. 2013, Landberg et al. 2021, Lee et al. 2020, Löfgren et al. 2019, Schmidt et al. 2021, Seagrave et al. 2014, Stausberg et al. 2023, Tan et al. 2019, Tomic et al. 2015 |
| **Completeness (data)** |  |  |
| Missing content indicators |  |  |
| Conspicuous missing values distribution | IDEFIM-1078 |  |
| Missing modules | IDEFIM-1009 | Argaw et al. 2022, Laberge et al. 2013, Lasim et al. 2022, Schmidt et al. 2021, Tosti et al. 2015 |
| Missing values in data elements | IDEFIM-1010 | Aerts et al. 2021, Alhaug et al. 2022, Arboe et al. 2016, Bian et al. 2020, Blacketer et al. 2021, Boes et al. 2020, Brown et al. 2013, Chen et al. 2018, Corrêa et al. 2023, Couchoud et al. 2013, D'Ambrosio et al. 2021, Eder et al. 2021, Ehrlinger et al. 2022, Elouataoui et al. 2022, Feder et al. 2018, Gisslander et al. 2023, Glöckner et al. 2017, Groisman et al. 2019, Gualo et al. 2021, Heinänen et al. 2021, Kahn et al. 2018, Kalincik et al. 2017, Khare et al. 2017, Laberge et al. 2013, Lee et al. 2018, Liaw et al. 2013, Lim et al. 2018, Liu et al. 2023, Mashoufi et al. 2023, Odeny et al. 2023, Orlandi et al. 2020, Porgo et al. 2016, Quindroit et al. 2023, Rau et al. 2022, Saucedo et al. 2023, Schmidt et al. 2021, Seagrave et al. 2014, Shaheen et al. 2019, Silva et al. 2017, Skyttberg et al. 2017, Tahar et al. 2023, Tan et al. 2019, Terry et al. 2019, Tlale et al. 2019, Tosti et al. 2015, Tute et al. 2023, Wiley et al. 2022, Woods et al. 2021 |
| Temporal missingness | IDEFIM-1080 |  |
| Other indicators for completeness (data) |  |  |
| Data elements with existing entries for all observational units | IDEFIM-1014 | Lee et al. 2020, Terry et al. 2019 |
| Information density score | IDEFIM-1056 | Wiley et al. 2022 |
| Modules with existing entries for all data elements | IDEFIM-1045 | Tahar et al. 2023 |
| Observational units with existing entries for all data elements | IDEFIM-1049 | García-de-León-Chocano et al. 2015, Gualo et al. 2021, Perren et al. 2019, Tahar et al. 2023 |
| Observational units with follow-up | IDEFIM-1036 | Stausberg et al. 2023 |
| Refusal rate indicators |  |  |
| Refusal rate of investigations | IDEFIM-1027 | Schmidt et al. 2021 |
| Refusal rate of modules | IDEFIM-1028 | Schmidt et al. 2021 |
| Refusal rate of single data elements | IDEFIM-1029 | Draisma et al. 2015 |
| **Compliance (data)** |  |  |
| Indicators for compliance (data) |  |  |
| Data format, data type, and unit compliance | IDEFIM-1057 | Aerts et al. 2021, Eder et al. 2021, Elouataoui et al. 2022, Gisslander et al. 2023, Gualo et al. 2021, Kahn et al. 2018, Lee et al. 2018, Oh et al. 2023, Quindroit et al. 2023, Schmidt et al. 2021, Shaheen et al. 2019, Skyttberg et al. 2017, Zhang et al. 2020 |
| Incompliance with metadata | IDEFIM-1058 | Brown et al. 2013 |
| Range compliance | IDEFIM-1043 | Blacketer et al. 2021, García-de-León-Chocano et al. 2015, Gualo et al. 2021, Kalincik et al. 2017, Oh et al. 2023, Schmidt et al. 2021, Tute et al. 2023, Zhang et al. 2020 |
| **Consistency (data)** |  |  |
| Confusion and redundancy indicators |  |  |
| Confusion | IDEFIM-1083 |  |
| Duplicates (data) | IDEFIM-1025 | Aerts et al. 2021, Bian et al. 2020, Blacketer et al. 2021, Elouataoui et al. 2022, García-De-León-Chocano et al. 2015, Gualo et al. 2021, Kahn et al. 2018, Liu et al. 2023, Oh et al. 2023, Quindroit et al. 2023, Schmidt et al. 2021, Woods et al. 2021, Zhang et al. 2020 |
| Homonyms (data) | IDEFIM-1032 | Aerts et al. 2021, Quindroit et al. 2023 |
| Synonyms (data) | IDEFIM-1031 | Couchoud et al. 2013, D'Ambrosio et al. 2021, Gisslander et al. 2023, Lee et al. 2018, Quindroit et al. 2023, Tahar et al. 2023, Woods et al. 2021, Zhang et al. 2020 |
| Contradiction indicators |  |  |
| Contradictions | IDEFIM-1003 | Aerts et al. 2021, Allemani et al. 2017, Ayele et al. 2023, Azeroual et al. 2018, Bian et al. 2020, Blacketer et al. 2021, Brown et al. 2013, D'Ambrosio et al. 2021, Draisma et al. 2015, Eder et al. 2021, Ehrlinger et al. 2022, Gisslander et al. 2023, Gualo et al. 2021, Kahn et al. 2018, Kalincik et al. 2017, Khare et al. 2017, Laberge et al. 2013, Lasim et al. 2022, Lee et al. 2018, Liu et al. 2023, Odeny et al. 2023, Oh et al. 2023, Perren et al. 2019, Quindroit et al. 2023, Salati et al. 2016, Saucedo et al. 2023, Silva et al. 2017, Stausberg et al. 2023, Tahar et al. 2023, Woods et al. 2021, Zhang et al. 2020 |
| Data element contradictions | IDEFIM-1048 | Corrêa et al. 2023, Perren et al. 2019 |
| Other indicators for consistency (data) |  |  |
| Missing evidence of known correlations | IDEFIM-1022 | Draisma et al. 2015, Groisman et al. 2019, Odeny et al. 2023, Schmidt et al. 2021 |
| Single data source per observational unit | IDEFIM-1035 | Bashar et al. 2021, Wu et al. 2022 |
| Temporality of categorical data elements | IDEFIM-1060 | García-de-León-Chocano et al. 2015 |
| Unexpected entry indicators |  |  |
| Concordance | IDEFIM-1002 | Ayele et al. 2023, Bashar et al. 2021, Bian et al. 2020, Feder et al. 2018, Heinänen et al. 2021, Lee et al. 2020, Lim et al. 2018, Liu et al. 2023, Mashoufi et al. 2023, Quindroit et al. 2023, Schmidt et al. 2021, Skyttberg et al. 2017, Terry et al. 2019, Tlale et al. 2019 |
| Conspicuous distribution of digits in date-time data elements | IDEFIM-1059 | Allemani et al. 2017 |
| Conspicuous distribution of values | IDEFIM-1006 | Brown et al. 2013, Corrêa et al. 2023, Khare et al. 2017, Porgo et al. 2016, Schmidt et al. 2021, Skyttberg et al. 2017 |
| Data elements with value unknown etc. | IDEFIM-1013 | Bashar et al. 2021, Glöckner et al. 2017, Schmidt et al. 2021 |
| Disagreement with previous values | IDEFIM-1001 | Aerts et al. 2021, Lasim et al. 2022, Rau et al. 2022 |
| Frequency outliers | IDEFIM-1046 | Khare et al. 2017 |
| Last digit preferences | IDEFIM-1007 | Allemani et al. 2017, Corrêa et al. 2023 |
| Outliers (continuous data elements) | IDEFIM-1015 | Aerts et al. 2021, Ayele et al. 2023, Brown et al. 2013, D'Ambrosio et al. 2021, Gisslander et al. 2023, Kahn et al. 2018, Khare et al. 2017, Lasim et al. 2022, Lee et al. 2020, Liu et al. 2023, Quindroit et al. 2023, Rau et al. 2022, Schmidt et al. 2021, Shaheen et al. 2019, Skyttberg et al. 2017, Stausberg et al. 2023, Tahar et al. 2023 |
| Outliers in numerical data elements in a multivariate analysis | IDEFIM-1061 | Schmidt et al. 2021 |
| Values from external references | IDEFIM-1016 | Khare et al. 2017, Liaw et al. 2013, Liu et al. 2023 |
| **Credibility** |  |  |
| Indicators for credibility |  |  |
| Data element credibility | IDEFIM-1044 | Gualo et al. 2021, Wu et al. 2022 |
| **Currentness** |  |  |
| Indicators for currentness |  |  |
| Currentness | IDEFIM-1024 | Anderka et al. 2015, Argaw et al. 2022, Azeroual et al. 2018, Boes et al. 2020, Couchoud et al. 2013, Dimitrova et al. 2015, Ehrlinger et al. 2022, Elouataoui et al. 2022, Feder et al. 2018, Gualo et al. 2021, Laberge et al. 2013, Landberg et al. 2021, Lasim et al. 2022, Liaw et al. 2013, Lim et al. 2018, Liu et al. 2023, Löfgren et al. 2019, Mashoufi et al. 2023, Rao et al. 2016, Saucedo et al. 2023, Silva et al. 2017, Terry et al. 2019, Tomic et al. 2015, Tosti et al. 2015, Wiley et al. 2022, Wu et al. 2022 |
| **Representativeness** |  |  |
| Indicators for representativeness |  |  |
| Conspicuous representativeness distribution | IDEFIM-1077 |  |
| Representativeness | IDEFIM-1042 | Bashar et al. 2021, Dimitrova et al. 2015, Groisman et al. 2019, Liaw et al. 2013, Rau et al. 2022, Tahar et al. 2023, Terry et al. 2019 |

**References**

Aerts, H, Kalra, D, Sáez, C, Ramírez-Anguita, JM, Mayer, MA, Garcia-Gomez, JM, Durà-Hernández, M, Thienpont, G & Coorevits, P (2021) Quality of Hospital Electronic Health Record (EHR) Data Based on the International Consortium for Health Outcomes Measurement (ICHOM) in Heart Failure: Pilot Data Quality Assessment Study. *JMIR Med Inform* 9:e27842. DOI: 10.2196/27842.

Alhaug, OK, Kaur, S, Dolatowski, F, Smastuen, MC, Solberg, TK & Lonne, G (2022) Accuracy and agreement of national spine register data for 474 patients compared to corresponding electronic patient records. *Eur Spine J* 31:801-811. DOI: 10.1007/s00586-021-07093-8.

Allemani, C, Harewood, R, Johnson, CJ, Carreira, H, Spika, D, Bonaventure, A, Ward, K, Weir, HK & Coleman, MP (2017) Population-based cancer survival in the United States: Data, quality control, and statistical methods. *Cancer* 123 Suppl 24:4982-4993. DOI: 10.1002/cncr.31025.

Anderka, M, Mai, CT, Romitti, PA, Copeland, G, Isenburg, J, Feldkamp, ML, Krikov, S, Rickard, R, Olney, RS, Canfield, MA, Stanton, C, Mosley, B & Kirby, RS (2015) Development and implementation of the first national data quality standards for population-based birth defects surveillance programs in the United States. *BMC Public Health* 15:925. DOI: 10.1186/s12889-015-2223-2.

Arboe, B, El-Galaly, TC, Clausen, MR, Munksgaard, PS, Stoltenberg, D, Nygaard, MK, Klausen, TW, Christensen, JH, Gørløv, JS & Brown Pde, N (2016) The Danish National Lymphoma Registry: Coverage and Data Quality. *PLoS One* 11:e0157999. DOI: 10.1371/journal.pone.0157999.

Argaw, MD, Desta, BF, Tsegaye, ZT, Mitiku, AD, Atsa, AA, Tefera, BB, Rogers, D, Teferi, E, Abera, WS, Beshir, IA, Kora, ZA, Setegn, S, Anara, AA, Sinamo, T & Muloiwa, R (2022) Immunization data quality and decision making in pertussis outbreak management in southern Ethiopia: a cross sectional study. *Arch Public Health* 80:49. DOI: 10.1186/s13690-022-00805-6.

Ayele W, Gage A, Kapoor NR, Kassahun Gelaw S, Hensman D, Derseh Mebratie A, Nega A, Asai D, Molla G, Mehata S, Mthethwa L, Mfeka-Nkabinde NG, Joseph JP, Pierre DM, Thermidor R & Arsenault C (2023) Quality of routine health data at the onset of the COVID-19 pandemic in Ethiopia, Haiti, Laos, Nepal, and South Africa. *Popul Health Metr* 20;21(1):7. DOI: 10.1186/s12963-023-00306-w.

Azeroual, O, Saake, G & Wastl, J (2018) Data measurement in research information systems: metrics for the evaluation of data quality. *Scientometrics* 115:1271-1290. DOI: 10.1007/s11192-018-2735-5.

Bashar, MA, Thakur, JS & Budukh, A (2021) Evaluation of Data Quality of Four New Population Based Cancer Registries (PBCRs) in Chandigarh and Punjab, North India - A Quality Control Study. *Asian Pac J Cancer Prev* 22:1421-1433. DOI: 10.31557/apjcp.2021.22.5.1421.

Bian, J, Lyu, T, Loiacono, A, Viramontes, TM, Lipori, G, Guo, Y, Wu, Y, Prosperi, M, George, TJ, Harle, CA, Shenkman, EA & Hogan, W (2020) Assessing the practice of data quality evaluation in a national clinical data research network through a systematic scoping review in the era of real-world data. *J Am Med Inform Assoc* 27:1999-2010. DOI: 10.1093/jamia/ocaa245.

Blacketer, C, Defalco, FJ, Ryan, PB & Rijnbeek, PR (2021) Increasing trust in real-world evidence through evaluation of observational data quality. *J Am Med Inform Assoc* 28:2251-2257. DOI: 10.1093/jamia/ocab132.

Boes, L, Houareau, C, Altmann, D, An der Heiden, M, Bremer, V, Diercke, M, Dudareva, S, Neumeyer-Gromen, A & Zimmermann, R (2020) Evaluation of the German surveillance system for hepatitis B regarding timeliness, data quality, and simplicity, from 2005 to 2014. *Public Health* 180:141-148. DOI: 10.1016/j.puhe.2019.11.012.

Brown, JS, Kahn, M & Toh, S (2013) Data quality assessment for comparative effectiveness research in distributed data networks. *Med Care* 51:S22-9. DOI: 10.1097/MLR.0b013e31829b1e2c.

Chen, Y, Lin, HY, Tseng, TS, Wen, H & DeVivo, MJ (2018) Racial Differences in Data Quality and Completeness: Spinal Cord Injury Model Systems' Experiences. *Top Spinal Cord Inj Rehabil* 24:110-120. DOI: 10.1310/sci2402-110.

Correa, FF, Carrilho, TRB, Bonilha, EA, Keller, VN, Melo, TC, Kac, G & Diniz, CSG (2023) Analysis of the quality of prenatal data of pregnant women attended at Healthcare Services in the city of Sao Paulo between 2012 and 2020. *Rev Bras Epidemiol* 26:e230051. DOI: 10.1590/1980-549720230051.

Couchoud, C, Lassalle, M, Cornet, R & Jager, KJ (2013) Renal replacement therapy registries - time for a structured data quality evaluation programme. *Nephrol Dial Transplant* 28:2215-20. DOI: 10.1093/ndt/gft004.

D'Ambrosio, A, Garlasco, J, Quattrocolo, F, Vicentini, C & Zotti, CM (2021) Data quality assessment and subsampling strategies to correct distributional bias in prevalence studies. *BMC Med Res Methodol* 21:90. DOI: 10.1186/s12874-021-01277-y.

Dimitrova, N & Parkin, DM (2015) Data quality at the Bulgarian National Cancer Registry: An overview of comparability, completeness, validity and timeliness. *Cancer Epidemiol* 39:405-13. DOI: 10.1016/j.canep.2015.03.015.

Draisma, S, van Zaane, J & Smit, JH (2015) Data quality indicators for daily life chart methodology: prospective self-ratings of bipolar disorder and alcohol use. *BMC Res Notes* 8:473. DOI: 10.1186/s13104-015-1436-x.

Dunn, S, Lanes, A, Sprague, AE, Fell, DB, Weiss, D, Reszel, J, Taljaard, M, Darling, EK, Graham, ID, Grimshaw, JM, Harrold, J, Smith, GN, Peterson, W & Walker, M (2019) Data accuracy in the Ontario birth Registry: a chart re-abstraction study. *BMC Health Serv Res* 19:1001. DOI: 10.1186/s12913-019-4825-3.

Eder J & Shekhovtsov VA (2021) Data quality for federated medical data lakes. *Int J Web Inf Syst* Vol. 17(5):407-426. DOI: 10.1108/IJWIS-03-2021-0026.

Ehrlinger, L & Wöß, W (2022) A Survey of Data Quality Measurement and Monitoring Tools. *Front Big Data* 5:850611. DOI: 10.3389/fdata.2022.850611.

Elouataoui, W, El Alaoui, I, El Mendili, S, & Gahi, Y (2022) An Advanced Big Data Quality Framework Based on Weighted Metrics. *Big data cogn comput* 6(4):153. DOI: 10.3390/bdcc6040153.

Feder, SL (2018) Data Quality in Electronic Health Records Research: Quality Domains and Assessment Methods. *West J Nurs Res* 40:753-766. DOI: 10.1177/0193945916689084.

García-de-León-Chocano, R, Sáez, C, Muñoz-Soler, V, García-de-León-González, R & García-Gómez, JM (2015) Construction of quality-assured infant feeding process of care data repositories: definition and design (Part 1). *Comput Biol Med* 67:95-103. DOI: 10.1016/j.compbiomed.2015.09.024.

Gass, JD, Jr., Misra, A, Yadav, MNS, Sana, F, Singh, C, Mankar, A, Neal, BJ, Fisher-Bowman, J, Maisonneuve, J, Delaney, MM, Kumar, K, Singh, VP, Sharma, N, Gawande, A, Semrau, K & Hirschhorn, LR (2017) Implementation and results of an integrated data quality assurance protocol in a randomized controlled trial in Uttar Pradesh, India. *Trials* 18:418. DOI: 10.1186/s13063-017-2159-1.

Gisslander, K, Rutherford, M, Aslett, L, Basu, N, Dradin, F, Hederman, L, Hruskova, Z, Kardaoui, H, Lamprecht, P, Lichołai, S, Musial, J, O'Sullivan, D, Puechal, X, Scott, J, Segelmark, M, Straka, R, Terrier, B, Tesar, V, Tesi, M, Vaglio, A, Wandrei, D, White, A, Wójcik, K, Yaman, B, Little, MA & Mohammad, AJ (2024) Data quality and patient characteristics in European ANCA-associated vasculitis registries: data retrieval by federated querying. *Ann Rheum Dis* 83:112-120. DOI: 10.1136/ard-2023-224571.

Glöckner, S, Toikkanen, SE, Rui, Hu, JW & Sinnott, R (2017) A Case Study of the International Niemann-Pick Disease Registry (INPDR). *IEEE 19th International Conference on e-Health Networking, Applications and Services (Healthcom)*, Dalian, China, 2017, pp. 1-6. DOI: 10.1109/HealthCom.2017.8210764.

Groisman, B, Mastroiacovo, P, Barbero, P, Bidondo, MP, Liascovich, R & Botto, LD (2019) A proposal for the systematic assessment of data quality indicators in birth defects surveillance. *Birth Defects Res* 111:324-332. DOI: 10.1002/bdr2.1474.

Gualo, F, Rodriguez, M, Verdugo, J, Caballero, I & Piattini, M (2021) Data quality certification using ISO/IEC 25012: Industrial experiences. *J Syst Softw* 176:110938. DOI: 10.1016/j.jss.2021.110938.

Heinänen, M, Brinck, T, Lefering, R, Handolin, L & Söderlund, T (2021) How to Validate Data Quality in a Trauma Registry? The Helsinki Trauma Registry Internal Audit. *Scand J Surg* 110:199-207. DOI: 10.1177/1457496919883961.

Huser, V, Li, X, Zhang, Z, Jung, S, Park, RW, Banda, J, Razzaghi, H, Londhe, A & Natarajan, K (2019) Extending Achilles Heel Data Quality Tool with New Rules Informed by Multi-Site Data Quality Comparison. *Stud Health Technol Inform* 264:1488-1489. DOI: 10.3233/shti190498.

Kahn, M, Ong, T, Barnard, J & Maertens, J (2018) PCORI Final Research Reports, *Developing Standards for Improving Measurement and Reporting of Data Quality in Health Research*, Washington (DC), Patient-Centered Outcomes Research Institute (PCORI). DOI: 10.25302/3.2018.ME.13035581.

Kalincik, T, Kuhle, J, Pucci, E, Rojas, JI, Tsolaki, M, Sirbu, CA, Slee, M & Butzkueven, H (2017) Data quality evaluation for observational multiple sclerosis registries. *Mult Scler* 23:647-655. DOI: 10.1177/1352458516662728.

Khare, R, Utidjian, L, Ruth, BJ, Kahn, MG, Burrows, E, Marsolo, K, Patibandla, N, Razzaghi, H, Colvin, R, Ranade, D, Kitzmiller, M, Eckrich, D & Bailey, LC (2017) A longitudinal analysis of data quality in a large pediatric data research network. *J Am Med Inform Assoc* 24:1072-1079. DOI: 10.1093/jamia/ocx033.

Laberge, M & Shachak, A (2013) Developing a tool to assess the quality of socio-demographic data in community health centres. *Appl Clin Inform* 4:1-11. DOI: 10.4338/aci-2012-10-cr-0041.

Landberg, A, Bruce, D, Lindblad, P, Ljungberg, B, Lundstam, S, Thorstenson, A & Sundqvist, P (2021) Validation of data quality in the National Swedish Kidney Cancer Register. *Scand J Urol* 55:142-148. DOI: 10.1080/21681805.2021.1885485.

Lasim, OU, Ansah, EW & Apaak, D (2022) Maternal and child health data quality in health care facilities at the Cape Coast Metropolis, Ghana. *BMC Health Serv Res* 22:1102. DOI: 10.1186/s12913-022-08449-6.

Lee, K, Weiskopf, N & Pathak, J (2017) A Framework for Data Quality Assessment in Clinical Research Datasets. *AMIA Annu Symp Proc* 2017:1080-1089.

Lee, B, Ebrahimi, M, Ektas, N, Ting, CH, Cowley, M, Scholes, C & Bell, C (2020) Implementation and quality assessment of a clinical orthopaedic registry in a public hospital department. *BMC Health Serv Res* 20:393. DOI: 10.1186/s12913-020-05203-8.

Liaw, ST, Rahimi, A, Ray, P, Taggart, J, Dennis, S, de Lusignan, S, Jalaludin, B, Yeo, AE & Talaei-Khoei, A (2013) Towards an ontology for data quality in integrated chronic disease management: a realist review of the literature. *Int J Med Inform* 82:10-24. DOI: 10.1016/j.ijmedinf.2012.10.001.

Lim, YMF, Yusof, M & Sivasampu, S (2018) Assessing primary care data quality. *Int J Health Care Qual Assur* 31:203-213. DOI: 10.1108/ijhcqa-08-2016-0111.

Liu, C, Talaei-Khoei, A, Storey, VC & Peng, G (2023) A Review of the State of the Art of Data Quality in Healthcare. *J Glob Inf Manag* 31(1):1-18. DOI: 10.4018/JGIM.316236.

Löfgren, L, Eloranta, S, Krawiec, K, Asterkvist, A, Lönnqvist, C & Sandelin, K (2019) Validation of data quality in the Swedish National Register for Breast Cancer. *BMC Public Health* 19:495. DOI: 10.1186/s12889-019-6846-6.

Mashoufi, M, Ayatollahi, H, Khorasani-Zavareh, D & Talebi Azad Boni, T (2023) Data quality assessment in emergency medical services: an objective approach. *BMC Emerg Med* 23:10. DOI: 10.1186/s12873-023-00781-2.

Odeny, BM, Njoroge, A, Gloyd, S, Hughes, JP, Wagenaar, BH, Odhiambo, J, Nyagah, LM, Manya, A, Oghera, OW & Puttkammer, N (2023) Development of novel composite data quality scores to evaluate facility-level data quality in electronic data in Kenya: a nationwide retrospective cohort study. *BMC Health Serv Res* 23:1139. DOI: 10.1186/s12913-023-10133-2.

Oh, SW, Ko, SJ, Im, YS, Jung, S, Choi, BY, Kim, JY, Choi, W & Choi, IY (2023) Data Quality Assessment for Observational Medical Outcomes Partnership Common Data Model of Multi-Center. *Stud Health Technol Inform* 302:322-326. DOI: 10.3233/shti230127.

Orlandi, BMM, Mejia, OAV, Borgomoni, GB, Goncharov, M, Rocha, KN, Bassolli, L, Melo de Barros, ESPG, Nakazone, MA, Sousa, A, Campagnucci, VP, de Sousa Vilarinho, KA, Katz, M, Tiveron, MG, Arrais Dos Santos, M, Lisboa, LAF, Dallan, LAO & Jatene, FB (2020) REPLICCAR II Study: Data quality audit in the Paulista Cardiovascular Surgery Registry. *PLoS One* 15:e0223343. DOI: 10.1371/journal.pone.0223343.

Perren, A, Cerutti, B, Kaufmann, M & Rothen, HU (2019) A novel method to assess data quality in large medical registries and databases. *Int J Qual Health Care* 31:1-7. DOI: 10.1093/intqhc/mzy249.

Porgo, TV, Moore, L & Tardif, PA (2016) Evidence of data quality in trauma registries: A systematic review. *J Trauma Acute Care Surg* 80:648-58. DOI: 10.1097/ta.0000000000000970.

Quindroit, P, Fruchart, M, Degoul, S, Perichon, R, Martignène, N, Soula, J, Marcilly, R & Lamer, A (2023) Definition of a Practical Taxonomy for Referencing Data Quality Problems in Health Care Databases. *Methods Inf Med* 62:19-30. DOI: 10.1055/a-1976-2371.

Rao, C, Zhang, H, Gao, H, Zhao, Y, Yuan, X, Hua, K, Hu, S & Zheng, Z (2016) The Chinese Cardiac Surgery Registry: Design and Data Audit. *Ann Thorac Surg* 101:1514-20. DOI: 10.1016/j.athoracsur.2015.09.038.

Rau, C, Lüdecke, D, Dumolard, LB, Grevendonk, J, Wiernik, BM, Kobbe, R, Gacic-Dobo, M & Danovaro-Holliday, MC (2022) Data quality of reported child immunization coverage in 194 countries between 2000 and 2019. *PLOS Glob Public Health* 2:e0000140. DOI: 10.1371/journal.pgph.0000140.

Salati, M, Falcoz, PE, Decaluwe, H, Rocco, G, Van Raemdonck, D, Varela, G & Brunelli, A (2016) The European thoracic data quality project: An Aggregate Data Quality score to measure the quality of international multi-institutional databases. *Eur J Cardiothorac Surg* 49:1470-5. DOI: 10.1093/ejcts/ezv385.

Saucedo, SCM, Silva, KR, Silva, LA, Crivelari, JM & Costa, R (2023) The impact of data quality monitoring of a multicenter prospective registry of cardiac implantable electronic devices. *MethodsX* 11:102454. DOI: 10.1016/j.mex.2023.102454.

Schmidt, CO, Struckmann, S, Enzenbach, C, Reineke, A, Stausberg, J, Damerow, S, Huebner, M, Schmidt, B, Sauerbrei, W & Richter, A (2021) Facilitating harmonized data quality assessments. A data quality framework for observational health research data collections with software implementations in R. *BMC Med Res Methodol* 21:63. DOI: 10.1186/s12874-021-01252-7.

Seagrave, KG, Naylor, J, Armstrong, E, Leong, KM, Descallar, J & Harris, IA (2014) Data quality audit of the arthroplasty clinical outcomes registry NSW. *BMC Health Serv Res* 14:512. DOI: 10.1186/s12913-014-0512-6.

Shaheen, NA, Manezhi, B, Thomas, A & AlKelya, M (2019) Reducing defects in the datasets of clinical research studies: conformance with data quality metrics. *BMC Med Res Methodol* 19:98. DOI: 10.1186/s12874-019-0735-7.

Silva, G, Bartholomay, P, Cruz, OG & Garcia, LP (2017) Evaluation of data quality, timeliness and acceptability of the tuberculosis surveillance system in Brazil's micro-regions. *Cien Saude Colet* 22:3307-3319. DOI: 10.1590/1413-812320172210.18032017.

Skyttberg, N, Chen, R, Blomqvist, H & Koch, S (2017) Exploring Vital Sign Data Quality in Electronic Health Records with Focus on Emergency Care Warning Scores. *Appl Clin Inform* 8:880-892. DOI: 10.4338/aci-2017-05-ra-0075.

Stausberg, J, Harkener, S, Jenetzky, E, Jersch, P, Martin, D, Rupp, R & Schonthaler, M (2022) FAIR and Quality Assured Data - The Use Case of Trueness. *Stud Health Technol Inform* 289:25-28. DOI: 10.3233/SHTI210850.

Stausberg, J, Harkener, S, Engel, C, Finger, R, Heinz, C, Jenetzky, E, Jersch, P, Martin, D, Rupp, R, Schoenthaler, M, Suwelack, B & Wegner, J (2023) Cross-Registry Benchmarking of Data Quality: Lessons Learned. *Stud Health Technol Inform* 302:167-171. DOI: 10.3233/shti230096.

Tahar, K, Martin, T, Mou, Y, Verbuecheln, R, Graessner, H & Krefting, D (2023) Rare Diseases in Hospital Information Systems - An Interoperable Methodology for Distributed Data Quality Assessments. *Methods Inf Med* 62:71-89. DOI: 10.1055/a-2006-1018.

Tan, AC, Armstrong, E, Close, J & Harris, IA (2019) Data quality audit of a clinical quality registry: a generic framework and case study of the Australian and New Zealand Hip Fracture Registry. *BMJ Open Qual* 8:e000490. DOI: 10.1136/bmjoq-2018-000490.

Terry, AL, Stewart, M, Cejic, S, Marshall, JN, de Lusignan, S, Chesworth, BM, Chevendra, V, Maddocks, H, Shadd, J, Burge, F & Thind, A (2019) A basic model for assessing primary health care electronic medical record data quality. *BMC Med Inform Decis Mak* 19:30. DOI: 10.1186/s12911-019-0740-0.

Tlale, LB, Morake, B, Lesetedi, O, Maribe, L, Masweu, M, Faye, C & Asiki, G (2019) Data quality self-assessment of child health and sexual reproductive health indicators in Botswana, 2016-2017. *PLoS One* 14:e0220313. DOI: 10.1371/journal.pone.0220313.

Tomic, K, Sandin, F, Wigertz, A, Robinson, D, Lambe, M & Stattin, P (2015) Evaluation of data quality in the National Prostate Cancer Register of Sweden. *Eur J Cancer* 51:101-11. DOI: 10.1016/j.ejca.2014.10.025.

Tosti, ME, Longhi, S, de Waure, C, Mele, A, Franco, E, Ricciardi, W & Filia, A (2015) Assessment of timeliness, representativeness and quality of data reported to Italy's national integrated surveillance system for acute viral hepatitis (SEIEVA). *Public Health* 129:561-8. DOI: 10.1016/j.puhe.2015.02.015.

Tute, E, Mast, M & Wulff, A (2023) Targeted Data Quality Analysis for a Clinical Decision Support System for SIRS Detection in Critically Ill Pediatric Patients. *Methods Inf Med* 62:e1-e9. DOI: 10.1055/s-0042-1760238.

Wiley, KK, Mendonca, E, Blackburn, J, Menachemi, N, Groot, M & Vest, JR (2022) Quantifying Electronic Health Record Data Quality in Telehealth and Office-Based Diabetes Care. *Appl Clin Inform* 13:1172-1180. DOI: 10.1055/s-0042-1758737.

Wolf, L, Usemann, J, Collaud, E, Derkenne, MF, Fischer, R, Hensen, M, Hitzler, M, Hofer, M, Inci, D, Irani, S, Jahn, K, Koutsokera, A, Kusche, R, Kurowski, T, Latzin, P, Lin, D, Mioranza, L, Moeller, A, Mornand, A, Mueller-Suter, D, Murer, C, Naehrlich, L, Plojoux, J, Regamey, N, Rodriguez, R, Rochat, I, Sauty, A, Schuurmans, M, Semmler, M, Trachsel, D, Walter, AL & Jung, A (2023) Data accuracy, consistency and completeness of the national Swiss cystic fibrosis patient registry: Lessons from an ECFSPR data quality project. *J Cyst Fibros*. DOI: 10.1016/j.jcf.2023.08.015.

Woods, JA, Johnson, CE, Allingham, SF, Ngo, HT, Katzenellenbogen, JM & Thompson, SC (2021) Collaborative data familiarisation and quality assessment: Reflections from use of a national dataset to investigate palliative care for Indigenous Australians. *Health Inf Manag* 50:64-75. DOI: 10.1177/1833358320908957.

Wu, D, Xu, H, Yongyi, W & Zhu, H (2022) Quality of government health data in COVID-19: definition and testing of an open government health data quality evaluation framework. *Library Hi Tech* 40(2):516-534. DOI: 10.1108/LHT-04-2021-0126.

Zhang, Y & Koru, G (2020) Understanding and detecting defects in healthcare administration data: Toward higher data quality to better support healthcare operations and decisions. *J Am Med Inform Assoc* 27:386-395. DOI: 10.1093/jamia/ocz201.
