# Supplementary material for: Measuring the Quality of Datasets: Development of the IDEFIM Indicator Set for Empirical Health Research
Source: J Med Internet Res. 2026 Jun 17;28:e90482. doi: 10.2196/90482 (PMC13274964; doi:10.2196/90482)
Supplement: Multimedia Appendix 5 [file jmir-v28-e90482-s005.docx]

Evidence Sources per Indicator in Category Metadata Quality

| **Dimension/indicator group** | **ID** | **Evidence source** |
| --- | --- | --- |
| **Accuracy (metadata)** |  |  |
| Indicators for accuracy (metadata) |  |  |
| Correctness (metadata) | IDEFIM-1062 | Anderka et al. 2015 |
| Responsiveness | IDEFIM-1063 | Couchoud et al. 2013 |
| **Completeness (metadata)** |  |  |
| Indicators for completeness (metadata) |  |  |
| Completeness of administrative metadata | IDEFIM-1064 | Wu et al. 2021 |
| Coverage of all data elements | IDEFIM-1066 | Eder et al. 2021 |
| Data element completeness | IDEFIM-1065 | Tahar et al. 2023 |
| Richness (metadata) | IDEFIM-1067 | Wu et al. 2021 |
| **Compliance (metadata)** |  |  |
| Indicators for compliance (metadata) |  |  |
| Data element compliance with reference | IDEFIM-1047 | Blacketer et al. 2021 |
| Metadata format, type, and unit compliance | IDEFIM-1068 | Liaw et al. 2013, Quindroit et al. 2023, Wu et al. 2021 |
| **Consistency (metadata)** |  |  |
| Indicators for consistency (metadata) |  |  |
| Duplicates (metadata) | IDEFIM-1082 |  |
| Heterogeneous representation of data elements | IDEFIM-1069 | Quindroit et al. 2023 |
| Homonyms (metadata) | IDEFIM-1070 | Quindroit et al. 2023 |
| Synonyms (metadata) | IDEFIM-1071 | Quindroit et al. 2023 |
| **Precision** |  |  |
| Indicators for precision |  |  |
| Granularity (metadata) | IDEFIM-1072 | Eder et al. 2021, Liu et al. 2023 |
| Residual classes of qualitative data elements | IDEFIM-1020 | Stausberg et al. 2023 |
| **Understandability** |  |  |
| Indicators for understandability |  |  |
| Easy of understanding | IDEFIM-1073 | Wu et al. 2021 |
| Relevance of the dataset's descriptive information | IDEFIM-1074 | Wu et al. 2021 |

**References**

Anderka, M, Mai, CT, Romitti, PA, Copeland, G, Isenburg, J, Feldkamp, ML, Krikov, S, Rickard, R, Olney, RS, Canfield, MA, Stanton, C, Mosley, B & Kirby, RS (2015) Development and implementation of the first national data quality standards for population-based birth defects surveillance programs in the United States. *BMC Public Health* 15:925. DOI: 10.1186/s12889-015-2223-2.

Blacketer, C, Defalco, FJ, Ryan, PB & Rijnbeek, PR (2021) Increasing trust in real-world evidence through evaluation of observational data quality. *J Am Med Inform Assoc* 28:2251-2257. DOI: 10.1093/jamia/ocab132.

Couchoud, C, Lassalle, M, Cornet, R & Jager, KJ (2013) Renal replacement therapy registries - time for a structured data quality evaluation programme. *Nephrol Dial Transplant* 28:2215-20. DOI: 10.1093/ndt/gft004.

Eder J & Shekhovtsov VA (2021) Data quality for federated medical data lakes. Int J Web Inf Syst Vol. 17(5):407-426. DOI: 10.1108/IJWIS-03-2021-0026.

Liaw, ST, Rahimi, A, Ray, P, Taggart, J, Dennis, S, de Lusignan, S, Jalaludin, B, Yeo, AE & Talaei-Khoei, A (2013) Towards an ontology for data quality in integrated chronic disease management: a realist review of the literature. *Int J Med Inform* 82:10-24. DOI: 10.1016/j.ijmedinf.2012.10.001.

Liu, C, Talaei-Khoei, A, Storey, VC & Peng, G (2023) A Review of the State of the Art of Data Quality in Healthcare. *J Glob Inf Manag* 31(1):1-18. DOI: 10.4018/JGIM.316236.

Quindroit, P, Fruchart, M, Degoul, S, Perichon, R, Martignène, N, Soula, J, Marcilly, R & Lamer, A (2023) Definition of a Practical Taxonomy for Referencing Data Quality Problems in Health Care Databases. *Methods Inf Med* 62:19-30. DOI: 10.1055/a-1976-2371.

Stausberg, J, Harkener, S, Engel, C, Finger, R, Heinz, C, Jenetzky, E, Jersch, P, Martin, D, Rupp, R, Schoenthaler, M, Suwelack, B & Wegner, J (2023) Cross-Registry Benchmarking of Data Quality: Lessons Learned. *Stud Health Technol Inform* 302:167-171. DOI: 10.3233/shti230096.

Tahar, K, Martin, T, Mou, Y, Verbuecheln, R, Graessner, H & Krefting, D (2023) Rare Diseases in Hospital Information Systems - An Interoperable Methodology for Distributed Data Quality Assessments. *Methods Inf Med* 62:71-89. DOI: 10.1055/a-2006-1018.

Wu, D, Xu, H, Yongyi, W & Zhu, H (2021) Quality of government health data in COVID-19: definition and testing of an open government health data quality evaluation framework. *Library Hi Tech* 40(2):516-534. DOI: 10.1108/LHT-04-2021-0126.
