# Supplementary material for: Measuring the Quality of Datasets: Development of the IDEFIM Indicator Set for Empirical Health Research
Source: J Med Internet Res. 2026 Jun 17;28:e90482. doi: 10.2196/90482 (PMC13274964; doi:10.2196/90482)
Supplement: Multimedia Appendix 6 [file jmir-v28-e90482-s006.pdf]

IDEFIM's Set of Quality Indicators - Specifications

Completeness of administrative metadata

IDEFIM-1064

Section administration

Sequence	
Designation	Completeness of administrative metadata
Designation_inverse	Incompleteness of administrative metadata
Label	CompAdmMetd
Identificator_QI_IDEFIM	IDEFIM-1064
Identificator_external	
URI	
Description	Indicates the degree of completeness of descriptive information such as dataset title, summary and keywords. Complete descriptive information helps users to navigate and to discover data.
Description_terminology	
Structure_superordinate	Completeness (metadata)
Status	draft
Version	0.80
Last_update	13.5.2025
Contact	Prof. Dr. med. J. Stausberg (juergen.stausberg@uk-essen.de), S. Harkener (sonja.harkener@uk-essen.de)
Concept_upper	Indicators for completeness (metadata)
Concept_sub	
Sources	IDEFIM review: wu_2021.
General_notes	

Section References

Property	The calculation is performed for a dataset.

Section Calculation

Calculation_method	1) Determination of which descriptive information is necessary. 2) Determine which descriptive information is available. 3) Calculation of numerator, denominator and rate.
Measurement_function	Numerator: Number of missing descriptive information. Denominator: Number of necessary descriptive information.
Result_specification	rate (without dimension)
Direction	lower values
Threshold	
Influencing_factors	
Notes_calculation	

Section Assessment

Interpretation	

Completeness of administrative metadata

IDEFIM-1064

Notes_assessment

IDEFIM's Set of Quality Indicators - Specifications

Concordance

IDEFIM-1002

Section administration

Sequence	
Designation	Concordance
Designation_inverse	Disconcordance
Label	Concordance
Identificator_QI_IDEFIM	IDEFIM-1002
Identificator_external	TMF-1002
URI	
Description	The correspondence of the value of a data element with a reference source (e. g. patient file, CRF, but not the source of data collection).
Description_terminology	
Structure_superordinate	Consistency (data)
Status	release
Version	0.80
Last_update	2014
Contact	Prof. Dr. med. J. Stausberg (juergen.stausberg@uk-essen.de), S. Harkener (sonja.harkener@uk-essen.de)
Concept_upper	Unexpected entry indicators
Concept_sub	
Sources	TMF guideline 2014: Aronsky/Haug 2000, Brennan/Stead 2000, Cook et al. 2003.
General_notes	The indicator only checks the agreement between two representations of reality and makes no statement about the truthfulness and appropriateness of the representation [Brennan/Stead 2000]. The data from the reference source are assumed to be 'true' when calculating the indicator. If the reference source is identical to the source of data collection, then the indicator corresponds to the indicator 'agreement with source data'. Alternative definition: Concordance can also be seen as a question about the completeness of data collection [Aronsky/Haug 2000], e.g. “Are all diagnoses recorded in the patient file stored in the registry?”.

Section References

Property	The calculation is performed for a dataset. The calculation of the indicator only makes sense for data elements.

Section Calculation

Calculation_method	1) Selection of the reference source. 2) Comparison of the values of the data element with the corresponding values from the reference source. 3) Calculation of numerator, denominator and rate.

Concordance

IDEFIM-1002

Measurement_function	Numerator: Number of values that match the reference source. Denominator: Number of checked values.
Result_specification	rate (without dimension)
Direction	higher values
Threshold	
Influencing_factors	Time and personnel available for data collection; qualification of data collection personnel.
Notes_calculation	

Section Assessment

Interpretation	The lower the rate, the poorer the match with the reference source (and therefore the data quality). Which rate is still acceptable depends on the intended use of the data.
Notes_assessment	

IDEFIM's Set of Quality Indicators - Specifications

Confusion

IDEFIM-1083

Section administration

Sequence	
Designation	Confusion
Designation_inverse	Clearness
Label	Confusion
Identificator_QI_IDEFIM	IDEFIM-1083
Identificator_external	
URI	
Description	Homonyms regarding values in data elements (pieces of information).
Description_terminology	
Structure_superordinate	Consistency (data)
Status	draft
Version	0.80
Last_update	30.6.2025
Contact	Prof. Dr. med. J. Stausberg (juergen.stausberg@uk-essen.de), S. Harkener (sonja.harkener@uk-essen.de)
Concept_upper	Confusion and redundancy indicators
Concept_sub	
Sources	IDEFIM analysis: Woodall et al. 2014.
General_notes	Example: The term 'cervical' appears twice in the Diagnoses data element. It is unclear whether these are two different diagnoses (homonyms, cervical vertebrae or bottom of the uterus) or the same one. Also, the same abbreviations are often used for different diagnoses, e.g. MS for mitral stenosis or multiple sclerosis.

Section References

Property	The calculation can be performed for a single observational unit and for a dataset.

Section Calculation

Calculation_method	1) Search for homonym values. 2) Calculation of numerator, denominator and rate.
Measurement_function	Numerator: Number of homonym values. Denominator: Number of checked values.
Result_specification	rate (without dimension)
Direction	lower values
Threshold	
Influencing_factors	
Notes_calculation	

Confusion

IDEFIM-1083

Section Assessment

Interpretation

Notes_assessment

Conspicuous correctness distribution

IDEFIM-1075

Section administration

Sequence	
Designation	Conspicuous correctness distribution
Designation_inverse	Inconspicuous correctness distribution
Label	DistrCorrect
Identificator_QI_IDEFIM	IDEFIM-1075
Identificator_external	
URI	
Description	Proportion of data elements with a discriminatory misclassification due to diversity, equity, and inclusion demands.
Description_terminology	
Structure_superordinate	Accuracy (data)
Status	draft
Version	0.80
Last_update	7.6.2025
Contact	Prof. Dr. med. J. Stausberg (juergen.stausberg@uk-essen.de), S. Harkener (sonja.harkener@uk-essen.de)
Concept_upper	Contingency table indicators
Concept_sub	
Sources	IDEFIM analysis of DEI issues published partly in Bünz et al 2025.
General_notes	

Section References

Property	The calculation is performed for a dataset.

Section Calculation

Calculation_method	1) Determination of data elements with a discriminatory misclassification due to diversity, equity, and inclusion demands.<br>2) Calculation of numerator, denominator and rate.
Measurement_function	Numerator: Number of data elements with a discriminatory misclassification due to diversity, equity, and inclusion demands.<br>Denominator: Number of all data elements.
Result_specification	rate (without dimension)
Direction	lower values
Threshold	
Influencing_factors	
Notes_calculation	

Section Assessment

Interpretation	
Notes_assessment	

Conspicuous distribution of digits in date-time data elements

IDEFIM-1059

Section administration

Sequence	
Designation	Conspicuous distribution of digits in date-time data elements
Designation_inverse	Inconspicuous distribution of digits in date-time data elements
Label	DistrDigitTD
Identificator_QI_IDEFIM	IDEFIM-1059
Identificator_external	
URI	
Description	Anomalies in the distribution of days and months in dates or digits in time data.
Description_terminology	
Structure_superordinate	Consistency (data)
Status	draft
Version	0.80
Last_update	13.5.2025
Contact	Prof. Dr. med. J. Stausberg (juergen.stausberg@uk-essen.de), S. Harkener (sonja.harkener@uk-essen.de)
Concept_upper	Unexpected entry indicators
Concept_sub	
Sources	IDEFIM review: allemani_2017.
General_notes	

Section References

Property	The calculation is performed for a dataset. The calculation of the indicator only makes sense for date or time data elements.

Section Calculation

Calculation_method	1) Calculation of conspicuous clusters of numbers or digits in selected data elements. 2) Calculation of numerator, denominator and rate.
Measurement_function	Numerator: Number of conspicuous value distributions.<br>Denominator: Number of checked data elements.
Result_specification	rate (without dimension)
Direction	lower values
Threshold	
Influencing_factors	
Notes_calculation	

Section Assessment

Interpretation	
Notes_assessment	

IDEFIM's Set of Quality Indicators - Specifications

Conspicuous distribution of values

IDEFIM-1006

Section administration	
Sequence	
Designation	Conspicuous distribution of values
Designation_inverse	Inconspicuous distribution of values
Label	DistrVal
Identificator_QI_IDEFIM	IDEFIM-1006
Identificator_external	TMF-1006
URI	
Description	Value distributions within and between organizational units for numerical data elements.
Description_terminology	
Structure_superordinate	Consistency (data)
Status	release
Version	0.80
Last_update	2014
Contact	Prof. Dr. med. J. Stausberg (juergen.stausberg@uk-essen.de), S. Harkener (sonja.harkener@uk-essen.de)
Concept_upper	Unexpected entry indicators
Concept_sub	TMF-1007, TMF-1009, TMF-1010, TMF-1011, TMF-1052
Sources	TMF guideline 2014: Hasford/Staib 1994, Schmidt et al. 1995.
General_notes	
**Section References**	
Property	The calculation can be performed for a single observational unit and for a dataset. The calculation of the indicator only makes sense for numerical data elements.
**Section Calculation**	
Calculation_method	1) Calculation of the parameters minimum, maximum, quartiles, mean, median and standard deviation. 2) Determination of reference values from previous data from the organizational unit or data from other organizational units. 3) Comparison of the calculated parameters with the reference values. 4) Calculation of numerator, denominator and rate.
Measurement_function	Numerator: Number of conspicuous value distributions. Denominator: Number of checked data elements.
Result_specification	rate (without dimension)
Direction	lower values
Threshold	
Influencing_factors	Standardization of examination procedures and analysis methods; different patient/person collectives in different organizational

Conspicuous distribution of values

IDEFIM-1006

units.

Notes_calculation

Section Assessment

Interpretation

The higher the rate, the more conspicuous values there are in the data and the poorer the data quality. Every anomaly detected should lead to an in-depth check of the underlying values. A conspicuous distribution can be an indication of problems with examination procedures and analysis methods (e.g. laboratory parameters) or data falsification.

Notes_assessment

IDEFIM's Set of Quality Indicators - Specifications

Conspicuous missing values distribution

IDEFIM-1078

Section administration	
Sequence	
Designation	Conspicuous missing values distribution
Designation_inverse	Inconspicuous missing values distribution
Label	DistrMissVal
Identificator_QI_IDEFIM	IDEFIM-1078
Identificator_external	
URI	
Description	Proportion of data elements with a discriminatory distribution of missing values due to diversity, equity, and inclusion demands.
Description_terminology	
Structure_superordinate	Completeness (data)
Status	draft
Version	0.80
Last_update	7.6.2025
Contact	Prof. Dr. med. J. Stausberg (juergen.stausberg@uk-essen.de), S. Harkener (sonja.harkener@uk-essen.de)
Concept_upper	Missing content indicators
Concept_sub	
Sources	IDEFIM analysis of DEI issues published partly in Bünz et al 2025.
General_notes	
**Section References**	
Property	The calculation is performed for a dataset.
**Section Calculation**	
Calculation_method	1) Identification of the data elements to be analyzed. 2) Determining the distributions of missing values for selected data elements with regard to diversity, equity, and inclusion demands. 3) Calculation of numerator, denominator and rate.
Measurement_function	Numerator: Number of data elements with suspicious distribution. Denominator: Number of data elements checked.
Result_specification	rate (without dimension)
Direction	lower values
Threshold	
Influencing_factors	
Notes_calculation	
**Section Assessment**	
Interpretation	The lower the rate, the fewer data elements there are that have a distorted distribution with regard to diversity, equity, and

Conspicuous missing values distribution

IDEFIM-1078

inclusion demands.

Notes_assessment

IDEFIM's Set of Quality Indicators - Specifications

Conspicuous recruitment rate distribution

IDEFIM-1076

Section administration

Sequence	
Designation	Conspicuous recruitment rate distribution
Designation_inverse	Inconspicuous recruitment rate distribution
Label	DistrRecrRat
Identificator_QI_IDEFIM	IDEFIM-1076
Identificator_external	
URI	
Description	Proportion of underrepresented populations due to diversity, equity, and inclusion demands.
Description_terminology	
Structure_superordinate	Completeness (cases)
Status	draft
Version	0.80
Last_update	7.6.2025
Contact	Prof. Dr. med. J. Stausberg (juergen.stausberg@uk-essen.de), S. Harkener (sonja.harkener@uk-essen.de)
Concept_upper	Indicators for completeness (cases)
Concept_sub	
Sources	IDEFIM analysis of DEI issues published partly in Bünz et al 2025.
General_notes	

Section References

Property	The calculation is performed for a dataset.

Section Calculation

Calculation_method	1) Determination of an appropriate time interval (e.g. 3 months). 2) Compilation of the intended or possible recruitment numbers for each sub-population. 3) Determination of the current recruitment rate for each sub-population. 4) Comparison of the intended or possible recruitment rate with the current rate for each sub-population.
Measurement_function	Numerator: Number of sub-populations with a number of recruited observational units lower than intended or possible. Denominator: Total number of sub-populations.
Result_specification	rate (without dimension)
Direction	lower values
Threshold	
Influencing_factors	
Notes_calculation	

Section Assessment

Conspicuous recruitment rate distribution

IDEFIM-1076

Interpretation

Notes_assessment

IDEFIM's Set of Quality Indicators - Specifications

Conspicuous representativeness distribution

IDEFIM-1077

Section administration

Sequence	
Designation	Conspicuous representativeness distribution
Designation_inverse	Inconspicuous representativeness distribution
Label	DistrRepres
Identificator_QI_IDEFIM	IDEFIM-1077
Identificator_external	
URI	
Description	R-index: a standardized representativeness metric for benchmarking diversity, equity, and inclusion in a dataset.
Description_terminology	
Structure_superordinate	Representativeness
Status	draft
Version	0.80
Last_update	7.6.2025
Contact	Prof. Dr. med. J. Stausberg (juergen.stausberg@uk-essen.de), S. Harkener (sonja.harkener@uk-essen.de)
Concept_upper	Indicators for representativeness
Concept_sub	
Sources	IDEFIM analysis: James et al. 2025.
General_notes	

Section References

Property	The calculation is performed for a dataset.

Section Calculation

Calculation_method	1) Selection of categorical data elements representing DEI dimensions. 2) Determination of a reference population for each data element. 3) Calculation of the proportion for each category within the dataset and within the reference population. 4) Computing the absolute difference for each category between the two proportions.
Measurement_function	Calculating the R-index according to [James 2025] for each data element. Probably building the arithmetic mean or another appropriate distribution parameter for the R-index across data elements.
Result_specification	rate (without dimension)
Direction	higher values
Threshold	
Influencing_factors	
Notes_calculation	

Conspicuous representativeness distribution

IDEFIM-1077

Section Assessment

Interpretation

Notes_assessment

IDEFIM's Set of Quality Indicators - Specifications

Contradictions

IDEFIM-1003

Section administration

Sequence	
Designation	Contradictions
Designation_inverse	Freedom from contradictions
Label	Contrad
Identificator_QI_IDEFIM	IDEFIM-1003
Identificator_external	TMF-1003
URI	
Description	Contradiction is understood as a violation of conditions that are formulated via rules. No distinction is made here between the terms integrity, consistency and plausibility.
Description_terminology	
Structure_superordinate	Consistency (data)
Status	release
Version	0.80
Last_update	23.12.2021
Contact	Prof. Dr. med. J. Stausberg (juergen.stausberg@uk-essen.de), S. Harkener (sonja.harkener@uk-essen.de)
Concept_upper	Contradiction indicators
Concept_sub	TMF-1035, TMF-1004, TMF-1005
Sources	TMF guideline 2014: Bobrowski et al. 1999, Gaus 2003, Jung/Winter 2000, Naumann/Rolker 2000.
General_notes	Data that is as consistent as possible is important for the informative value of analyses. The indicator is not intended for the determination of deviations in the case of duplicate questionnaires. A distinction can be made between possible and certain contradictions. This must be taken into account when establishing the rules.

Section References

Property	The calculation can be performed for a data record, for a single observational unit and for a dataset. It only makes sense to calculate the indicator for data elements.

Section Calculation

Calculation_method	1) Definition of one or more plausibility rules for each selected data element [see also Goertzen/Stausberg 2004]. 2) Apply the plausibility rules to the existing values 3) Calculation of numerator, denominator and rate.
Measurement_function	Numerator: Number of values that violate at least one plausibility rule. Denominator: Number of checked values.
Result_specification	rate/sentinel event (without dimension)

Contradictions

IDEFIM-1003

Direction	lower values
Threshold	0.05
Influencing_factors	Automatic plausibility checks during data entry.
Notes_calculation	These rules can take the following form, for example: B1 and/or B2 and/or B3 ... → A with B1, B2, ..., A: Conditions that describe values or value lists of data elements. Examples: Pregnancy = 'yes' → Gender = 'female'; Age > 50 or gender = 'male' → pregnancy = 'na'; No values are counted for this indicator, but rather the number of plausibility checks performed. Any number of characteristics can be involved in a plausibility rule; each check is included once in the denominator and, if it fails, also in the numerator. If a data element is part of many rules (e.g. sex) and this was recorded incorrectly, this can lead to distortions, as all checks in which this characteristic is involved are then included in the numerator. The check is counted for the specified time period if one of the data elements defined for the rule was collected/recorded in this time period and values are available for all other data elements involved that were also recorded in this time period or earlier.

Section Assessment

Interpretation	The higher the rate, the more contradictions there are in the data and the poorer the data quality and usability of the data. Which rate is still acceptable depends on the intended use of the data.
Notes_assessment	

Correctness

IDEFIM-1037

Section administration	
Sequence	
Designation	Correctness
Designation_inverse	Incorrectness
Label	Correctness
Identificator_QI_IDEFIM	IDEFIM-1037
Identificator_external	TMF-1043
URI	
Description	Correctness of the data (the extent to which the data correspond to the truth).
Description_terminology	The extent to which the data correspond to the truth [Arts et al. 2002a].
Structure_superordinate	Accuracy (data)
Status	release
Version	0.80
Last_update	2014
Contact	Prof. Dr. med. J. Stausberg (juergen.stausberg@uk-essen.de), S. Harkener (sonja.harkener@uk-essen.de)
Concept_upper	Contingency table indicators
Concept_sub	
Sources	TMF guideline 2014: Arts et al. 2002a, Barrie/Marsh 1992, Brennan/Stead 2000, Hassey et al. 2001, Hüsers 2004, Jung/Winter 2000, Kühn-Stoffers 2004, Lindquist 2004, Massey/Hoffman 1989, Prins et al. 2000.
General_notes	The “truth” should come from an independent data source that was not used to collect the data. The comparison of the data with the data sources from which the data were derived is recorded by the indicator “Agreement with source data”. Alternative definition: Accuracy can also be defined as a combination of the indicators 'correctness' and 'completeness' [Brennan/Stead 2000, Hüsers 2004] or as a 'positive predictive value' [Hassey et al. 2001]. The latter is defined as the proportion of patients/persons who actually have a characteristic stored in the dataset out of the total number of patients/persons for whom this characteristic is stored in the dataset.
**Section References**	
Property	The calculation can be performed for a data record, for a single observational unit and for a dataset. Calculating the indicator only makes sense for data elements.
**Section Calculation**	
Calculation_method	1) Abstraction of the “truth” from the independent data source. 2)

Correctness

IDEFIM-1037

	Check whether these values are present in the dataset. 3) Calculation of numerator, denominator and rate.
Measurement_function	Numerator: Number of correct values present in the dataset. Denominator: Number of checked values.
Result_specification	rate (without dimension)
Direction	higher values
Threshold	
Influencing_factors	Time and personnel available for data collection; qualifications of data collection personnel; quality (veracity) of the data sources used to collect the data.
Notes_calculation	

Section Assessment

Interpretation	The higher the rate, the better the data quality. Prins proposes the following classification [Prinset al. 2000]: > 95%: good, between 80% and 95%: moderate, < 80%: poor.
Notes_assessment	

IDEFIM's Set of Quality Indicators - Specifications

Correctness (metadata)

IDEFIM-1062

Section administration	
Sequence	
Designation	Correctness (metadata)
Designation_inverse	Incorrectness (metadata)
Label	CorrectMetd
Identificator_QI_IDEFIM	IDEFIM-1062
Identificator_external	
URI	
Description	Clear and standardized definition of data elements and data element collections.
Description_terminology	
Structure_superordinate	Accuracy (metadata)
Status	draft
Version	0.80
Last_update	13.5.2025
Contact	Prof. Dr. med. J. Stausberg (juergen.stausberg@uk-essen.de), S. Harkener (sonja.harkener@uk-essen.de)
Concept_upper	Indicators for accuracy (metadata)
Concept_sub	
Sources	IDEFIM review: anderka_2015. IDEFIM analyses of DEI issues published partly in Bünz et al 2025.
General_notes	
**Section References**	
Property	The calculation is performed for a dataset.
**Section Calculation**	
Calculation_method	1) Determination of standardized and clear definitions for data elements from an independent data source. 2) Check whether these are used for definitions of data elements. 3) Calculation of numerator, denominator and rate.
Measurement_function	Numerator: Number of standardized and clear definitions of data elements. Denominator: Number of checked definitions of data elements.
Result_specification	rate (without dimension)
Direction	higher values
Threshold	
Influencing_factors	
Notes_calculation	
**Section Assessment**	

IDEFIM's Set of Quality Indicators - Specifications

Correctness (metadata)

IDEFIM-1062

Interpretation

Notes_assessment

IDEFIM's Set of Quality Indicators - Specifications

Coverage of all data elements

IDEFIM-1066

Section administration

Sequence	
Designation	Coverage of all data elements
Designation_inverse	Missing metadata for data elements
Label	CoverAllDE
Identificator_QI_IDEFIM	IDEFIM-1066
Identificator_external	
URI	
Description	All data elements should be supplemented with corresponding metadata.
Description_terminology	
Structure_superordinate	Completeness (metadata)
Status	draft
Version	0.80
Last_update	13.5.2025
Contact	Prof. Dr. med. J. Stausberg (juergen.stausberg@uk-essen.de), S. Harkener (sonja.harkener@uk-essen.de)
Concept_upper	Indicators for completeness (metadata)
Concept_sub	
Sources	IDEFIM review: eder_2021.
General_notes	

Section References

Property	The calculation is performed for a dataset.

Section Calculation

Calculation_method	1) Search for missing metadata for the description of data elements. 2) Calculation of numerator, denominator and rate.
Measurement_function	Numerator: Number of missing metadata for the description of data elements. Denominator: Number of checked data elements.
Result_specification	rate (without dimension)
Direction	lower values
Threshold	
Influencing_factors	
Notes_calculation	

Section Assessment

Interpretation	
Notes_assessment	

IDEFIM's Set of Quality Indicators - Specifications

Currentness

IDEFIM-1024

Section administration

Sequence	
Designation	Currentness
Designation_inverse	Non-currentness
Label	Currentness
Identificator_QI_IDEFIM	IDEFIM-1024
Identificator_external	TMF-1028
URI	
Description	Up-to-dateness of the data.
Description_terminology	
Structure_superordinate	Currentness
Status	release
Version	0.80
Last_update	2014
Contact	Prof. Dr. med. J. Stausberg (juergen.stausberg@uk-essen.de), S. Harkener (sonja.harkener@uk-essen.de)
Concept_upper	Indicators for currentness
Concept_sub	
Sources	TMF guideline 2014: Abate et al. 1998, Bobrowski et al. 1999, Jung/Winter 2000, Katalinic 2005, Kuntoro et al. 1994, Naumann/Rolker 2000, Wang et al. 1993.
General_notes	The indicator is important for the informative value of analyses. Its significance depends on the type of data and the planned use of the data. It is not applicable for unchanging socio-demographic data (e.g. date of birth, place of birth, gender, cause of death) and disease-related data (e.g. congenital diseases and disabilities, diagnosis of chronic and incurable diseases, data from previous hospitalizations, births and miscarriages, transplants, accidents, amputations). In the case of variable disease-related data (e.g. diagnosis, duration, medication and treatment of acute illnesses), it can be used to a limited extent if this data has to be reconstructed from old documents or from the patient's/person's memory.The indicator may be important for feasibility studies if decisions on the inclusion or exclusion of patients in clinical trials are to be made on the basis of variable medical data (e.g. dose changes during the course of treatment at a specified dose interval). The indicator is important as a process indicator for all acute (especially serious) adverse events in the context of a medication or therapy study. It is evidently important for notifiable diseases. Alternative definition: Up-to-dateness can also be understood as the question of the age of stored data [Naumann/Rolker 2000, Wang et al. 1993]. This approach only

	makes sense for data that can change over time and is collected iteratively.

Section References

Property	The calculation can be performed for a data record, for a single observational unit and for a dataset. The calculation only makes sense for variable data elements.

Section Calculation

Calculation_method	1) Determination of the acceptable time difference/expiry time of the data (e.g. 10 days); these values can be different for different data elements or information units. 2) Calculation of the current time difference = date of entry into the data collection - date of the physician contact that generated the data [Katalinic 2005]. 3) Calculation of numerator, denominator and rate.
Measurement_function	Numerator: Number of values with time difference = acceptable time difference. Denominator: Number of checked values.
Result_specification	rate (without dimension)
Direction	higher values
Threshold	
Influencing_factors	Personnel capacities in the organizational units; quality of the technical infrastructure; quality of the data source.
Notes_calculation	Alternative calculation option [Kuntoro et al. 1994]: 1) Determination of an acceptable recency rate 2) Definition of a fixed time interval 3) Calculation of the numerator = number of messages in the specified time interval 3) Calculation of the denominator = expected number of reports in the specified time interval; This value can be derived from known prevalences/incidences. 4) Calculation of the actuality rate 5) Timeliness rate < acceptable timeliness rate means poor data quality. This is similar to the definition of the 'completeness' indicator.

Section Assessment

Interpretation	The lower the rate, the poorer the data quality.
Notes_assessment	

IDEFIM's Set of Quality Indicators - Specifications

Data element completeness

IDEFIM-1065

Section administration

Sequence	
Designation	Data element completeness
Designation_inverse	Missing data elements
Label	DEComplete
Identificator_QI_IDEFIM	IDEFIM-1065
Identificator_external	
URI	
Description	This indicator assesses the metadata completeness of a given dataset and evaluates whether important data elements are considered.
Description_terminology	
Structure_superordinate	Completeness (metadata)
Status	draft
Version	0.80
Last_update	13.5.2025
Contact	Prof. Dr. med. J. Stausberg (juergen.stausberg@uk-essen.de), S. Harkener (sonja.harkener@uk-essen.de)
Concept_upper	Indicators for completeness (metadata)
Concept_sub	
Sources	IDEFIM review: tahar_2023.
General_notes	

Section References

Property	The calculation is performed for a dataset.

Section Calculation

Calculation_method	1) Search for missing data elements in the metadata of the dataset. 2) Calculation of numerator, denominator and rate.
Measurement_function	Numerator: Number of missing data elements. Denominator: Number of all data elements.
Result_specification	rate (without dimension)
Direction	lower values
Threshold	
Influencing_factors	
Notes_calculation	

Section Assessment

Interpretation	
Notes_assessment	

IDEFIM's Set of Quality Indicators - Specifications

Data element compliance with reference

IDEFIM-1047

Section administration	
Sequence	
Designation	Data element compliance with reference
Designation_inverse	Data element incompliance with reference
Label	DEComplRef
Identificator_QI_IDEFIM	IDEFIM-1047
Identificator_external	
URI	
Description	All data elements are present in the dataset as expected based on the specification or present in the metadata as recommended in established standards.
Description_terminology	
Structure_superordinate	Compliance (metadata)
Status	draft
Version	0.80
Last_update	13.5.2025
Contact	Prof. Dr. med. J. Stausberg (juergen.stausberg@uk-essen.de), S. Harkener (sonja.harkener@uk-essen.de)
Concept_upper	Indicators for compliance (metadata)
Concept_sub	
Sources	IDEFIM review: blacketer_2021.
General_notes	
**Section References**	
Property	The calculation is performed for a dataset.
**Section Calculation**	
Calculation_method	1) Selection of the reference source. 2) Comparison of the data elements with the corresponding data elements from the reference source. 3) Calculation of numerator, denominator and rate.
Measurement_function	Numerator: Number of data elements that match the reference source. Denominator: Number of data elements checked.
Result_specification	rate (without dimension)
Direction	higher values
Threshold	
Influencing_factors	
Notes_calculation	
**Section Assessment**	
Interpretation	

Data element compliance with reference

IDEFIM-1047

Notes_assessment

IDEFIM's Set of Quality Indicators - Specifications

Data element contradictions

IDEFIM-1048

Section administration

Sequence	
Designation	Data element contradictions
Designation_inverse	Freedom from data element contradictions
Label	DEContrad
Identificator_QI_IDEFIM	IDEFIM-1048
Identificator_external	
URI	
Description	Inconsistent value for a data element regardless of other data elements (differentiation from contradiction).
Description_terminology	
Structure_superordinate	Consistency (data)
Status	draft
Version	0.80
Last_update	13.5.2025
Contact	Prof. Dr. med. J. Stausberg (juergen.stausberg@uk-essen.de), S. Harkener (sonja.harkener@uk-essen.de)
Concept_upper	Contradiction indicators
Concept_sub	
Sources	IDEFIM review: corrêa_2023, perren_2019.
General_notes	

Section References

Property	The calculation can be performed for a data record, for a single observational unit and for a dataset.

Section Calculation

Calculation_method	1) Definition of permitted values per data element. 2) Search for illegal values. 3) Calculation of numerator, denominator and rate.
Measurement_function	Numerator: Number of missing values. Denominator: Number of checked values.
Result_specification	rate (without dimension)
Direction	lower values
Threshold	
Influencing_factors	
Notes_calculation	

Section Assessment

Interpretation	
Notes_assessment	

IDEFIM's Set of Quality Indicators - Specifications

Data element credibility

IDEFIM-1044

Section administration

Sequence	
Designation	Data element credibility
Designation_inverse	Data element incredibility
Label	DECredibil
Identificator_QI_IDEFIM	IDEFIM-1044
Identificator_external	
URI	
Description	The degree to which data has attributes (e.g. source of the data) that are regarded as true and believable by users in a specific context of use.
Description_terminology	Attributes are properties that describe the data element, for example the provenance of the information for the value of the data element.
Structure_superordinate	Credibility
Status	draft
Version	0.80
Last_update	13.5.2025
Contact	Prof. Dr. med. J. Stausberg (juergen.stausberg@uk-essen.de), S. Harkener (sonja.harkener@uk-essen.de)
Concept_upper	Indicators for credibility
Concept_sub	
Sources	IDEFIM review: gualo_2021, wu_2021.
General_notes	

Section References

Property	The calculation is performed for a dataset.

Section Calculation

Calculation_method	1) Determination of the data element to be checked. 2) Calculation of numerator, denominator and rate.
Measurement_function	Numerator: Number of values that are validated/certified by a specific process. Denominator: Number of values checked.
Result_specification	rate (without dimension)
Direction	higher values
Threshold	
Influencing_factors	
Notes_calculation	

Section Assessment

Interpretation	

Data element credibility

IDEFIM-1044

Notes_assessment

IDEFIM's Set of Quality Indicators - Specifications

Data elements with existing entries for all observational units

IDEFIM-1014

Section administration

Sequence	
Designation	Data elements with existing entries for all observational units
Designation_inverse	Data elements with at least one missing entry for all observation units
Label	DEExEntry
Identificator_QI_IDEFIM	IDEFIM-1014
Identificator_external	TMF-1017
URI	
Description	Proportion of data elements with existing entries for all observational units.
Description_terminology	
Structure_superordinate	Completeness (data)
Status	release
Version	0.80
Last_update	2014
Contact	Prof. Dr. med. J. Stausberg (juergen.stausberg@uk-essen.de), S. Harkener (sonja.harkener@uk-essen.de)
Concept_upper	Other indicators for completeness (data)
Concept_sub	The indicator is a subcategory of 'Missing entries'.
Sources	TMF guideline 2014: Open European Nephrology Science Center (OpEN.SC, TMF project V020-04).
General_notes	

Section References

Property	The calculation is performed for a dataset.

Section Calculation

Calculation_method	1) Determination of the data elements to be included. 2) Determine for each data element whether values from all observational units are available. 3) Calculation of numerator, denominator and rate.
Measurement_function	Numerator: Number of data elements with entries for all observational units. Denominator: Number of data elements checked.
Result_specification	rate (without dimension)
Direction	higher values
Threshold	
Influencing_factors	The influencing factors are manifold, as this indicator combines aspects of other key figures. It therefore makes sense not to analyze this indicator separately, but together with others from

Data elements with existing entries for all observational units

IDEFIM-1014

	the 'Missing entries' area.
Notes_calculation	The calculation is useful for both optional and mandatory data elements. The handling of entries such as 'unknown' or similar must be defined.The handling of entries such as 'unknown' or similar must be defined.

Section Assessment

Interpretation	The lower the rate, the poorer the data quality. Which rate is still acceptable depends on the intended use of the data.
Notes_assessment	

IDEFIM's Set of Quality Indicators - Specifications

Data elements with value unknown etc.

IDEFIM-1013

Section administration	
Sequence	
Designation	Data elements with value unknown etc.
Designation_inverse	Data elements without value unknown etc.
Label	ValUnknown
Identificator_QI_IDEFIM	IDEFIM-1013
Identificator_external	TMF-1016
URI	
Description	Proportion of data elements with value unknown or similar meaning.
Description_terminology	
Structure_superordinate	Consistency (data)
Status	release
Version	0.80
Last_update	2014
Contact	Prof. Dr. med. J. Stausberg (juergen.stausberg@uk-essen.de), S. Harkener (sonja.harkener@uk-essen.de)
Concept_upper	Unexpected entry indicators
Concept_sub	The indicator is a subcategory of 'Missing entries'.
Sources	TMF guideline 2014: Study in Health in Pomerania (SHIP, TMF project V020-04).
General_notes	This indicator is only useful for mandatory data elements with a predefined list of values where an entry is unknown or similar. This indicator is related to the indicator TMF-1025 'Data elements with unspecific values'.

Section References	
Property	The calculation can be performed for a data record, for a single observational unit and for a dataset.

Section Calculation	
Calculation_method	1) Definition of the relevant designations ('unknown' or similar). 2) Search for the corresponding entries. 3) Calculation of numerator, denominator and rate.
Measurement_function	Numerator: Number of values unknown or similar. Denominator: Number of checked values.
Result_specification	rate (without dimension)
Direction	lower values
Threshold	
Influencing_factors	Motivation of the survey personnel, type of survey.
Notes_calculation	

Data elements with value unknown etc.

IDEFIM-1013

Section Assessment

Interpretation	The higher the rate, the poorer the data quality. Which rate is still acceptable depends on the intended use of the data.
Notes_assessment	

IDEFIM's Set of Quality Indicators - Specifications

Data format, data type, and unit compliance

IDEFIM-1057

Section administration	
Sequence	
Designation	Data format, data type, and unit compliance
Designation_inverse	Data format, data type, or unit incompliance
Label	FormTypeData
Identificator_QI_IDEFIM	IDEFIM-1057
Identificator_external	
URI	
Description	Number of values that do not comply with data format, data type or unit defined for the data element.
Description_terminology	
Structure_superordinate	Compliance (data)
Status	draft
Version	0.80
Last_update	13.5.2025
Contact	Prof. Dr. med. J. Stausberg (juergen.stausberg@uk-essen.de), S. Harkener (sonja.harkener@uk-essen.de)
Concept_upper	Indicators for compliance (data)
Concept_sub	
Sources	IDEFIM review: aerts_2021, eder_2021, elouataoui_2022, gisslander_2023, gualo_2021, kahn_2018, lee_2018, oh_2023, quindroit_2023, schmidt_2021, shaheen_2019, skyttberg_2017, zhang_2020.
General_notes	This indicator can be divided into 3 sub-indicators so that the data is analyzed either for deviations for a format for a type or for a unit.
**Section References**	
Property	The calculation can be performed for a data record, for a single observational unit and for a dataset. The calculation of the indicator only makes sense for data elements for which a specific format or a specific type or unit is defined.
**Section Calculation**	
Calculation_method	1) Determination of the data elements to be included. 2) Determine for each data element whether the format, type or unit complies with the specification. 3) Calculation of numerator, denominator and rate.
Measurement_function	Numerator: Number of compliant values. Denominator: Number of checked values.
Result_specification	rate (without dimension)
Direction	higher values

Data format, data type, and unit compliance

IDEFIM-1057

Threshold

Influencing_factors

Notes_calculation

Section Assessment

Interpretation

Notes_assessment

IDEFIM's Set of Quality Indicators - Specifications

Disagreement with previous values

IDEFIM-1001

Section administration

Sequence	
Designation	Disagreement with previous values
Designation_inverse	Agreement with previous values
Label	DisagPrevVal
Identificator_QI_IDEFIM	IDEFIM-1001
Identificator_external	TMF-1001
URI	
Description	Disagreement of the result of a measurement or examination with the previous value.
Description_terminology	Previous value: A value of the same data element collected at an earlier point in time in relation to the current value.
Structure_superordinate	Consistency (data)
Status	release
Version	0.80
Last_update	2014
Contact	Prof. Dr. med. J. Stausberg (juergen.stausberg@uk-essen.de), S. Harkener (sonja.harkener@uk-essen.de)
Concept_upper	Unexpected entry indicators
Concept_sub	
Sources	TMF guideline 2014: Gassmann et al. 1995, Naumann/Rolker 2000, O'Kelly 2004.
General_notes	This indicator supports the visualization of data errors. Values that differ implausibly from their previous values are a clear indication of the existence of a data error. The indicator is related to the 'reliability' indicator. A distinction must be made here between: - unchanging characteristics that should not change at all (e.g. date of birth) or only within the limits of measurement accuracy (e.g. height), and - variable characteristics, for which a deviation from the previous value may occur or is to be expected.

Section References

Property	The calculation can be performed for a single observational unit and for a dataset. Calculating the indicator only makes sense for data elements that are collected multiple times (e.g. laboratory parameters, vital signs).

Section Calculation

Calculation_method	1) For data elements that should not change (within the scope of the measurement accuracy): Determination of the acceptable deviation per data element, taking into account the variability of the measurements (e.g. '0' for date of birth, '1 cm' for height). For variable data elements: Determination of the acceptable deviation

Disagreement with previous values

IDEFIM-1001

	per data element that is plausible under the given conditions of the data collection. 2) Calculation of the absolute difference to the previous value = amount (current value - previous value). 3) Calculation of numerator, denominator and rate.
Measurement_function	Numerator: Number of values with absolute difference to the previous value > acceptable deviation. Denominator: Number of values checked.
Result_specification	rate (without dimension)
Direction	lower values
Threshold	
Influencing_factors	Standardization of examination procedures and analysis methods; one-off recording of non-variable characteristics.
Notes_calculation	

Section Assessment

Interpretation	The higher the rate, the more implausible values (potential data errors) there are and the poorer the data quality. Which rate is still acceptable depends on the intended use of the data.
Notes_assessment	

Disagreement with source data referring to data elements

IDEFIM-1038

Section administration

Sequence	
Designation	Disagreement with source data referring to data elements
Designation_inverse	Agreement with source data referring to data elements
Label	DisagSDataDE
Identificator_QI_IDEFIM	IDEFIM-1038
Identificator_external	TMF-1044
URI	
Description	Disagreement of the data with the original data in relation to data elements.
Description_terminology	Original data: The data from which the data was extracted. The original data can exist in paper form or as electronic documents.
Structure_superordinate	Accuracy (data)
Status	release
Version	0.80
Last_update	2014
Contact	Prof. Dr. med. J. Stausberg (juergen.stausberg@uk-essen.de), S. Harkener (sonja.harkener@uk-essen.de)
Concept_upper	Disagreement with source data indicators
Concept_sub	
Sources	TMF guideline 2014: Arts et al. 2001, Arts et al. 2002a, Califf et al. 1997, Cnattingius et al. 1990, Gissler et al. 1996, Håkansson et al. 2001, Jensen et al. 2002, Khosla et al. 2000, Lin et al. 2004, Lu et al. 1995, Mullooly 1990, Maruszewksi et al. 2005, Nielsen et al. 1996, Ose et al. 2004, O'Sullivan et al. 1996, Teperi 1993, Pogash et al. 2001, Schmidt et al. 1995, Vestberg et al. 1997, Weiss et al. 1993, Weiss 1998.
General_notes	This indicator represents the result of a source data verification (original data comparison) and is also required for the case number planning of the source data verification. The indicator only checks whether the data matches the original data and does not contain any statement about the truth of the data. The original data is assumed to be 'true' when calculating the indicator. If the data do not match, they can only be data errors or missing values. The indicator is related to the indicators 'Agreement of the data with the original data in relation to observational units', 'Concordance' and 'Correctness'.

Section References

Property	The calculation is performed for a dataset. The calculation of the indicator only makes sense for data elements.

Section Calculation

Disagreement with source data referring to data elements

IDEFIM-1038

Calculation_method	1) Comparison of the values of the data element with the corresponding values from the original data. 2) Calculation of numerator, denominator and rate.
Measurement_function	Numerator: Number of values that do not match the original data. Denominator: Number of checked values.
Result_specification	rate (without dimension)
Direction	lower values
Threshold	
Influencing_factors	Time and personnel available for data collection; qualification of data collection personnel; second collection of data independent of initial collection.
Notes_calculation	

Section Assessment

Interpretation	The higher the rate, the worse the match with the original data (and therefore the data quality). Which rate is still acceptable depends on the intended use of the data; a high rate may indicate problems with the collection or recording of the data.
Notes_assessment	

IDEFIM's Set of Quality Indicators - Specifications

Disagreement with source data referring to observational units

IDEFIM-1039

Section administration

Sequence	
Designation	Disagreement with source data referring to observational units
Designation_inverse	Agreement with source data referring to observational units
Label	DisagSDataOU
Identificator_QI_IDEFIM	IDEFIM-1039
Identificator_external	TMF-1045
URI	
Description	Inconsistency of the data with the original data in relation to observational units.
Description_terminology	Original data: The data from which the data was extracted. The original data can exist in paper form or as electronic documents.
Structure_superordinate	Accuracy (data)
Status	release
Version	0.80
Last_update	2014
Contact	Prof. Dr. med. J. Stausberg (juergen.stausberg@uk-essen.de), S. Harkener (sonja.harkener@uk-essen.de)
Concept_upper	Disagreement with source data indicators
Concept_sub	
Sources	TMF guideline 2014: Arts et al. 2001, Arts et al. 2002a, Califf et al. 1997, Cnattingius et al. 1990, Gissler et al. 1996, Håkansson et al. 2001, Jensen et al. 2002, Khosla et al. 2000, Lin et al. 2004, Lu et al. 1995, Mullooly 1990, Maruszewksi et al. 2005, Nielsen et al. 1996, Ose et al. 2004, O'Sullivan et al. 1996, Teperi 1993, Pogash et al. 2001, Schmidt et al. 1995, Vestberg et al. 1997, Weiss et al. 1993, Weiss 1998.
General_notes	This indicator represents the result of a source data verification (original data comparison) and is also required for the case number planning of the source data verification. The indicator only checks whether the data matches the original data and does not contain any statement about the truth of the data. The original data is assumed to be 'true' when calculating the indicator. If the data do not match, they can only be data errors or missing values.The indicator is related to the indicators 'Agreement of the data with the original data in relation to data elements', 'Concordance' and 'Correctness'.

Section References

Property	The calculation is performed for a dataset. The calculation of the indicator only makes sense for data elements.

Section Calculation

Disagreement with source data referring to observational units

IDEFIM-1039

Calculation_method	1) Definition of the observational unit (e.g. patient, report, report form). 2) Determination of the number of errors for each observational unit. 3) Calculation of numerator, denominator and rate.
Measurement_function	Numerator: Number of observational units with at least one error. Denominator: Number of observational units checked.
Result_specification	rate (without dimension)
Direction	lower values
Threshold	
Influencing_factors	Time and personnel available for data collection; qualification of data collection personnel; second collection of data independent of initial collection.
Notes_calculation	

Section Assessment

Interpretation	The higher the rate, the worse the match with the original data (and thus the data quality). Which rate is still acceptable depends on the intended use of the data. A high rate may indicate problems with the collection or recording of the data.
Notes_assessment	

IDEFIM's Set of Quality Indicators - Specifications

Drop-out-rate

IDEFIM-1030

Section administration

Sequence	
Designation	Drop-out-rate
Designation_inverse	Continous participation rate
Label	DropOutRate
Identificator_QI_IDEFIM	IDEFIM-1030
Identificator_external	TMF-1034
URI	
Description	Number of observational units who leave a data collection prematurely.
Description_terminology	Premature termination: From a data management perspective, the premature termination of data collection for a patient/person in the dataset. This can happen by withdrawing consent to participate in the data collection or by breaking off contact between the organizational unit and the patient/person (lost-to-follow-up).
Structure_superordinate	Completeness (cases)
Status	release
Version	0.80
Last_update	23.12.2021
Contact	Prof. Dr. med. J. Stausberg (juergen.stausberg@uk-essen.de), S. Harkener (sonja.harkener@uk-essen.de)
Concept_upper	Indicators for completeness (cases)
Concept_sub	
Sources	TMF guideline 2014: IMIBE Expertise.
General_notes	The lowest possible drop-out rate is important for the informative value of analyses. The indicator is only important for follow-up data collections and for data collections from which patients are to be recruited for clinical studies (feasibility studies). However, it can also be a qualitative medical indicator (e.g. congenital disease or disability - time of death or time of therapy - time of death) or say something about the 'level of consent' and the satisfaction of patients/persons with the data collection (e.g. examinations are too time-consuming, painful or unpleasant, 'nothing happens'). In the case of age-related diseases, the number of deaths that go unreported is difficult to estimate.

Section References

Property	The calculation is performed for a dataset.

Section Calculation

Calculation_method	1) Definition of an appropriate time interval (e.g. 3 months). 2) Calculation of numerator, denominator and rate.

IDEFIM's Set of Quality Indicators - Specifications

Drop-out-rate

IDEFIM-1030

Measurement_function	Numerator: Number of patients/persons who leave the data collection prematurely within the selected time interval. Denominator: Number of new patients/persons added to the data collection in the selected time interval.
Result_specification	ratio (without dimension)
Direction	lower values
Threshold	0.01
Influencing_factors	Measures to maintain and improve contact between the organizational unit and patients/individuals; informing patients/individuals about the purpose of the data collection; personnel capacity in the organizational units (consistency).
Notes_calculation	Alternative definition: patients/persons who leave the data collection due to death can be excluded from the numerator. The point of time at which a patient/person is considered to be a drop-out must be defined. This is particularly difficult for data collectiosn without regular examinations or survey intervals.

Section Assessment

Interpretation	Drop-out rate > threshold value is an indication of problems in connection with patient approach and contact and can also be an indication of manipulated data. Since (too) many data/disease progressions were only incompletely recorded, the overall quality and informative value of the data is reduced. In the Parkinson's Competence Network, a drop-out rate of 0.013 (62 of 4,801 patients) was empirically determined as of 15 November 2005 (by G. Antony, IT Coordination Parkinson's Competence Network). Of the 62 patients, 42 had died, the remaining 20 patients withdrew their consent for further participation in the data collection.
Notes_assessment	

Duplicates (data)

IDEFIM-1025

Section administration

Sequence	
Designation	Duplicates (data)
Designation_inverse	Freedom from duplicates (data)
Label	DuplicData
Identificator_QI_IDEFIM	IDEFIM-1025
Identificator_external	TMF-1029
URI	
Description	Number of duplicates in the dataset.
Description_terminology	Duplicate: Two or more information units that describe the same subject or object and for which the identity can be proven or - if proof is not possible - can be assumed with sufficient probability. Sufficient probability: A probability that is considered sufficient taking into account data quality aspects and the intended use of the data.
Structure_superordinate	Consistency (data)
Status	release
Version	0.80
Last_update	9.11.2018
Contact	Prof. Dr. med. J. Stausberg (juergen.stausberg@uk-essen.de), S. Harkener (sonja.harkener@uk-essen.de)
Concept_upper	Confusion and redundancy indicators
Concept_sub	
Sources	TMF guideline 2014: Swart/Ihle 2005.
General_notes	The indicator is important for the correct estimation of incidences and prevalences and is related to the 'number of synonyms' indicator. The existence of a duplicate means that data is actually available several times. In the case of synonyms, on the other hand, a patient/person is listed several times under different patient identifiers. However, this does not necessarily mean that his/her data is available twice. For example, a documentation assistant could have started recording data on a new patient after obtaining a patient identifier and a second documentation assistant could continue recording data under this new patient indicator after accidentally obtaining a second patient identifier.The estimated rate of duplicates must always be included in the evaluation of statistical indicators (significance level).

Section References

Property	The calculation can be performed for a single observational unit and for a dataset. The calculation only makes sense for definable

Duplicates (data)

IDEFIM-1025

	information units (e.g. diagnoses).
**Section Calculation**	
Calculation_method	1) Definition of a set of data elements whose combination allows the detection of duplicates (e.g. gender, date of birth, place of residence, date of infection, procedures). 2) Definition of the detection limit for the duplicate search (e.g. matching values for all data elements in the set; matching values for at least 6 of 8 data elements in the set). 3) Identification of duplicates by comparing the values of the key elements per information unit. 4) Calculation of numerator, denominator and rate.
Measurement_function	Numerator: Number of duplicates found. Denominator: Number of checked sets of data elements.
Result_specification	rate/sentinel event (without dimension)
Direction	lower values
Threshold	0.05
Influencing_factors	Merging of data from different information sources in the data collection; patients/persons who visit several reporting organizational units; special type of disease (e.g. dementia); age (children); quality of the technical infrastructure (possibility of reporting organizational unit changes and parallel treatment of a patient/person in several organizational units); number of patients/persons recruited in an organizational unit (doctors cannot remember all patients/persons); fluctuation in the organizational unit (the new doctor does not know whether his predecessor has already seen the patient/person).
Notes_calculation	It must first be investigated where duplicates may occur in the database.
**Section Assessment**	
Interpretation	The higher the rate, the poorer the data quality. Swart/Ihle 2005 describe a rate of < 0.01 as acceptable for secondary data analysis of routine healthcare data; this rate appears too rigid and not in line with reality for large datasets. From an empirical sample of 700 patients from a center of the Parkinson's Competence Network, a realistic rate of 2.5% can be estimated (determined by G. Antony, IT Coordination Parkinson's Competence Network).
Notes_assessment	

Duplicates (metadata)

IDEFIM-1082

Section administration

Sequence	
Designation	Duplicates (metadata)
Designation_inverse	Freedom from duplicates (metadata)
Label	DuplicMetd
Identificator_QI_IDEFIM	IDEFIM-1082
Identificator_external	
URI	
Description	Identical designation of data elements in different datasets.
Description_terminology	
Structure_superordinate	Consistency (metadata)
Status	draft
Version	0.80
Last_update	30.6.2025
Contact	Prof. Dr. med. J. Stausberg (juergen.stausberg@uk-essen.de), S. Harkener (sonja.harkener@uk-essen.de)
Concept_upper	Indicators for consistency (metadata)
Concept_sub	
Sources	IDEFIM analysis: Woodall et al. 2014.
General_notes	

Section References

Property	The calculation can be performed for multiple datasets.

Section Calculation

Calculation_method	1) Search for identical designation of data elements in different datasets. 2) Calculation of numerator, denominator and rate.
Measurement_function	Numerator: Number of duplicates found. Denominator: Number of checked data elements.
Result_specification	rate/sentinel event (without dimension)
Direction	lower values
Threshold	
Influencing_factors	
Notes_calculation	

Section Assessment

Interpretation	
Notes_assessment	

IDEFIM's Set of Quality Indicators - Specifications

Easy of understanding

IDEFIM-1073

Section administration

Sequence	
Designation	Easy of understanding
Designation_inverse	Difficult of understanding
Label	EasyUnderst
Identificator_QI_IDEFIM	IDEFIM-1073
Identificator_external	
URI	
Description	Indicates whether the dataset's descriptive information is easy to understand without unclear or ambiguous representations.
Description_terminology	
Structure_superordinate	Understandability
Status	draft
Version	0.80
Last_update	13.5.2025
Contact	Prof. Dr. med. J. Stausberg (juergen.stausberg@uk-essen.de), S. Harkener (sonja.harkener@uk-essen.de)
Concept_upper	Indicators for understandability
Concept_sub	
Sources	IDEFIM review: wu_2021.
General_notes	

Section References

Property	The calculation is performed for a dataset.

Section Calculation

Calculation_method	1) Search for unclear or ambiguous representations. 2) Determination of numerator, denominator and rate.
Measurement_function	Numerator: Number of unclear or ambiguous representations. Denominator: Number checked representations.
Result_specification	rate (without dimension)
Direction	lower values
Threshold	
Influencing_factors	
Notes_calculation	

Section Assessment

Interpretation	
Notes_assessment	

IDEFIM's Set of Quality Indicators - Specifications

Frequency outliers

IDEFIM-1046

Section administration

Sequence	
Designation	Frequency outliers
Designation_inverse	No frequency outliers
Label	FreqOutliers
Identificator_QI_IDEFIM	IDEFIM-1046
Identificator_external	
URI	
Description	Unusual or conspicuous results in a frequency analysis.
Description_terminology	
Structure_superordinate	Consistency (data)
Status	draft
Version	0.80
Last_update	13.5.2025
Contact	Prof. Dr. med. J. Stausberg (juergen.stausberg@uk-essen.de), S. Harkener (sonja.harkener@uk-essen.de)
Concept_upper	Unexpected entry indicators
Concept_sub	
Sources	IDEFIM review: khare_2017.
General_notes	This indicator is not about a numerical outlier, but about how often a data element that is collected at different points in time occurs. For example an observational unit with 3000 procedures or a laboratory culture result with 10 organisms.

Section References

Property	The calculation can be performed for a single observational unit and for a dataset. The calculation of the indicator only makes sense for numerical data elements.

Section Calculation

Calculation_method	1) Frequency analysis for selected data elements. 2) Search for conspicuous results. 3) Calculation of numerator, denominator and rate.
Measurement_function	Numerator: Number of conspicuous results. Denominator: Number of results.
Result_specification	rate (without dimension)
Direction	lower values
Threshold	
Influencing_factors	
Notes_calculation	

Frequency outliers

IDEFIM-1046

Section Assessment

Interpretation

Notes_assessment

IDEFIM's Set of Quality Indicators - Specifications

Granularity (data)

IDEFIM-1050

Section administration

Sequence	
Designation	Granularity (data)
Designation_inverse	Coarseness (data)
Label	GranularData
Identificator_QI_IDEFIM	IDEFIM-1050
Identificator_external	
URI	
Description	The degree of detail of data elements.
Description_terminology	
Structure_superordinate	Accuracy (data)
Status	draft
Version	0.80
Last_update	13.5.2025
Contact	Prof. Dr. med. J. Stausberg (juergen.stausberg@uk-essen.de), S. Harkener (sonja.harkener@uk-essen.de)
Concept_upper	Other indicators for accuracy (data)
Concept_sub	
Sources	IDEFIM review: eder_2021, feder_2018.
General_notes	For qualitative data elements, the degree of detail is, for example, the number of used categories out of all available categories; for numerical data elements, it is, for example, the number of significant digits.

Section References

Property	The calculation is performed for a dataset.

Section Calculation

Calculation_method	1) Definition of the data elements to be analyzed. 2) Definition of the highest possible degree of detail for the data elements to be analyzed. 3) Determination of numerator, denominator and rate.
Measurement_function	Numerator: Number of data elements with the highest possible level of detail. Denominator: Number of checked data elements.
Result_specification	rate (without dimension)
Direction	higher values
Threshold	
Influencing_factors	
Notes_calculation	

Section Assessment

Interpretation	

Granularity (data)

IDEFIM-1050

Notes_assessment

Granularity (metadata)

IDEFIM-1072

Section administration	
Sequence	
Designation	Granularity (metadata)
Designation_inverse	Coarseness (metadata)
Label	GranularMetd
Identificator_QI_IDEFIM	IDEFIM-1072
Identificator_external	
URI	
Description	The degree of detail of a data element (e.g. the number of used categories for the values of categorical data elements or the number of significant digits for the values of numerical data elements).
Description_terminology	
Structure_superordinate	Precision
Status	draft
Version	0.80
Last_update	13.5.2025
Contact	Prof. Dr. med. J. Stausberg (juergen.stausberg@uk-essen.de), S. Harkener (sonja.harkener@uk-essen.de)
Concept_upper	Indicators for precision
Concept_sub	
Sources	IDEFIM review: eder_2021, liu_2023. IDEFIM analyses of DEI issues published partly in Bünz et al 2025.
General_notes	

Section References	
Property	The calculation is performed for a dataset.

Section Calculation	
Calculation_method	1) Selection of data elements for which a level of granularity can be defined. 2) Determination of a desired level of detail for each data element, e.g. based on an external standard. 3) Comparison of the level of detail in the metadata with the desired level of detail.
Measurement_function	Numerator: Number of data elements with the desired granularity. Denominator: Number of data elements selected.
Result_specification	rate (without dimension)
Direction	higher values
Threshold	
Influencing_factors	
Notes_calculation	

Granularity (metadata)

IDEFIM-1072

Section Assessment

Interpretation

Notes_assessment

IDEFIM's Set of Quality Indicators - Specifications

Heterogeneous representation of data elements

IDEFIM-1069

Section administration

Sequence	
Designation	Heterogeneous representation of data elements
Designation_inverse	Homogeneous representation of data elements
Label	HeteroReprDE
Identificator_QI_IDEFIM	IDEFIM-1069
Identificator_external	
URI	
Description	Different representations of the same data element in different datasets.
Description_terminology	
Structure_superordinate	Consistency (metadata)
Status	draft
Version	0.80
Last_update	13.5.2025
Contact	Prof. Dr. med. J. Stausberg (juergen.stausberg@uk-essen.de), S. Harkener (sonja.harkener@uk-essen.de)
Concept_upper	Indicators for consistency (metadata)
Concept_sub	
Sources	IDEFIM review: quindroit_2023.
General_notes	Unlike with synonyms (metadata), where the focus is on the designation of data elements, here the focus is on different representations of data elements. For example: In the dataset 1, information related to the address of the patient is stored in the ADDRESS column of the PATIENT table, while four distinct columns of the PAT table in the dataset 2 may store the ADDRESS, POSTAL_CODE, CITY, and COUNTRY.

Section References

Property	The calculation is performed for multiple datasets.

Section Calculation

Calculation_method	1) Search for data elements whose representation differs in the datasets. 2) Calculation of numerator, denominator and rate.
Measurement_function	Numerator: Number of data elements whose representation differs in the datasets. Denominator: Number of data elements checked.
Result_specification	rate (without dimension)
Direction	lower values
Threshold	
Influencing_factors	

Heterogeneous representation of data elements

IDEFIM-1069

Notes_calculation

Section Assessment

Interpretation

Notes_assessment

IDEFIM's Set of Quality Indicators - Specifications

Homonyms (data)

IDEFIM-1032

Section administration	
Sequence	
Designation	Homonyms (data)
Designation_inverse	Freedom from homonyms (data)
Label	HomonymData
Identificator_QI_IDEFIM	IDEFIM-1032
Identificator_external	TMF-1037
URI	
Description	Number of homonyms in observational units.
Description_terminology	Homonym: Two different definitions are associated with one word. In the context of datasets, a patient identifier is referred to as a homonym if it has been assigned to more than one patient/person.
Structure_superordinate	Consistency (data)
Status	release
Version	0.80
Last_update	2014
Contact	Prof. Dr. med. J. Stausberg (juergen.stausberg@uk-essen.de), S. Harkener (sonja.harkener@uk-essen.de)
Concept_upper	Confusion and redundancy indicators
Concept_sub	
Sources	TMF guideline 2014: Parkin/Muir 1992, Winter et al. 2003.
General_notes	The indicator is important for the correct estimation of incidences and prevalences. Unrecognized homonyms lead to data from different patients/persons being incorrectly combined. This can also lead to supposedly incorrect or contradictory data and thus negatively influence the assessment of data quality. The detection of homonyms is difficult and may only be possible to a limited extent or not at all.
**Section References**	
Property	The calculation is performed for a dataset. If possible, all patients/persons should be included in the search for homonyms.
**Section Calculation**	
Calculation_method	1) Definition of the criteria by which homonyms can be recognized (e.g. the data elements used to generate the patient identifiers, duplicate examinations, start of therapy before diagnosis, therapy does not match the diagnosis). 2) Identification of homonyms by checking the defined criteria. 3) Calculation of numerator, denominator and rate.
Measurement_function	Numerator: Number of homonyms. Denominator: Number of

Homonyms (data)

IDEFIM-1032

	patients/persons checked.
Result_specification	rate (without dimension)
Direction	lower values
Threshold	
Influencing_factors	Algorithm for generating patient identifiers.
Notes_calculation	

Section Assessment

Interpretation	The higher the rate, the poorer the data quality. Which rate is still acceptable depends on the intended use of the data.
Notes_assessment	

IDEFIM's Set of Quality Indicators - Specifications

Homonyms (metadata)

IDEFIM-1070

Section administration

Sequence	
Designation	Homonyms (metadata)
Designation_inverse	Freedom from homonyms (metadata)
Label	HomonymMetd
Identificator_QI_IDEFIM	IDEFIM-1070
Identificator_external	
URI	
Description	The same designation is used for different data elements in multiple datasets.
Description_terminology	
Structure_superordinate	Consistency (metadata)
Status	draft
Version	0.80
Last_update	13.5.2025
Contact	Prof. Dr. med. J. Stausberg (juergen.stausberg@uk-essen.de), S. Harkener (sonja.harkener@uk-essen.de)
Concept_upper	Indicators for consistency (metadata)
Concept_sub	
Sources	IDEFIM review: quindroit_2023.
General_notes	For example: in the datasets 1 and 2, UNIT corresponds to a measurement unit and a hospital unit, respectively.

Section References

Property	The calculation is performed for multiple datasets.

Section Calculation

Calculation_method	1) Definition of the criteria by which homonyms can be recognized. 2) Identification of homonyms by checking the defined criteria. 3) Calculation of numerator, denominator and rate.
Measurement_function	Numerator: Number of homonyms. Denominator: Number of data elements checked.
Result_specification	rate (without dimension)
Direction	lower values
Threshold	
Influencing_factors	
Notes_calculation	

Section Assessment

Interpretation	

Homonyms (metadata)

IDEFIM-1070

Notes_assessment

IDEFIM's Set of Quality Indicators - Specifications

Illegal values of qualitative data elements

IDEFIM-1017

Section administration

Sequence	
Designation	Illegal values of qualitative data elements
Designation_inverse	Permissible values of qualitative data elements
Label	IllegQualDE
Identificator_QI_IDEFIM	IDEFIM-1017
Identificator_external	TMF-1021
URI	
Description	Qualitative data elements with values that do not originate from a defined value set.
Description_terminology	Qualitative data element: A data element that can only accept a finite number of qualitative values, i.e. values that cannot be quantified (e.g. diagnosis coded according to ICD-10).
Structure_superordinate	Accuracy (data)
Status	release
Version	0.80
Last_update	2014
Contact	Prof. Dr. med. J. Stausberg (juergen.stausberg@uk-essen.de), S. Harkener (sonja.harkener@uk-essen.de)
Concept_upper	Illegal content indicators
Concept_sub	The indicator is a subcategory of 'Illegal values'. Depending on the
Sources	TMF guideline 2014: Gaus 2003, Swart/Ihle 2005, Winter et al. 2003.
General_notes	This indicator only makes sense if it is technically possible to record illegal values at all. For data elements that contain coded values, the indicator only makes a statement about the formal correctness of the coding (e.g. permitted value range = all existing ICD-10 codes). The (difficult) problem of the correctness of the content of a code is not covered by this indicator.

Section References

Property	The calculation can be performed for a data record, for a single observational unit and for a dataset. Calculating the indicator only makes sense for qualitative data elements.

Section Calculation

Calculation_method	1) Definition of permitted values per data element. 2) Search for illegal values. 3) Calculation of numerator, denominator and rate.
Measurement_function	Numerator: Number of illegal values. Denominator: Number of checked values.
Result_specification	rate (without dimension)
Direction	lower values

Illegal values of qualitative data elements

IDEFIM-1017

Threshold	
Influencing_factors	Automatic area checks during data capture.
Notes_calculation	

Section Assessment

Interpretation	Every illegal value is a data error. The higher the rate, the poorer the data quality; the acceptable rate depends on the intended use of the data.
Notes_assessment	

IDEFIM's Set of Quality Indicators - Specifications

Illegal values of qualitative data elements used for the coding of missings

IDEFIM-1018

Section administration

Sequence	
Designation	Illegal values of qualitative data elements used for the coding of missings
Designation_inverse	Permissible values of qualitative data elements used for the coding of missings
Label	IllegCodgMis
Identificator_QI_IDEFIM	IDEFIM-1018
Identificator_external	TMF-1022
URI	
Description	Number of illegal values for qualitative data elements for the coding of missings.
Description_terminology	
Structure_superordinate	Accuracy (data)
Status	release
Version	0.80
Last_update	2014
Contact	Prof. Dr. med. J. Stausberg (juergen.stausberg@uk-essen.de), S. Harkener (sonja.harkener@uk-essen.de)
Concept_upper	Illegal content indicators
Concept_sub	The indicator is a subcategory of 'Illegal values'.
Sources	TMF guideline 2014: Study in Health in Pomerania (SHIP, TMF project V020-04).
General_notes	This indicator only makes sense if the occurrence of missings is technically possible and coding is provided.

Section References

Property	The calculation can be performed for a data record, for a single observational unit and for a dataset. The calculation of the indicator only makes sense for qualitative data elements.

Section Calculation

Calculation_method	1) Definition of the permitted values for missings per data element. 2) Search for illegal values. 3) Calculation of numerator, denominator and rate.
Measurement_function	Numerator: Number of illegal values. Denominator: Number of checked values.
Result_specification	rate (without dimension)
Direction	lower values
Threshold	
Influencing_factors	Automatic assignment of missings with defined codes.

Illegal values of qualitative data elements used for the coding of missings

IDEFIM-1018

Notes_calculation

Section Assessment

Interpretation	Every illegal value is a data error. The higher the rate, the poorer the data quality.
Notes_assessment	

IDEFIM's Set of Quality Indicators - Specifications

Incompliance with metadata

IDEFIM-1058

Section administration

Sequence	
Designation	Incompliance with metadata
Designation_inverse	Compliance with metadata
Label	IncomplMetd
Identificator_QI_IDEFIM	IDEFIM-1058
Identificator_external	
URI	
Description	Comparison of existing values with their specifications, e.g. from a data dictionary.
Description_terminology	
Structure_superordinate	Compliance (data)
Status	draft
Version	0.80
Last_update	13.5.2025
Contact	Prof. Dr. med. J. Stausberg (juergen.stausberg@uk-essen.de), S. Harkener (sonja.harkener@uk-essen.de)
Concept_upper	Indicators for compliance (data)
Concept_sub	
Sources	IDEFIM review: brown_2013.
General_notes	

Section References

Property	The calculation is performed for a dataset.

Section Calculation

Calculation_method	1) Search for data elements whose specification differs from the metadata. 2) Calculation of numerator, denominator and rate.
Measurement_function	Numerator: Number of data elements whose specification differs from the metadata. Denominator: Number of data elements checked.
Result_specification	rate (without dimension)
Direction	lower values
Threshold	
Influencing_factors	
Notes_calculation	

Section Assessment

Interpretation	
Notes_assessment	

Incorrect text in qualitative data elements

IDEFIM-1051

Section administration

Sequence	
Designation	Incorrect text in qualitative data elements
Designation_inverse	Correct text in qualitative data elements
Label	IncorrText
Identificator_QI_IDEFIM	IDEFIM-1051
Identificator_external	
URI	
Description	Number of values with spelling errors, typos, or misleading abbreviations.
Description_terminology	
Structure_superordinate	Accuracy (data)
Status	draft
Version	0.80
Last_update	13.5.2025
Contact	Prof. Dr. med. J. Stausberg (juergen.stausberg@uk-essen.de), S. Harkener (sonja.harkener@uk-essen.de)
Concept_upper	Illegal content indicators
Concept_sub	
Sources	IDEFIM review: elouataoui_2022, gualo_2021, quindroit_2023, zhang_2020.
General_notes	

Section References

Property	The calculation can be performed for a data record, for a single observational unit and for a dataset. Calculating the indicator only makes sense for qualitative data elements.

Section Calculation

Calculation_method	1) Search for spelling errors, typos, or misleading abbreviations. 2) Calculation of numerator, denominator and rate.
Measurement_function	Numerator: Number of values with spelling errors, typos, or misleading abbreviations. Denominator: Number of checked values.
Result_specification	rate (without dimension)
Direction	lower values
Threshold	
Influencing_factors	
Notes_calculation	

Section Assessment

Incorrect text in qualitative data elements

IDEFIM-1051

Interpretation

Notes_assessment

IDEFIM's Set of Quality Indicators - Specifications

Information density score

IDEFIM-1056

Section administration

Sequence	
Designation	Information density score
Designation_inverse	-
Label	InfDensScore
Identificator_QI_IDEFIM	IDEFIM-1056
Identificator_external	
URI	
Description	The average amount of information each observation provides for a patient observed n times. Higher scores indicate that patient measurements were more equally distributed across patient visits.
Description_terminology	
Structure_superordinate	Completeness (data)
Status	draft
Version	0.80
Last_update	13.5.2025
Contact	Prof. Dr. med. J. Stausberg (juergen.stausberg@uk-essen.de), S. Harkener (sonja.harkener@uk-essen.de)
Concept_upper	Other indicators for completeness (data)
Concept_sub	
Sources	IDEFIM review: wiley_2022. IDEFIM analysis: Sperrin et al. 2011.
General_notes	Wiley: Information density scores are measures of completeness that account for the irregular nature of patient measurements taken over time. Information density is the average amount of information each observation provides for a patient observed n times. An information density score is a number between 0 and 1 where higher scores indicate that patient measurements were more equally distributed across patient visits. Sperrin: The information score can be interpreted as a measure of the average quantity of information provided by each observation in an individual’s time course, where information is lost whenever the observation density deviates from a defined optimal density.

Section References

Property	The calculation can be performed for a single observational unit and for a dataset.

Section Calculation

Calculation_method	1) Selection of data elements with recurrent entries. 2) Determination of the information density score [Sperrin 2011] for each data element and each observational unit using the following information about a data element and an observational unit: number of entries, survey date of each entry in any unit of time

Information density score

IDEFIM-1056

	(e.g. days, hours, minutes, seconds).
Measurement_function	Building the arithmetic mean or another appropriate distribution parameter across observational units for each selected data element. Probably building the arithmetic mean or another appropriate distribution parameter for a dataset across all arithmetic means for data elements.
Result_specification	rate (without dimension)
Direction	higher result
Threshold	
Influencing_factors	
Notes_calculation	

Section Assessment

Interpretation	An information density score is a number between 0 and 1 where higher scores indicate that patient measurements were more equally distributed across patient visits.
Notes_assessment	

IDEFIM's Set of Quality Indicators - Specifications

Last digit preferences

IDEFIM-1007

Section administration

Sequence	
Designation	Last digit preferences
Designation_inverse	No last digit preferences
Label	LastDigPref
Identificator_QI_IDEFIM	IDEFIM-1007
Identificator_external	TMF-1007
URI	
Description	Preference for certain final digits for numerical data elements.
Description_terminology	Final digit: The rightmost digit of a value. The position of the decimal point is irrelevant (e.g. the values '10.43' and '213' have both the final digit '3'; the value '213.0' has the final digit '0').
Structure_superordinate	Consistency (data)
Status	release
Version	0.80
Last_update	2014
Contact	Prof. Dr. med. J. Stausberg (juergen.stausberg@uk-essen.de), S. Harkener (sonja.harkener@uk-essen.de)
Concept_upper	Unexpected entry indicators
Concept_sub	This indicator is a subcategory of TMF-1006.
Sources	TMF guideline 2014: Hasford/Staib 1994, O'Kelly 2004.
General_notes	The indicator is based on the assumption that every digit in the range from 0 to 9 can occur as a final digit and that the probability of occurrence is the same for all digits (e.g. if the body weight is measured with an accuracy of 500 g in 'kg', then only '0' and '5' can occur as final digits). If these conditions are not met, the indicator should not be used. The smaller the number of verified values, the greater the frequency differences in the final digits can be, even with correct data.

Section References

Property	The calculation is performed for a dataset. The calculation of the indicator only makes sense for numerical data elements.

Section Calculation

Calculation_method	1) Calculation of the frequency of occurrence of the final digits 0 to 9 per selected data element. 2) Examination of the frequencies found per data element for conspicuous (uneven) distribution of the final digits. 3) Calculation of numerator, denominator and rate.
Measurement_function	Numerator: Number of data elements with random distribution of end digits. Denominator: Number of checked data elements.
Result_specification	rate (without dimension)

Last digit preferences

IDEFIM-1007

Direction	lower values
Threshold	
Influencing_factors	Standardization of examination procedures and analysis methods.
Notes_calculation	

Section Assessment

Interpretation	The higher the rate, the poorer the data quality. Conspicuous differences can be an indication of problems with examination procedures and analysis methods (e.g. laboratory parameters) or of data falsification and should result in an in-depth check of the underlying values.
Notes_assessment	

IDEFIM's Set of Quality Indicators - Specifications

Metadata format, type, and unit compliance

IDEFIM-1068

Section administration

Sequence	
Designation	Metadata format, type, and unit compliance
Designation_inverse	Metadata format, type, or unit incompliance
Label	FormTypeMetd
Identificator_QI_IDEFIM	IDEFIM-1068
Identificator_external	
URI	
Description	Use of uniform data formats, data types, and units in multiple datasets.
Description_terminology	
Structure_superordinate	Compliance (metadata)
Status	draft
Version	0.80
Last_update	13.5.2025
Contact	Prof. Dr. med. J. Stausberg (juergen.stausberg@uk-essen.de), S. Harkener (sonja.harkener@uk-essen.de)
Concept_upper	Indicators for compliance (metadata)
Concept_sub	
Sources	IDEFIM review: liaw_2013, quindroit_2023, wu_2021.
General_notes	Examples: In the dataset 1, the BIRTH_DATE has the format DD/MM/YYYY, while in the dataset 2, the it has the format YYYY/MM/DD. The data type for the PAT_ID is VARCHAR2(20) in one dataset and CHAR(10) in the other. The unit for HEIGHT is expressed by centimeters in one dataset and in meters in the other.

Section References

Property	The calculation is performed for multiple datasets.

Section Calculation

Calculation_method	1) Determination of the data elements to be included. 2) Determine for each data element whether the format, type or unit complies with the specification of other datasets. 3) Calculation of numerator, denominator and rate.
Measurement_function	Numerator: Number of compliant data elements. Denominator: Number of checked data elements.
Result_specification	rate (without dimension)
Direction	higher values
Threshold	
Influencing_factors	

Metadata format, type, and unit compliance

IDEFIM-1068

Notes_calculation

Section Assessment

Interpretation

Notes_assessment

Misfielded values

IDEFIM-1081

Section administration

Sequence	
Designation	Misfielded values
Designation_inverse	Correctly assigned values
Label	MisfieldVal
Identificator_QI_IDEFIM	IDEFIM-1081
Identificator_external	
URI	
Description	Values that correspond to the expected value of another data element. Refers to qualitative data elements that do not have a defined value set.
Description_terminology	
Structure_superordinate	Accuracy (data)
Status	draft
Version	0.80
Last_update	30.6.2025
Contact	Prof. Dr. med. J. Stausberg (juergen.stausberg@uk-essen.de), S. Harkener (sonja.harkener@uk-essen.de)
Concept_upper	Illegal content indicators
Concept_sub	
Sources	IDEFIM review: khare_2017.pdf, quindroit_2023.pdf, zhang_2020.pdf.
General_notes	This is about correctly written entries which are however not permitted for the data element, e.g. a diagnosis in the field for an operation.

Section References

Property	The calculation can be performed for a data record, for a single observational unit and for a dataset. Calculating the indicator only makes sense for qualitative data elements.

Section Calculation

Calculation_method	1) Definition of permitted values per data element. 2) Search for illegal text. 3) Calculation of numerator, denominator and rate.
Measurement_function	Numerator: Number of values with illegal text. Denominator: Number of checked values.
Result_specification	rate (without dimension)
Direction	lower values
Threshold	
Influencing_factors	
Notes_calculation	

Misfielded values

IDEFIM-1081

Section Assessment

Interpretation

Notes_assessment

Missing evidence of known correlations

IDEFIM-1022

Section administration

Sequence	
Designation	Missing evidence of known correlations
Designation_inverse	Evidence of known correlations
Label	KnownCorrel
Identificator_QI_IDEFIM	IDEFIM-1022
Identificator_external	TMF-1027
URI	
Description	Proof of an already known correlation between different data elements.
Description_terminology	Correlation: Existence of a statistical relationship between two or more quantitative data elements.
Structure_superordinate	Consistency (data)
Status	release
Version	0.80
Last_update	2014
Contact	Prof. Dr. med. J. Stausberg (juergen.stausberg@uk-essen.de), S. Harkener (sonja.harkener@uk-essen.de)
Concept_upper	Other indicators for consistency (data)
Concept_sub	
Sources	TMF guideline 2014: Hasford/Staib 1994.
General_notes	

Section References

Property	The calculation is performed for a dataset. The calculation of the indicator only makes sense for quantitative data elements.

Section Calculation

Calculation_method	1) Determination of data elements that should be correlated with each other. 2) Determination of the statistical method for checking the correlation. 3) Application of the selected procedure to the selected data elements. 4) Calculation of numerator, denominator and rate.
Measurement_function	Numerator: Number of expected but undetectable correlations. Denominator: Number of correlations checked.
Result_specification	rate (without dimension)
Direction	lower values
Threshold	
Influencing_factors	
Notes_calculation	

Missing evidence of known correlations

IDEFIM-1022

Section Assessment

Interpretation	The higher the rate, the poorer the data quality. If the proof of an expected correlation fails, this is an indication of data errors or data falsification (and thus poor data quality). These cases should be checked in detail.
Notes_assessment	

IDEFIM's Set of Quality Indicators - Specifications

Missing modules

IDEFIM-1009

Section administration

Sequence	
Designation	Missing modules
Designation_inverse	Existing modules
Label	MissMod
Identificator_QI_IDEFIM	IDEFIM-1009
Identificator_external	TMF-1012
URI	
Description	A dataset can be divided into modules, e.g. according to procedures, to factual references such as socio-demographics or to groups of related data elements for example used in a specific report. This indicator checks the completeness of the modules.
Description_terminology	
Structure_superordinate	Completeness (data)
Status	release
Version	0.80
Last_update	2014
Contact	Prof. Dr. med. J. Stausberg (juergen.stausberg@uk-essen.de), S. Harkener (sonja.harkener@uk-essen.de)
Concept_upper	Missing content indicators
Concept_sub	The indicator is a subcategory of 'Missing entries'.
Sources	TMF guideline 2014: Study in Health in Pomerania (SHIP, TMF project V020-04).
General_notes	

Section References

Property	The calculation can be performed for a single observational unit and for a dataset.

Section Calculation

Calculation_method	1) Search for missing modules. 2) Calculation of numerator, denominator and rate.
Measurement_function	Numerator: Number of missing modules. Denominator: Number of modules checked.
Result_specification	rate (without dimension)
Direction	lower values
Threshold	
Influencing_factors	Motivation of study participants, burden on study participants due to examinations, device failures, study design.
Notes_calculation	This indicator can be limited to one module, such as sociodemography, as well as including various modules. Only

	complete modules are analyzed; missing modules may be due to a lack of compliance on the part of study participants with regard to certain examinations, for example.
**Section Assessment**	
Interpretation	The higher the rate, the poorer the data quality. Which rate is still acceptable depends on the intended use of the data.
Notes_assessment	

IDEFIM's Set of Quality Indicators - Specifications

Missing values in data elements

IDEFIM-1010

Section administration	
Sequence	
Designation	Missing values in data elements
Designation_inverse	Existing values in data elements
Label	MissValDE
Identificator_QI_IDEFIM	IDEFIM-1010
Identificator_external	TMF-1013
URI	
Description	Number of missing values for data elements.
Description_terminology	
Structure_superordinate	Completeness (data)
Status	release
Version	0.80
Last_update	9.11.2018
Contact	Prof. Dr. med. J. Stausberg (juergen.stausberg@uk-essen.de), S. Harkener (sonja.harkener@uk-essen.de)
Concept_upper	Missing content indicators
Concept_sub	The indicator is a subcategory of 'Missing entries'. Subcategories of
Sources	TMF guideline 2014: Addition for reasons of systematics (TMF project V020-04).
General_notes	This indicator was introduced to enable calculation of the number of missing values including optional and mandatory data elements.

Section References	
Property	The calculation can be performed for a data record, for a single observational unit and for a dataset.

Section Calculation	
Calculation_method	1) Search for missing values. 2) Calculation of numerator, denominator and rate.
Measurement_function	Numerator: Number of missing values. Denominator: Number of checked values.
Result_specification	rate (without dimension)
Direction	lower values
Threshold	0.05
Influencing_factors	Introduction of separate categories for 'not applicable', 'not done'. Use of an EDC system with forced entries for mandatory data elements.
Notes_calculation	Only include data records that are marked as “completed” (i.e. no open queries available); Differentiate “not applicable”, “not done”, “unknown” from “missing” or evaluate them

	additionally/separately.
**Section Assessment**	
Interpretation	The higher the rate, the poorer the data quality. Which rate is still acceptable depends on the intended use of the data.
Notes_assessment	There are data elements that must be recorded (mandatory) and data elements that are optional. The assessment of the indicator should take into account whether mandatory or optional data elements were examined. In addition, the following distinctions regarding missing values could be examined: data missing complete at random (Missings are randomly distributed within the data, not associated with other variables, and independent of unobserved data. For example, data are considered MCAR if due to a clinician forgetting to document a patient's BMI in the EHR). Data missing not at random (Missing data are related to the unobserved value of the missing variable. For example, data would be MNAR if data were missing for all patients with critical levels of cardiac enzymes). Data missing at random (If the missing data are associated with other variables, but are independent of unobserved data values. For example, data would be MAR if males were less likely to answer a screening question about anxiety, but variable missingness did not depend on reported anxiety levels.).

Modules with existing entries for all data elements

IDEFIM-1045

**Section administration**	
Sequence	
Designation	Modules with existing entries for all data elements
Designation_inverse	Modules with missing entry for at least one data element
Label	ModComplete
Identificator_QI_IDEFIM	IDEFIM-1045
Identificator_external	
URI	
Description	Assesses the completeness of values required for recording the module.
Description_terminology	
Structure_superordinate	Completeness (data)
Status	draft
Version	0.80
Last_update	13.5.2025
Contact	Prof. Dr. med. J. Stausberg (juergen.stausberg@uk-essen.de), S. Harkener (sonja.harkener@uk-essen.de)
Concept_upper	Other indicators for completeness (data)
Concept_sub	
Sources	IDEFIM review: tahar_2023.
General_notes	
**Section References**	
Property	The calculation can be performed for a single observational unit and for a dataset.
**Section Calculation**	
Calculation_method	1) Search for complete modules (existing entries for all data elements). 2) Calculation of numerator, denominator and rate.
Measurement_function	Numerator: Number of complete modules. Denominator: Number of modules checked.
Result_specification	rate (without dimension)
Direction	higher values
Threshold	
Influencing_factors	
Notes_calculation	
**Section Assessment**	
Interpretation	
Notes_assessment	

IDEFIM's Set of Quality Indicators - Specifications

Observational units with existing entries for all data elements

IDEFIM-1049

Section administration

Sequence	
Designation	Observational units with existing entries for all data elements
Designation_inverse	Observation units with at least one missing entry for a data element
Label	ObsUExEntry
Identificator_QI_IDEFIM	IDEFIM-1049
Identificator_external	
URI	
Description	Completeness of all required data elements for an observational unit.
Description_terminology	
Structure_superordinate	Completeness (data)
Status	draft
Version	0.80
Last_update	13.5.2025
Contact	Prof. Dr. med. J. Stausberg (juergen.stausberg@uk-essen.de), S. Harkener (sonja.harkener@uk-essen.de)
Concept_upper	Other indicators for completeness (data)
Concept_sub	
Sources	IDEFIM review: garcía-de-león-chocano_2015, gualo_2021, perren_2019, tahar_2023.
General_notes	

Section References

Property	The calculation can be performed for a single observational unit and for a dataset.

Section Calculation

Calculation_method	1) Search for complete observational units (existing entries for all data elements). 2) Calculation of numerator, denominator and rate.
Measurement_function	Numerator: Number of complete observational units.<br>Denominator: Number of observational units checked.
Result_specification	rate (without dimension)
Direction	higher values
Threshold	
Influencing_factors	
Notes_calculation	

Section Assessment

Observational units with existing entries for all data elements

IDEFIM-1049

Interpretation

Notes_assessment

IDEFIM's Set of Quality Indicators - Specifications

Observational units with follow-up

IDEFIM-1036

Section administration

Sequence	
Designation	Observational units with follow-up
Designation_inverse	Observational units without follow-up
Label	ObsUFU
Identificator_QI_IDEFIM	IDEFIM-1036
Identificator_external	TMF-1042
URI	
Description	Proportion of observational units with follow-up.
Description_terminology	
Structure_superordinate	Completeness (data)
Status	release
Version	0.80
Last_update	23.12.2021
Contact	Prof. Dr. med. J. Stausberg (juergen.stausberg@uk-essen.de), S. Harkener (sonja.harkener@uk-essen.de)
Concept_upper	Other indicators for completeness (data)
Concept_sub	
Sources	TMF guideline 2014: Gesellschaft der epidemiologischen Krebsregister in Deutschland e.V. (GEKID) Hentschel/Katalinic 2008 (TMF project V020-04).
General_notes	This indicator says something about the completeness of follow-ups. Follow-ups are important for follow-up examinations (e.g. determining the survival time after the initial diagnosis of a tumor), which requires knowledge of the follow-ups to be carried out.

Section References

Property	The calculation is performed for a dataset.

Section Calculation

Calculation_method	1) Identification of the observational units for which at least one follow-up is available. 2) Determination of numerator, denominator and rate.
Measurement_function	Numerator: Number of observational units with follow-up. Denominator: Number of observational units checked.
Result_specification	rate (without dimension)
Direction	higher values
Threshold	0.9 (REGISVF-AP)
Influencing_factors	Willingness of reporting organizational units to report, patient mobility.

Observational units with follow-up

IDEFIM-1036

Notes_calculation	The patients/persons for whom a follow-up should be available in the period under consideration according to the project specification must be identified. In particular, consideration must be given to how to deal with the time margins that may have been defined for the permissible survey time of an FU (e.g. ± 30 days). It must also be taken into account that the recording of an FU in the database can only take place at a later point in time (interval between survey and recording).

Section Assessment

Interpretation	The lower the rate, the poorer the data quality.
Notes_assessment	

IDEFIM's Set of Quality Indicators - Specifications

Outliers (continuous data elements)

IDEFIM-1015

Section administration	
Sequence	
Designation	Outliers (continuous data elements)
Designation_inverse	No outliers (continuous data elements)
Label	Outliers
Identificator_QI_IDEFIM	IDEFIM-1015
Identificator_external	TMF-1018
URI	
Description	Outliers for continuous data elements, i.e. values outside the interval of expected or plausible values.
Description_terminology	Continuous data element: A data element that can assume any intermediate value within a certain range (e.g. blood pressure). Outlier: Value of a data element that does not meet expectations, i.e. lies outside the interval of expected or plausible values.
Structure_superordinate	Consistency (data)
Status	release
Version	0.80
Last_update	14.10.2019
Contact	Prof. Dr. med. J. Stausberg (juergen.stausberg@uk-essen.de), S. Harkener (sonja.harkener@uk-essen.de)
Concept_upper	Unexpected entry indicators
Concept_sub	Possible depending on the data element, e.g. gender for laborator
Sources	TMF guideline 2014: Gaus 2003, Swart/Ihle 2005.
General_notes	This indicator can be used specifically for laboratory parameters and vital signs. Not every outlier is a data error, e.g. very high or very low laboratory values can occur depending on the severity of a disease and the condition of a patient.

Section References	
Property	The calculation can be performed for a data record, for a single observational unit and for a dataset. The calculation of the indicator only makes sense for continuous data elements.

Section Calculation	
Calculation_method	1) Definition of the interval of expected values per data element. 2) Search for values that lie outside this interval. 3) Calculation of numerator, denominator and rate.
Measurement_function	Numerator: Number of outliers. Denominator: Number of checked values.
Result_specification	rate/sentinel event (without dimension)
Direction	lower values

Outliers (continuous data elements)

IDEFIM-1015

Threshold	0.1
Influencing_factors	Standardization of examination procedures and analysis methods.
Notes_calculation	An interval used in the presentation of box-whisker plots is 1.5 times the interquartile range, i.e. 1.5 times the distance between the 1st and 3rd quartile. Definition of which data elements are continuous. Definition of an interval for each data element so that a value outside this interval qualifies as an outlier.

Section Assessment

Interpretation	The higher the rate, the more outliers and therefore data errors there are in the data. A high rate can be an indication of problems with the underlying examination or analysis methods.
Notes_assessment	

IDEFIM's Set of Quality Indicators - Specifications

Outliers in numerical data elements in a multivariate analysis

IDEFIM-1061

Section administration

Sequence	
Designation	Outliers in numerical data elements in a multivariate analysis
Designation_inverse	No outliers in numerical data elements in a multivariate analysis
Label	OutlMultiVar
Identificator_QI_IDEFIM	IDEFIM-1061
Identificator_external	
URI	
Description	Computing the Mahalanobis distance of at least two data elements and counting the number of extreme measurements.
Description_terminology	
Structure_superordinate	Consistency (data)
Status	draft
Version	0.80
Last_update	13.5.2025
Contact	Prof. Dr. med. J. Stausberg (juergen.stausberg@uk-essen.de), S. Harkener (sonja.harkener@uk-essen.de)
Concept_upper	Unexpected entry indicators
Concept_sub	
Sources	IDEFIM review: schmidt_2021.
General_notes	

Section References

Property	The calculation can be performed for a data record, for a single observational unit and for a dataset. The calculation of the indicator only makes sense for continuous data elements.

Section Calculation

Calculation_method	1) Determination of at least two data elements. 2) Computing the Mahalanobis distance and counting the number of extreme measurements. 3) Calculation of numerator, denominator and rate.
Measurement_function	Numerator: number of extreme measurements. Denominator: number of normal measurements.
Result_specification	rate/sentinel event (without dimension)
Direction	lower values
Threshold	
Influencing_factors	
Notes_calculation	

Section Assessment

Outliers in numerical data elements in a multivariate analysis

IDEFIM-1061

Interpretation

Notes_assessment

Range compliance

IDEFIM-1043

Section administration

Sequence	
Designation	Range compliance
Designation_inverse	Range incompliance
Label	RangeCompl
Identificator_QI_IDEFIM	IDEFIM-1043
Identificator_external	
URI	
Description	Number of values that lie within a specific range defined for the data element.
Description_terminology	
Structure_superordinate	Compliance (data)
Status	draft
Version	0.80
Last_update	13.5.2025
Contact	Prof. Dr. med. J. Stausberg (juergen.stausberg@uk-essen.de), S. Harkener (sonja.harkener@uk-essen.de)
Concept_upper	Indicators for compliance (data)
Concept_sub	
Sources	IDEFIM review: blacketer_2021, garcía-de-león-chocano_2015, gualo_2021, kalincik_2017, oh_2023, schmidt_2021, tute_2023, zhang_2020.
General_notes	Differentiation from outliers: Here, no outliers in a distribution are considered, but values that do not lie within a defined range.

Section References

Property	The calculation can be performed for a data record, for a single observational unit and for a dataset.

Section Calculation

Calculation_method	1) Definition of a permitted range per data element. 2) Search for values that lie within the defined range. 3) Calculation of numerator, denominator and rate.
Measurement_function	Numerator: Number of values that lie within the defined range. Denominator: Number of checked values.
Result_specification	rate (without dimension)
Direction	higher values
Threshold	
Influencing_factors	
Notes_calculation	

Range compliance

IDEFIM-1043

Section Assessment

Interpretation

Notes_assessment

IDEFIM's Set of Quality Indicators - Specifications

Recall

IDEFIM-1040

Section administration

Sequence	
Designation	Recall
Designation_inverse	-
Label	Recall
Identificator_QI_IDEFIM	IDEFIM-1040
Identificator_external	TMF-1046
URI	
Description	The proportion of observations made about the world that were recorded in the dataset (also known as sensitivity).
Description_terminology	Extent to which data that could be recorded had been really recorded [Arts et al. 2002a]. Formerly referred to as completeness.
Structure_superordinate	Accuracy (data)
Status	release
Version	0.80
Last_update	14.10.2019
Contact	Prof. Dr. med. J. Stausberg (juergen.stausberg@uk-essen.de), S. Harkener (sonja.harkener@uk-essen.de)
Concept_upper	Contingency table indicators
Concept_sub	
Sources	TMF guideline 2014: Arts et al. 2002a, Barrie/Marsh 1992, Bobrowski et al. 1999, Goldberg et al. 1980, Hassey et al. 2001, Hogan/Wagner 1997, Jensen et al. 2002, Jung/Winter 2000, Katalinic 2005, Kuntoro et al. 1994, Lindquist 2004, Logan et al. 2001, Naumann/Rolker 2000, Nielsen et al. 1996, Parkin/Muir 1992, Teppo et al. 1994, Topp et al. 1997, Vestberg et al. 1997.
General_notes	This indicator is important for the correct estimation of incidences and prevalences. It is related to the indicators 'concordance' and 'correctness'. The completeness of recruitment is measured by the 'recruitment rate' indicator. Other registries, death certificates, health insurance billing data, etc. can be used as sources for determining the data that could be included in the dataset. Alternative definition: Completeness can also be defined as the proportion of patients/persons with complete data or as sensitivity [Hassey et al. 2001, Nielsen et al. 1996], i.e. as the proportion of patients/persons who actually have a characteristic stored in the dataset among all patients/persons who have this characteristic in reality.

Section References

Property	The calculation can be performed for a data record, for a single observational unit and for a dataset. The calculation of the

Recall

IDEFIM-1040

	indicator only makes sense for data elements.
**Section Calculation**	
Calculation_method	1) Identification of data that should be recorded. 2) Check whether the data is available. 3) Calculation of numerator, denominator, and rate.
Measurement_function	Numerator: Number of available values [Naumann/Rolker 2000]. Denominator: Number of available values + Number of missing values (= Number of values, that could be recorded) [Naumann/Rolker 2000].
Result_specification	rate (without dimension)
Direction	higher values
Threshold	0.95
Influencing_factors	Time and personnel available for data collection; qualification of the data collection personnel; completeness of the original documents on which the data is based.
Notes_calculation	
**Section Assessment**	
Interpretation	The higher the rate, the more complete the data and the better the data quality. Which rate is still acceptable depends on the intended use of the data.
Notes_assessment	

IDEFIM's Set of Quality Indicators - Specifications

Recruitment rate

IDEFIM-1026

Section administration

Sequence	
Designation	Recruitment rate
Designation_inverse	Failed recruitment rate
Label	RecruitRate
Identificator_QI_IDEFIM	IDEFIM-1026
Identificator_external	TMF-1030
URI	
Description	Ratio between recruited and recruitable persons or assessment of the achievement of a recruitment target.
Description_terminology	Recruitment: Inclusion of patients/persons who fulfil the inclusion and exclusion criteria in a dataset. Inclusion criteria: Set of characteristics of a patient/person that must all be present at the same time for him/her to be included in the data collection (e.g. presence of a specific disease). Exclusion criteria: Set of characteristics of a patient/person that do not allow his/her inclusion in the data collection. The presence of at least one characteristic prohibits the inclusion of the patient/person in the data collection (e.g. age < 18 years).
Structure_superordinate	Completeness (cases)
Status	release
Version	0.80
Last_update	23.12.2021
Contact	Prof. Dr. med. J. Stausberg (juergen.stausberg@uk-essen.de), S. Harkener (sonja.harkener@uk-essen.de)
Concept_upper	Indicators for completeness (cases)
Concept_sub	TMF-1051
Sources	TMF guideline 2014: Goldberg et al. 1980, Hasford/Staib 1994, Kuntoro et al. 1994, Pogash et al. 2001, Pollock 1994, Svolba/Bauer 1999.
General_notes	A recruitment target could be set for each participating organizational unit. This can be done based on the number of cases in the organizational unit or by using a checklist of all potential subjects or by consulting other references such as hospital statistics or the hospital's quality reports.

Section References

Property	The calculation is performed for a dataset.

Section Calculation

Calculation_method	1) Determination of an appropriate time interval (e.g. 3 months).<br>2) Calculation of numerator, denominator and rate/ratio.

IDEFIM's Set of Quality Indicators - Specifications

Recruitment rate

IDEFIM-1026

Measurement_function	Numerator: Number of patients/persons recruited in the selected time interval. Denominator: Number of 'potentially suitable' (i.e. recruitable) patients/persons in the selected time interval; alternative measurement: Numerator: Number of patients/persons recruited in the selected time interval. Denominator: Number of patients/persons to be recruited in the selected time interval according to a sample size estimate (recruitment target).
Result_specification	rate (without dimension), if comparision with a number of 'potentially suitable patients/persons; ratio (without dimension), if comparsion with a recruitment target.
Direction	higher values
Threshold	0.9
Influencing_factors	Targeted approach and motivation of recruitable patients/persons in the organizational units; personnel capacities in the organizational units; willingness of patients/persons to participate; severity of the disease (recruitment rate decreases with increasing severity of the disease and thus increasing effort of the survey).
Notes_calculation	Alternative calculation options: 1) Determine the expected variability of the recruitment rate per selected time interval 2) Determine the current recruitment rate (number of patients/persons per time interval) in the selected time interval 3) Deviation = recruitment rate in the previous time interval - recruitment rate in the current time interval. Absolute amount of the deviation > expected variability is an indication of recruitment problems. Derive an estimate for the completeness of the dataset from a sample and extrapolate this estimate to the entire project [Goldberg et al. 1980]. An incidence of prevalence rate known from historical data is used to calculate an expected number of cases for the project data. The difference between the expected number of cases and the actual number of cases found in the database is a measure of completeness [Goldberg et al. 1980, Kuntoro et al. 1994]. To increase the significance of the indicator, the severity of the illness and the difficulty in obtaining patient consent (e.g. for complex, possibly painful examinations) should be taken into account in the calculation.

Section Assessment

Interpretation	The higher the rate/ratio, the better the data quality. A recruitment rate < threshold value indicates that too small a proportion of the recruitable patients/persons were actually included in the data collection. The completeness is insufficient.
Notes_assessment	

IDEFIM's Set of Quality Indicators - Specifications

Refusal rate of investigations

IDEFIM-1027

**Section administration**	
Sequence	
Designation	Refusal rate of investigations
Designation_inverse	Acceptance rate of investigations
Label	RefRateInves
Identificator_QI_IDEFIM	IDEFIM-1027
Identificator_external	TMF-1031
URI	
Description	Proportion of refused examinations.
Description_terminology	
Structure_superordinate	Completeness (data)
Status	release
Version	0.80
Last_update	2014
Contact	Prof. Dr. med. J. Stausberg (juergen.stausberg@uk-essen.de), S. Harkener (sonja.harkener@uk-essen.de)
Concept_upper	Refusal rate indicators
Concept_sub	The indicator is a sub-category of 'refusal rates'.
Sources	TMF guideline 2014: Study in Health in Pomerania (SHIP, TMF project V020-04).
General_notes	The indicator is related to indicator TMF-1012 'Missing modules'. The prerequisite for determining the indicator is the possibility of explicitly documenting a refusal of examinations; this indicator can be determined both for a specific examination, e.g. MRI, and for various examinations together.
**Section References**	
Property	The calculation is performed for a dataset.
**Section Calculation**	
Calculation_method	1) Identification of the data element in which the refusal is recorded for each examination type. 2) Determination of the value indicating a denial. 3) Search for the occurrence of this value 4) Calculation of numerator, denominator and rate.
Measurement_function	Numerator: Number of refused examinations. Denominator: Number of analyses reviewed.
Result_specification	rate (without dimension)
Direction	lower values
Threshold	
Influencing_factors	Study design, incentive for study participants, burden on study participants.

Refusal rate of investigations

IDEFIM-1027

Notes_calculation	

Section Assessment

Interpretation	The higher the rate, the poorer the data quality. Which rate is still acceptable depends on the intended use of the data.
Notes_assessment	

IDEFIM's Set of Quality Indicators - Specifications

Refusal rate of modules

IDEFIM-1028

**Section administration**	
Sequence	
Designation	Refusal rate of modules
Designation_inverse	Acceptance rate of modules
Label	RefRateMod
Identificator_QI_IDEFIM	IDEFIM-1028
Identificator_external	TMF-1032
URI	
Description	Proportion of modules refused.
Description_terminology	
Structure_superordinate	Completeness (data)
Status	release
Version	0.80
Last_update	2014
Contact	Prof. Dr. med. J. Stausberg (juergen.stausberg@uk-essen.de), S. Harkener (sonja.harkener@uk-essen.de)
Concept_upper	Refusal rate indicators
Concept_sub	The indicator is a sub-category of 'refusal rates'.
Sources	TMF guideline 2014: Open European Nephrology Science Center (OpEN.SC, TMF project V020-04), Study in Health in Pomerania (SHIP, TMF project V020-04).
General_notes	The indicator is closely related to the indicators TMF-1012 'Missing modules' and TMF-1031 'Refusal rate of examinations'. The prerequisite for determining the indicator is the possibility of explicitly documenting a refusal of modules.
**Section References**	
Property	The calculation is performed for a dataset.
**Section Calculation**	
Calculation_method	1) Identification of the data element in which the denial is recorded. 2) Determination of the value that indicates a denial. 3) Search for the occurrence of this value. 4) Calculation of numerator, denominator and rate.
Measurement_function	Numerator: Number of modules refused. Denominator: Number of modules checked.
Result_specification	rate (without dimension)
Direction	lower values
Threshold	
Influencing_factors	Study design, incentive for study participants.
Notes_calculation	

Refusal rate of modules

IDEFIM-1028

Section Assessment

Interpretation	The higher the rate, the poorer the data quality. Which rate is still acceptable depends on the intended use of the data.
Notes_assessment	

IDEFIM's Set of Quality Indicators - Specifications

Refusal rate of single data elements

IDEFIM-1029

Section administration

Sequence	
Designation	Refusal rate of single data elements
Designation_inverse	Acceptance rate of single data elements
Label	RefRateDE
Identificator_QI_IDEFIM	IDEFIM-1029
Identificator_external	TMF-1033
URI	
Description	Proportion of refused data elements.
Description_terminology	
Structure_superordinate	Completeness (data)
Status	release
Version	0.80
Last_update	2014
Contact	Prof. Dr. med. J. Stausberg (juergen.stausberg@uk-essen.de), S. Harkener (sonja.harkener@uk-essen.de)
Concept_upper	Refusal rate indicators
Concept_sub	The indicator is a sub-category of 'refusal rates'.
Sources	TMF guideline 2014: Study in Health in Pomerania (SHIP, TMF project V020-04).
General_notes	The indicator is complementary to the indicator TMF-1013 'Missing values for data elements' and its subcategories TMF-1014 and TMF-1015. The prerequisite for determining the indicator is the possibility of explicitly documenting a refusal to answer data elements. The data elements will usually be elements of a survey, hardly an isolated parameter from a technical investigation.

Section References

Property	The calculation is performed for a dataset.

Section Calculation

Calculation_method	1) Identification of data elements for which a denial is recorded. 2) Determination of the value that indicates a denial. 3) Search for the occurrence of this value. 4) Calculation of numerator, denominator and rate.
Measurement_function	Numerator: Number of values with refusal. Denominator: Number of checked values.
Result_specification	rate (without dimension)
Direction	lower values
Threshold	
Influencing_factors	Sensitivity of the data element, type of survey, interviewer

Refusal rate of single data elements

IDEFIM-1029

	training.
Notes_calculation	

Section Assessment

Interpretation	The higher the rate, the poorer the data quality. Which rate is still acceptable depends on the intended use of the data.
Notes_assessment	

IDEFIM's Set of Quality Indicators - Specifications

Relevance of the dataset's descriptive information

IDEFIM-1074

Section administration

Sequence	
Designation	Relevance of the dataset's descriptive information
Designation_inverse	Irrelevance of the dataset's descriptive information
Label	RelevInform
Identificator_QI_IDEFIM	IDEFIM-1074
Identificator_external	
URI	
Description	Indicates the degree of relevance of the dataset’s descriptive information to the content of the dataset.
Description_terminology	
Structure_superordinate	Understandability
Status	draft
Version	0.80
Last_update	13.5.2025
Contact	Prof. Dr. med. J. Stausberg (juergen.stausberg@uk-essen.de), S. Harkener (sonja.harkener@uk-essen.de)
Concept_upper	Indicators for understandability
Concept_sub	
Sources	IDEFIM review: wu_2021.
General_notes	

Section References

Property	The calculation is performed for a dataset.

Section Calculation

Calculation_method	1) Compilation of a dataset's descriptive information. 2) Development or selection of a method to define the relevancy of information. 3) Comparison of the descriptive information with the metadata and the dataset using the method at hand.
Measurement_function	Decision about having irrelevant descriptive information as a sentinel event.
Result_specification	yes/no
Direction	not applicable
Threshold	
Influencing_factors	
Notes_calculation	

Section Assessment

Interpretation	
Notes_assessment	

IDEFIM's Set of Quality Indicators - Specifications

Representativeness

IDEFIM-1042

Section administration

Sequence	
Designation	Representativeness
Designation_inverse	Unrepresentativeness
Label	Represent
Identificator_QI_IDEFIM	IDEFIM-1042
Identificator_external	TMF-1048
URI	
Description	Representativeness of the data.
Description_terminology	Representativeness: The population recorded in the dataset is a sample of the total population to be analysed, which largely corresponds to the total population in terms of the key influencing variables.
Structure_superordinate	Representativeness
Status	release
Version	0.80
Last_update	2014
Contact	Prof. Dr. med. J. Stausberg (juergen.stausberg@uk-essen.de), S. Harkener (sonja.harkener@uk-essen.de)
Concept_upper	Indicators for representativeness
Concept_sub	
Sources	TMF guideline 2014: Project partner.
General_notes	Representativeness is important for the generalizability of evaluation results. However, no criteria are known from the literature that allow an objective measurement of the transferability of the results obtained from data to the corresponding population.The indicator is only relevant for datasets that are not intended to cover the entire target population, but only a representative part of the target population. Incidences or prevalences known from other sources (e.g. the proportion of drug-addicted HIV-infected persons in the total number of HIV-infected persons) can be used to determine the main influencing variables.

Section References

Property	The calculation is performed for a dataset.

Section Calculation

Calculation_method	1) Selection of the influencing variables essential for representativeness. 2) Determination of the influencing variables for the population of the dataset. 3) Comparison of the dataset population with the total population. 4) Calculation of numerator, denominator and rate.

Representativeness

IDEFIM-1042

Measurement_function	Numerator: Number of influencing variables with expected distribution. Denominator: Number of influencing variables analyzed.
Result_specification	rate (without dimension)
Direction	higher values
Threshold	
Influencing_factors	Type of organizational unit (e.g. hospital, general practitioner); spatial distribution of organizational units (e.g. large city, small town, village); different patient populations in different organizational units.
Notes_calculation	

Section Assessment

Interpretation	The higher the rate, the better the representativeness and thus the data quality. Which rate is still acceptable depends on the intended use of the data.
Notes_assessment	

IDEFIM's Set of Quality Indicators - Specifications

Residual classes of qualitative data elements

IDEFIM-1020

Section administration

Sequence	
Designation	Residual classes of qualitative data elements
Designation_inverse	Assignment to categories other than residual classes for qualitative data elements
Label	ResidClass
Identificator_QI_IDEFIM	IDEFIM-1020
Identificator_external	
URI	
Description	Proportion of values in residual classes of qualitative data elements.
Description_terminology	
Structure_superordinate	Precision
Status	release
Version	0.80
Last_update	23.12.2021
Contact	Prof. Dr. med. J. Stausberg (juergen.stausberg@uk-essen.de), S. Harkener (sonja.harkener@uk-essen.de)
Concept_upper	Indicators for precision
Concept_sub	
Sources	IDEFIM review: stausberg_2023_c.pdf.
General_notes	

Section References

Property	The calculation can be performed for a dataset. The calculation of the indicator only makes sense for qualitative data elements.

Section Calculation

Calculation_method	1) Identification of the data elements with a residual class. 2) Calculation of numerator, denominator and rate.
Measurement_function	Numerator: Number of values available in the dataset in residual classes for qualitative data elements. Denominator: Number of values available in the dataset for qualitative data elements for which there is a residual class as a selection option.
Result_specification	rate (without dimension)
Direction	lower values
Threshold	0.1 (REGISVF-AP)
Influencing_factors	Time available for data collection; qualification of the survey staff.
Notes_calculation	A residual class can be labelled 'other(s)', 'different', 'not in this list' or similar and applies if there is a specific answer but there is no category for it in the selection list. This is not to be confused

Residual classes of qualitative data elements

IDEFIM-1020

	with the 'unknown' selection option: If no value was entered for a data element with a residual class (=missing), this data element is not counted, even if coding was carried out for 'missing' and a value was therefore saved. Data elements with a residual class for which 'don't know', 'unknown' or similar were recorded are only included in the denominator; the value is not a residual class. There is another indicator for a separate check for these values (TMF-1016 Proportion of data elements with value unknown or similar).
**Section Assessment**	
Interpretation	The lower the rate, the better the answer options cover the need; if the rate is high, it should be checked whether the category 'residual class' was selected because no other answer actually applies or whether there were other reasons (e.g. lack of time because answer options are too complex; lack of knowledge to categorize correctly).
Notes_assessment	If it is possible to enter a free text after selecting the residual class, this could be evaluated regularly in order to add further response categories to the selection list if necessary (further development/optimization of the metadata). The handling of data elements (i.e. the consideration for this indicator) for which several values can be recorded (multiple selection), among which there is also a residual class, possibly also in a sequential order, must be considered and clarified separately.

IDEFIM's Set of Quality Indicators - Specifications

Responsiveness

IDEFIM-1063

Section administration	
Sequence	
Designation	Responsiveness
Designation_inverse	Unresponsiveness
Label	Responsiven
Identificator_QI_IDEFIM	IDEFIM-1063
Identificator_external	
URI	
Description	Ability of data elements to reflect changes.
Description_terminology	
Structure_superordinate	Accuracy (metadata)
Status	draft
Version	0.80
Last_update	13.5.2025
Contact	Prof. Dr. med. J. Stausberg (juergen.stausberg@uk-essen.de), S. Harkener (sonja.harkener@uk-essen.de)
Concept_upper	Indicators for accuracy (metadata)
Concept_sub	
Sources	IDEFIM review: couchoud_2013.
General_notes	
**Section References**	
Property	The calculation is performed for a dataset.
**Section Calculation**	
Calculation_method	1) Selection of data elements that capture different states of an observational unit over time. 2) Determination of an appropriate level of change sensitivity for each data element. 3) Counting the number of data elements that fulfil this level.
Measurement_function	Numerator: Number of data elements that are sensitive for change. Denominator: Number of data elements selected.
Result_specification	rate (without dimension)
Direction	higher values
Threshold	
Influencing_factors	
Notes_calculation	
**Section Assessment**	
Interpretation	
Notes_assessment	

IDEFIM's Set of Quality Indicators - Specifications

Richness (metadata)

IDEFIM-1067

Section administration

Sequence	
Designation	Richness (metadata)
Designation_inverse	Poverty (metadata)
Label	RichnMetd
Identificator_QI_IDEFIM	IDEFIM-1067
Identificator_external	
URI	
Description	Indicates the metadata richness of a dataset.
Description_terminology	
Structure_superordinate	Completeness (metadata)
Status	draft
Version	0.80
Last_update	13.5.2025
Contact	Prof. Dr. med. J. Stausberg (juergen.stausberg@uk-essen.de), S. Harkener (sonja.harkener@uk-essen.de)
Concept_upper	Indicators for completeness (metadata)
Concept_sub	
Sources	IDEFIM review: wu_2021.
General_notes	

Section References

Property	The calculation is performed for a dataset.

Section Calculation

Calculation_method	1) Counting the number of data elements for the whole dataset or a logical section of the dataset. 2) Alternatively, counting the number of used data elements for each observational unit considering the whole dataset or a logical section of a dataset.
Measurement_function	Using the count if analyzing the metadata directly; building the arithmetic mean or another appropriate distribution parameter in case of analyzing the dataset.
Result_specification	count
Direction	higher values
Threshold	
Influencing_factors	
Notes_calculation	

Section Assessment

Interpretation	
Notes_assessment	The more data elements a dataset contains, the higher its richness

Richness (metadata)

IDEFIM-1067

of metadata.

IDEFIM's Set of Quality Indicators - Specifications

Single data source per observational unit

IDEFIM-1035

Section administration

Sequence	
Designation	Single data source per observational unit
Designation_inverse	Multiple data sources per observational unit
Label	SourceObsU
Identificator_QI_IDEFIM	IDEFIM-1035
Identificator_external	TMF-1041
URI	
Description	Notifications from one or more data sources may exist for an observational unit. The indicator checks how often only a single data source exists for an observational unit.
Description_terminology	
Structure_superordinate	Consistency (data)
Status	release
Version	0.80
Last_update	2014
Contact	Prof. Dr. med. J. Stausberg (juergen.stausberg@uk-essen.de), S. Harkener (sonja.harkener@uk-essen.de)
Concept_upper	Other indicators for consistency (data)
Concept_sub	
Sources	TMF guideline 2014: Gesellschaft der epidemiologischen Krebsregister in Deutschland e.V. (GEKID) Hentschel/Katalinic 2008 (TMF project V020-04).
General_notes	Notifications from several data sources (e.g. from the general practitioner, hospital doctor and pathologist) may be available for a tumor case. If only a single data source is available for a case, important data may be missing because it was not recorded in this data source.

Section References

Property	The calculation can be performed for a single observational unit and for a dataset.

Section Calculation

Calculation_method	1) Determination of the number of data sources for each observational unit. 2) Determination of numerator, denominator and rate.
Measurement_function	Numerator: Number of observational units with only one data source. Denominator: Number of observational units checked.
Result_specification	rate (without dimension)
Direction	lower values
Threshold	

Single data source per observational unit

IDEFIM-1035

Influencing_factors	Motivation of the reporting offices to report, time of calculation.
Notes_calculation	The death certificate must not be included in the calculation.

Section Assessment

Interpretation	The higher the rate, the poorer the data quality.
Notes_assessment	

Synonyms (data)

IDEFIM-1031

Section administration

Sequence	
Designation	Synonyms (data)
Designation_inverse	Freedom from synonyms (data)
Label	SynonymData
Identificator_QI_IDEFIM	IDEFIM-1031
Identificator_external	TMF-1036
URI	
Description	Number of synonyms in observational units.
Description_terminology	Synonym: Lexical sign that has the same meaning as another lexical sign. In the context of datasets, patient identifiers are referred to as synonyms if they are lexically different but have been assigned to the same patient/person.
Structure_superordinate	Consistency (data)
Status	release
Version	0.80
Last_update	2014
Contact	Prof. Dr. med. J. Stausberg (juergen.stausberg@uk-essen.de), S. Harkener (sonja.harkener@uk-essen.de)
Concept_upper	Confusion and redundancy indicators
Concept_sub	
Sources	TMF guideline 2014: Parkin/Muir 1992, Winter et al. 2003.
General_notes	The indicator is important for the correct estimation of incidences and prevalences and is related to the indicator 'Number of duplicates in the database'. Unrecognised synonyms lead to patients/persons being counted more than once. The relationships between synonyms should be documented [Winter et al. 2003].

Section References

Property	The calculation is performed for a dataset. If possible, all patients/persons should be included in the search for synonyms.

Section Calculation

Calculation_method	1) Definition of the data elements (key elements) whose combination allows the recognition of patients/persons who are stored multiple times in the dataset. This can include, for example, the data elements used to generate the patient identifiers. Alternatively, record linkage procedures can be used. 2) Identification of synonyms by comparing the values of the key elements per patient/person. 3) Calculation of numerator, denominator and rate.

Synonyms (data)

IDEFIM-1031

Measurement_function	Numerator: Number of synonyms. Denominator: Number of patients/persons checked.
Result_specification	rate (without dimension)
Direction	lower values
Threshold	
Influencing_factors	Algorithm for generating patient identifiers; quality of the technical infrastructure (possibility of registering organizational unit changes and parallel treatment of a patient in several organizational units).
Notes_calculation	

Section Assessment

Interpretation	The higher the rate, the poorer the data quality. Which rate is still acceptable depends on the intended use of the data.
Notes_assessment	

IDEFIM's Set of Quality Indicators - Specifications

Synonyms (metadata)

IDEFIM-1071

Section administration

Sequence	
Designation	Synonyms (metadata)
Designation_inverse	Freedom from synonyms (metadata)
Label	SynonymMetd
Identificator_QI_IDEFIM	IDEFIM-1071
Identificator_external	
URI	
Description	Different designations are used for the same data element in multiple datasets.
Description_terminology	
Structure_superordinate	Consistency (metadata)
Status	draft
Version	0.80
Last_update	13.5.2025
Contact	Prof. Dr. med. J. Stausberg (juergen.stausberg@uk-essen.de), S. Harkener (sonja.harkener@uk-essen.de)
Concept_upper	Indicators for consistency (metadata)
Concept_sub	
Sources	IDEFIM review: quindroit_2023.
General_notes	Unlike synonyms (data) where observational units (patients/persons) are concerned, here the focus is on synonymous data elements in multiple datasets. For example: in the datasets 1 and 2, the patient object is represented by two distinct table names: PATIENT and PAT, respectively.

Section References

Property	The calculation is performed for mutiple datasets.

Section Calculation

Calculation_method	1) Search for data elements with different designations but describing the same content. 2) Calculation of numerator, denominator and rate.
Measurement_function	Numerator: Number of synonyms found. Denominator: Number of checked data elements.
Result_specification	rate (without dimension)
Direction	lower values
Threshold	
Influencing_factors	
Notes_calculation	

Synonyms (metadata)

IDEFIM-1071

Section Assessment

Interpretation

Notes_assessment

IDEFIM's Set of Quality Indicators - Specifications

Temporal missingness

IDEFIM-1080

Section administration

Sequence	
Designation	Temporal missingness
Designation_inverse	Temporal completeness
Label	TempMissing
Identificator_QI_IDEFIM	IDEFIM-1080
Identificator_external	
URI	
Description	Proportion of incomplete time series (i.e. missing values in data elements that have to be recorded at several points in time) in the dataset.
Description_terminology	
Structure_superordinate	Completeness (data)
Status	draft
Version	0.80
Last_update	27.6.2025
Contact	Prof. Dr. med. J. Stausberg (juergen.stausberg@uk-essen.de), S. Harkener (sonja.harkener@uk-essen.de)
Concept_upper	Missing content indicators
Concept_sub	
Sources	IDEFIM analysis: Giesa et al. 2025.
General_notes	

Section References

Property	The calculation can be performed for a data record, for a single observational unit and for a dataset.

Section Calculation

Calculation_method	1) Definition of time series 2) Search for incomplete time series. 3) Calculation of numerator, denominator and rate.
Measurement_function	Numerator: Number of incomplete time series. Denominator: Number of time series.
Result_specification	rate (without dimension)
Direction	lower values
Threshold	
Influencing_factors	
Notes_calculation	

Section Assessment

Interpretation	
Notes_assessment	

IDEFIM's Set of Quality Indicators - Specifications

Temporal trends in counts or proportions

IDEFIM-1052

Section administration

Sequence	
Designation	Temporal trends in counts or proportions
Designation_inverse	No temporal trends in counts or proportions
Label	TempTrends
Identificator_QI_IDEFIM	IDEFIM-1052
Identificator_external	
URI	
Description	Identification of temporal trends in the number or proportion of values of a data element within and between organizational units (e.g. study centers).
Description_terminology	
Structure_superordinate	Accuracy (data)
Status	draft
Version	0.80
Last_update	13.5.2025
Contact	Prof. Dr. med. J. Stausberg (juergen.stausberg@uk-essen.de), S. Harkener (sonja.harkener@uk-essen.de)
Concept_upper	Other indicators for accuracy (data)
Concept_sub	
Sources	IDEFIM review: brown_2013.
General_notes	

Section References

Property	The calculation is performed for a dataset.

Section Calculation

Calculation_method	1) Determination of data elements that should be examined. 2) Calculation of trends in counts or proportions. 3) Calculation of numerator, denominator and rate.
Measurement_function	Numerator: Number of data elements with conspicuous trends. Denominator: Number of data elements checked.
Result_specification	rate (without dimension)
Direction	lower values
Threshold	
Influencing_factors	
Notes_calculation	

Section Assessment

Interpretation	
Notes_assessment	

IDEFIM's Set of Quality Indicators - Specifications

Temporality of categorical data elements

IDEFIM-1060

Section administration

Sequence	
Designation	Temporality of categorical data elements
Designation_inverse	Permanence of categorical data elements
Label	TempCatDE
Identificator_QI_IDEFIM	IDEFIM-1060
Identificator_external	
URI	
Description	Evaluation of changes produced by recodification or renaming of categories or produced by protocol shifts that affects the precision by increasing or decreasing the number of categories.
Description_terminology	
Structure_superordinate	Consistency (data)
Status	draft
Version	0.80
Last_update	13.5.2025
Contact	Prof. Dr. med. J. Stausberg (juergen.stausberg@uk-essen.de), S. Harkener (sonja.harkener@uk-essen.de)
Concept_upper	Other indicators for consistency (data)
Concept_sub	
Sources	IDEFIM review: garcía-de-león-chocano_2015.
General_notes	The prerequisite for this indicator is that changes in the metadata (definition of value lists) or protocol changes occur during data collection.

Section References

Property	The calculation is performed for a dataset. The calculation of the indicator only makes sense for qualitative data elements.

Section Calculation

Calculation_method	1) Search for data elements, whose metadata has been changed due to recodification or renaming of categories or protocol shifts.<br>2) Calculation of numerator, denominator and rate.
Measurement_function	Numerator: Number of changed data elements. Denominator: Number of checked data elements.
Result_specification	rate (without dimension)
Direction	lower values
Threshold	
Influencing_factors	
Notes_calculation	

IDEFIM's Set of Quality Indicators - Specifications

Temporality of categorical data elements

IDEFIM-1060

Section Assessment

Interpretation

Notes_assessment

Validity

IDEFIM-1079

Section administration

Sequence	
Designation	Validity
Designation_inverse	-
Label	Validity
Identificator_QI_IDEFIM	IDEFIM-1079
Identificator_external	
URI	
Description	Harmonic mean of correctness and recall (F-Measure).
Description_terminology	
Structure_superordinate	Accuracy (data)
Status	draft
Version	0.80
Last_update	7.6.2025
Contact	Prof. Dr. med. J. Stausberg (juergen.stausberg@uk-essen.de), S. Harkener (sonja.harkener@uk-essen.de)
Concept_upper	Contingency table indicators
Concept_sub	
Sources	IDEFIM analysis: Proposal by a participant at an expert workshop.
General_notes	Validity or F-measure is a measure that takes into account both the precision and the recall of a model. Precision indicates how many of the items classified as positive by the model are actually positive (positive predictive value or correctness), while recall indicates how many of the actual positive items were correctly recognized as positive by the model (also called sensitivity).

Section References

Property	The calculation is performed for a dataset.

Section Calculation

Calculation_method	1) Definition of the data elements to be analyzed. 2) Calculation of correctness and sensitivity. 3) Calculation of f-measure.
Measurement_function	Numerator: 2 x correctness x recall. Denominator: correctness + recall.
Result_specification	rate (without dimension)
Direction	higher values
Threshold	
Influencing_factors	
Notes_calculation	

Section Assessment

Validity

IDEFIM-1079

Interpretation	The result of the F-measure is high only when both recall (sensitivity) and precision (correctness) are high. For example: It is 0 when no relevant documents have been retrieved, and is 1 if all retrieved documents are relevant and all relevant documents have been retrieved.
Notes_assessment	

IDEFIM's Set of Quality Indicators - Specifications

Values from external references

IDEFIM-1016

Section administration

Sequence	
Designation	Values from external references
Designation_inverse	Values that do not originate from external references
Label	ValFrExtRef
Identificator_QI_IDEFIM	IDEFIM-1016
Identificator_external	TMF-1020
URI	
Description	Percentage of values that match terms from external references (e.g. controlled vocabularies).
Description_terminology	
Structure_superordinate	Consistency (data)
Status	release
Version	0.80
Last_update	2014
Contact	Prof. Dr. med. J. Stausberg (juergen.stausberg@uk-essen.de), S. Harkener (sonja.harkener@uk-essen.de)
Concept_upper	Unexpected entry indicators
Concept_sub	
Sources	TMF guideline 2014: Open European Nephrology Science Center (OpEN.SC, TMF project V020-04).
General_notes	

Section References

Property	The calculation is performed for a dataset.

Section Calculation

Calculation_method	1) Definition of the external references (e.g. controlled vocabularies) to be considered. 2) Normalization of the entries or definition of a procedure for text comparison. 3) Search for values that match the terms from the external references (e.g. controlled vocabularies) 4) Calculation of numerator, denominator and rate.
Measurement_function	Numerator: Number of values with labels from external references (e. g. controlled vocabularies). Denominator: Number of checked values.
Result_specification	rate (without dimension)
Direction	higher values
Threshold	
Influencing_factors	Importance and degree of familiarity of external references (e.g. controlled vocabularies) for the data elements under consideration.

Values from external references

IDEFIM-1016

Notes_calculation	The calculation only makes sense for free text fields or data elements for which the list of permitted values can be supplemented during entry. It makes sense to subject the procedure used for text comparison to an explicit quality check.

Section Assessment

Interpretation	The lower the rate, the poorer the data quality. As a external references (e.g. controlled vocabulary) was not explicitly used when defining the data elements, a rate of 100% is not to be expected. The reasons relevant to this decision must be taken into account when interpreting the results.
Notes_assessment	
